# H3K36 trimethylation mediated by SETD2 regulates the fate of bone marrow mesenchymal stem cells

**DOI:** 10.1371/journal.pbio.2006522

**Published:** 2018-11-13

**Authors:** Lijun Wang, Ningning Niu, Li Li, Rui Shao, Huiling Ouyang, Weiguo Zou

**Affiliations:** 1 State Key Laboratory of Cell Biology, CAS Center for Excellence in Molecular Cell Science, Shanghai Institute of Biochemistry and Cell Biology, Chinese Academy of Sciences, University of Chinese Academy of Sciences, Shanghai, China; 2 State Key Laboratory of Oncogenes and Related Genes, Stem Cell Research Center, Renji Hospital, School of Medicine, Shanghai Jiao Tong University, Shanghai, China; Imperial College London, United Kingdom of Great Britain and Northern Ireland

## Abstract

During the aging process, bone marrow mesenchymal stem cells (BMSCs) exhibit declined osteogenesis accompanied by excess adipogenesis, which will lead to osteoporosis. Here, we report that the H3 lysine 36 trimethylation (H3K36me3), catalyzed by histone methyltransferase SET-domain-containing 2 (SETD2), regulates lineage commitment of BMSCs. Deletion of *Setd2* in mouse bone marrow mesenchymal stem cells (mBMSCs), through conditional Cre expression driven by *Prx1* promoter, resulted in bone loss and marrow adiposity. Loss of *Setd2* in BMSCs in vitro facilitated differentiation propensity to adipocytes rather than to osteoblasts. Through conjoint analysis of RNA sequencing (RNA-seq) and chromatin immunoprecipitation sequencing (ChIP-seq) data, we identified a SETD2 functional target gene, *Lbp*, on which H3K36me3 was enriched, and its expression was affected by *Setd2* deficiency. Furthermore, overexpression of lipopolysaccharide-binding protein (LBP) could partially rescue the lack of osteogenesis and enhanced adipogenesis resulting from the absence of *Setd2* in BMSCs. Further mechanistic studies demonstrated that the trimethylation level of H3K36 could regulate Lbp transcriptional initiation and elongation. These findings suggest that H3K36me3 mediated by SETD2 could regulate the cell fate of mesenchymal stem cells (MSCs) in vitro and in vivo, indicating that the regulation of H3K36me3 level by targeting SETD2 and/or the administration of downstream LBP may represent a potential therapeutic way for new treatment in metabolic bone diseases, such as osteoporosis.

## Author summary

During the aging process, the ability of the mesenchymal stem cells (MSCs) in the bone marrow to generate bone cells declines, and they become more likely to produce fat cells, resulting in brittle, or osteoporotic, bones. Here, we demonstrate that a histone modification—H3 lysine 36 trimethylation (H3K36me3), which is mediated by the enzyme SET-domain-containing 2 (SETD2) and regulates gene expression—plays an essential role in MSC lineage commitment in the bone marrow. We deleted the *Setd2* gene in bone marrow mesenchymal stem cells (BMSCs) in mice and found that these mice exhibited reduced bone formation and increased marrow fat accumulation. Notably, we identified *Lbp* as one of SETD2 functional downstream genes. Overexpression of lipopolysaccharide-binding protein (LBP) could partially rescue the phenotype of SETD2 deficiency in bone marrow stem cells. Further mechanistic studies revealed that H3K36me3 could regulate Lbp transcription by modulating its transcriptional initiation and elongation. Thus, our study provides a new mechanism for MSC fate determination and a therapeutic target for the treatment of osteoporosis.

## Introduction

Bone marrow mesenchymal stem cells (BMSCs) are defined as a subset of PDGFRα^+^Sca1^+^CD45^−^TER119^−^ cells from adult mouse bone marrow in vivo [1]. BMSCs are multipotent progenitors that can differentiate into several distinct cell types, including osteoblasts and adipocytes. During the aging process, BMSCs exhibit an age-dependent decline in osteogenesis accompanied by an elevated propensity toward adipogenesis [2–4]. This switch decreases the number of osteoblasts and increases the number of adipocytes, contributing to osteoporosis [5,6]. Over the past decades, significant progress has been made in understanding the transcriptional control of BMSC lineage commitment, especially the lineage switch between adipocytes and osteoblasts. However, the epigenetic regulation of this process remains to be further investigated.

Histone modifications represent a large proportion of epigenetic regulations—including histone methylation, acetylation, phosphorylation, ubiquitination, sumoylation, etc.—on different histone residues [7]. The histone methylation state has been revealed as an important modulator of BMSC differentiation into osteogenic lineage or adipogenic lineage. It has been reported that repressive histone modification H3K27 trimethylation regulated by enhancer of zeste homolog 2 (EZH2) and KDM6A/B facilitate bone marrow adipogenesis in vitro [8–10]. H3K9 trimethylation has also been reported to promote adipogenesis and repress osteogenesis in human mesenchymal stem cells (hMSCs), owing to the KDM4B depletion [10]. Most of these reports were done in cultured human or mouse BMSCs (mBMSCs), and little is known about the in vivo effects of the histone modifications on the fate determination of BMSCs.

SET-domain-containing 2 (SETD2) is a methyltransferase responsible for H3 lysine 36 trimethylation (H3K36me3) [11]. H3K36me3 is positively correlated with actively transcribed genes and has been shown to be more enriched on transcriptionally activated genes [12,13]. H3K36me3 may be involved in some specific process such as DNA repair or transcriptional initiation and elongation, and it may contribute to the composition of heterochromatin in combination with other histone modifications [12–16]. Physiologically, SETD2 plays an important role in tumor suppression, aging delay, and antiviral immunity [17,18]. However, the function of H3K36me3 and SETD2 in BMSC cell fate determination remains unclear. Recently, H3K36M mutation has been suggested to be involved in chondroblastoma, indicating that the modification of H3K36 may play a role in cell differentiation [19]. It has been reported that knockdown of SETD2 by short hairpin RNA (shRNA) in hMSCs could impair osteoblast differentiation in vitro as well [20].

Here, we demonstrate that H3K36me3 mediated by SETD2 plays an essential role in MSC lineage commitment. We genetically deleted SETD2 in BMSCs by crossing *Setd2*^*fl/fl*^ mice with *Prx1-cre* mice [21] to explore the role of H3K36me3 in MSC fate in vivo and found that *Setd2*-deficient mice (*Prx1-Cre*, *Setd2*^*fl/fl*^ mice) exhibited reduced bone formation and increased marrow fat accumulation. Notably, *Lbp* was identified as one SETD2 functional downstream genes. Overexpression of lipopolysaccharide-binding protein (LBP) could partially rescue the phenotype of SETD2 deficiency in BMSCs. Further mechanistic studies showed that H3K36me3 could regulate Lbp transcription level by modulating its transcriptional initiation and elongation. Thus, our study provides a new mechanism for MSC fate determination and therapeutic target for osteoporosis.

## Results

### H3K36me3 level changes accordingly with the SETD2 level in adipogenesis and osteogenesis of BMSCs

**Fig 1:**
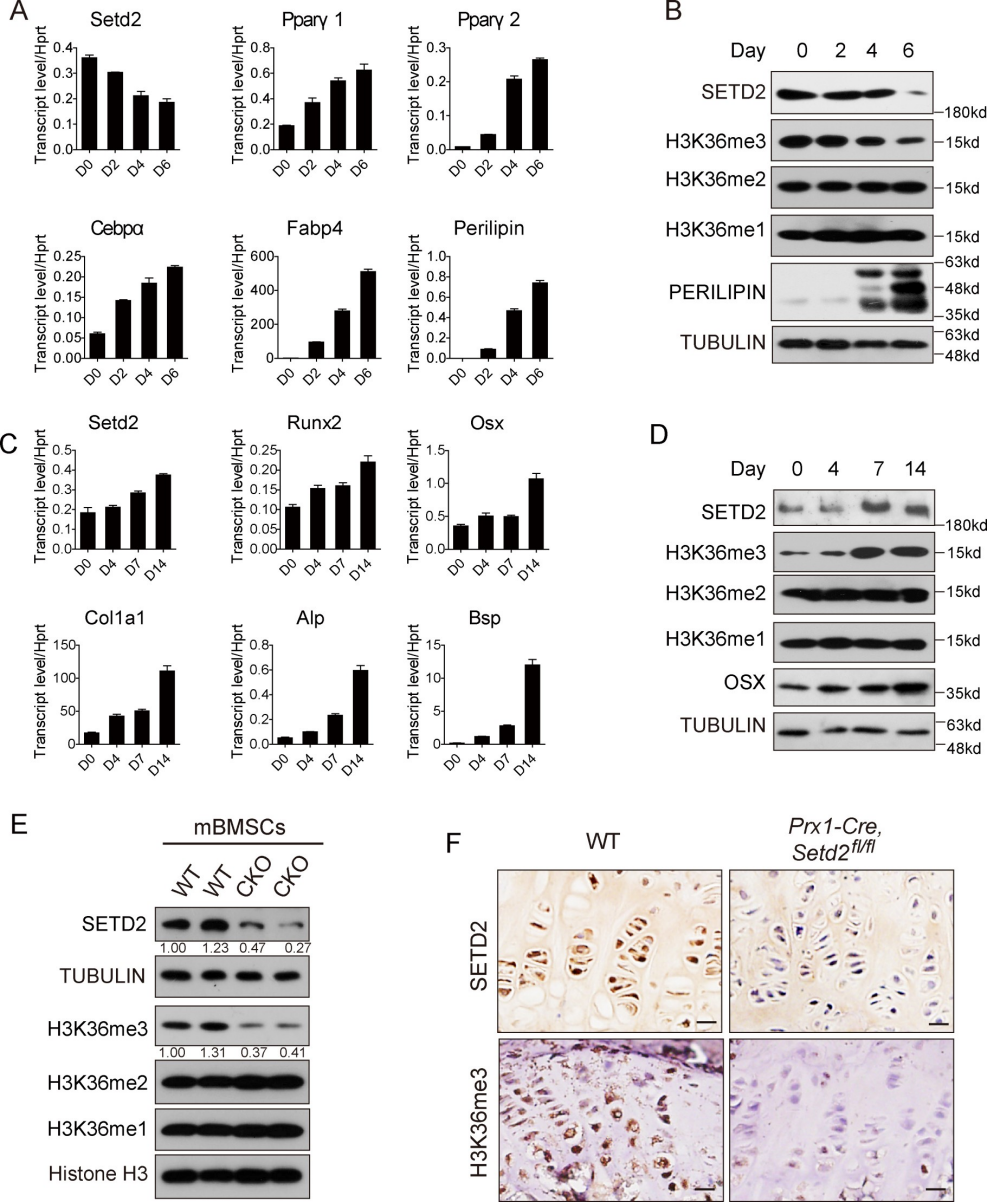
H3K36me3 changed accordingly with SETD2 level during BMSC adipogenesis and osteogenesis. (A) qPCR analysis of *Setd2* and adipocyte markers including *Ppar*γ*1*, *Pparγ2*, *Cebpα*, *Fabp4*, and *Perilipin* expression in BMSCs during adipogenesis for indicated days. Data represent mean ± SD, *n =* 4. (B) Western blot analysis of SETD2, PERILIPIN, and H3K36me1/2/3 level during adipogenesis for different days. (C) qPCR analysis of *Setd2* and osteoblast markers including *Runx2*, *Osx*, *Col1α1*, *Alp*, and *Bsp* expression during osteogenesis for different days. Data represent mean ± SD, *n* = 4. (D) Western blot analysis of SETD2, OSX, and H3K36me1/2/3 level in BMSCs during osteogenesis for indicated days. (E) Western blot analysis of SETD2 and H3K36me1/2/3 level in BMSCs isolated from WT and *Prx1-Cre*, *Setd2*^*fl/fl*^ mice. (F) Immunohistochemistry assay of SETD2 and H3K36me3 level in hindlimb growth plate of *Prx1-Cre*, *Set*^*d2fl/fl*^ mice and WT control mice. Images are representative results from 3 independent experiments. Scale bar = 20 μm. Data used in the generation of this figure can be found in S1 Data. Alp, alkaline phosphatase; BMSC, bone marrow mesenchymal stem cell; Bsp, bone sialoprotein; Cebpα, CCAAT enhancer binding protein α; Col1α1, Collagen type 1 alpha 1; Fabp4, fatty acid binding protein 4; H3K36me3, H3 lysine 36 trimethylation; Osx, Sp7 transcription factor; Pparγ1/2, peroxisome proliferative activated receptor γ1/2; qPCR, quantitative PCR; Runx2, runt-related transcription factor 2; SETD2, SET-domain-containing 2; WT, wild-type.

SETD2 has been reported as a major methyltransferase responsible for H3K36me3 [12]. In order to examine the role of H3K36me3 on mesenchymal cell fate in vivo, we firstly examined the SETD2 expression level and H3K36me3 level during BMSC differentiation to adipocytes and/or osteoblasts. Both SETD2 mRNA and protein levels are decreased during the course of adipocyte differentiation (Fig 1A and 1B). Meanwhile, the H3K36me3 level—but not H3K36me1 and H3K36me2 levels—is also decreased during the adipocyte differentiation (Fig 1A). On the contrary, mRNA and protein levels of SETD2 are elevated during the course of osteoblast differentiation, accompanied by the increase of H3K36me3 level and stable levels of H3K36me1 and H3K36me2 (Fig 1C and 1D). The dynamic alternations of SETD2 protein level and H3K36me3 during BMSC adipogenesis and osteogenesis indicate that H3K36me3 might serve as an MSC fate determinant.

To further examine the role of H3K36me3 in MSC lineage commitment in vivo, we generated a *Setd2* conditional knockout mice line. In *Setd2*-targeted allele, 2 *LoxP* sites were located at intron 5 and intron 7, and a *neomycin* resistance gene flanked with Frt sites was inserted into intron 7 and located between exon 7 and *LoxP* site (S1A Fig). The DNA of targeted embryonic stem cells (ESCs) was fragmented by Pst I restriction enzyme. For the correctly targeted allele, Southern blot using 3′-probe would detect an extra 5.5 k band as *Neomycin* gene carries a Pst I restriction enzyme site besides 2 other Pst I sites in intron 4 and 8 (S1A and S1B Fig). The identified ES cells were injected into 4- to 8-blastocyst embryos to get chimeric mice and then F0 targeted germ line mice. A PCR strategy was set up for genotyping the *Setd2*^*fl/fl*^ mice, in which the *Neomycin* resistance gene was removed by breeding with Flp recombinase mice (S1C Fig). To examine the MSC fate, we crossed *Setd2*^*fl/fl*^ mice with *Prx1-Cre* mice, which express *Cre* recombinase in the MSCs that form the limbs and parts of the skull, but not the spine or other organs [21]. Quantitative PCR (qPCR) using the primers targeting exon 6 revealed a reduction of SETD2 in BMSCs but not in liver (S1D Fig). Western blot confirmed the deletion of SETD2 in BMSCs but not in liver (Fig 1E and S1E Fig). More importantly, a reduction of H3K36me3 but not H3K36me1/2 was observed in SETD2-deleted BMSCs (Fig 1E). We also did immunohistochemistry assay and observed a complete loss of SETD2 in *Prx1-Cre-*positive cells in the tibia growth plate of *Prx1-Cre; Setd2*^*fl/fl*^ mice (Fig 1F, upper panel), accompanied with the decrease of H3K36me3 level (Fig 1F, lower panel). And the H3K36me1/2 did not change significantly (S1G Fig). The expression pattern of *Prx1-Cre* in the bone was further confirmed by breeding *Prx1-Cre* mice to the tdTomato reporter mice (a stop cassette flanked with *LoxP* sites before tdTomado gene, hereafter called Ai9 mice) in the femur [22]. As shown in S1F in S1 Fig, BMSCs and their progeny—including chondrocytes, osteoblasts, and adipocytes—were positive for tdTomato fluorescence in *Prx1-Cre*, *Ai9/+* mice, verifying the specific expression pattern of *Prx1-Cre*.

### SETD2 loss of function in BMSCs enhances adipogenic differentiation and impairs osteogeneic differentiation

**Fig 2:**
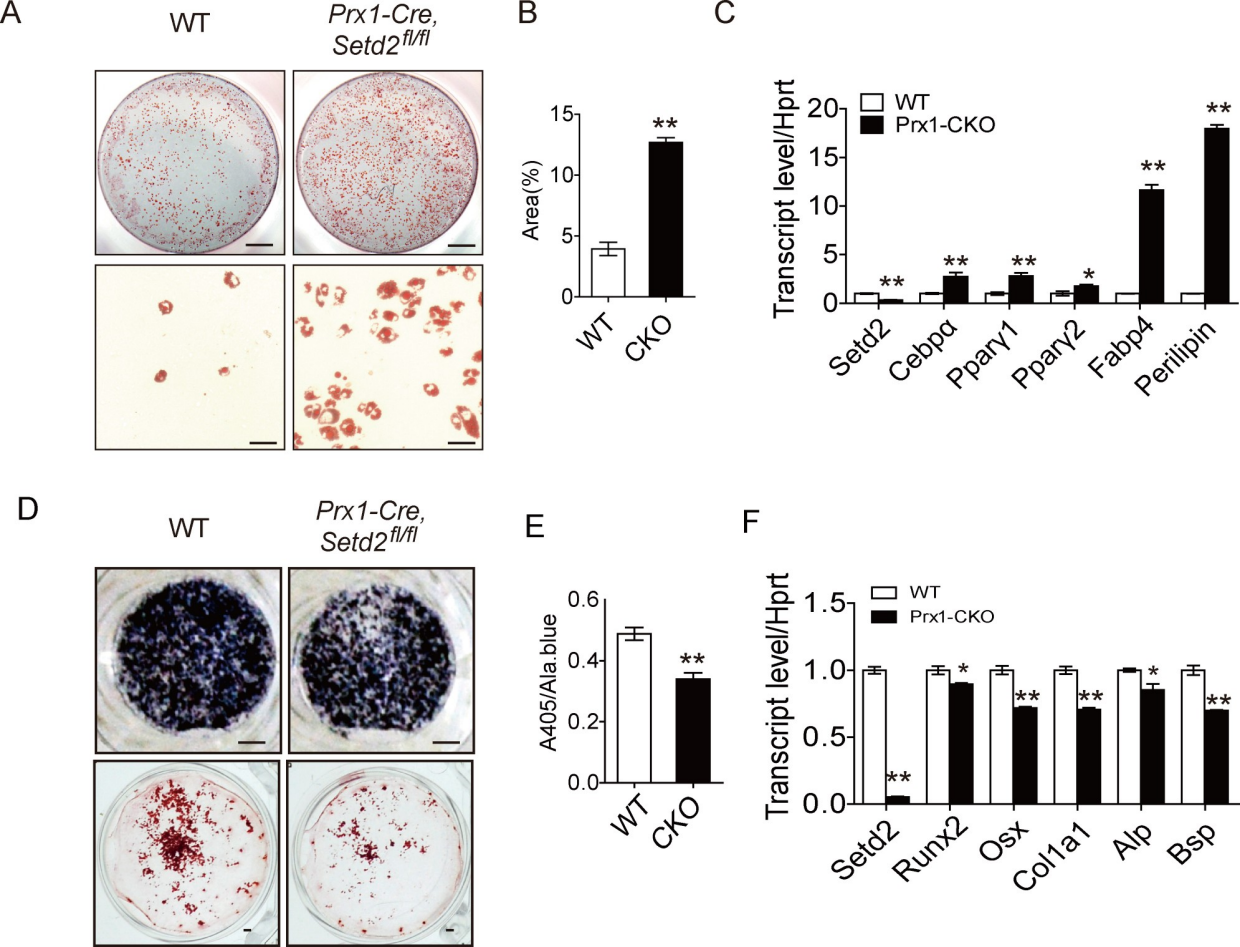
SETD2 loss of function enhances adipogenic differentiation and impairs osteogeneic differentiation. (A) Oil Red O staining of BMSCs after adipocyte differentiation for 6 days. Top panels: stained dishes, scale bar = 1 mm; lower panels: representative fields under the microscope, scale bar = 100 μm. Data are representative from 3 independent experiments. (B) Quantitative analysis of Oil Red O staining, *n =* 4. (C) qPCR analysis of *Setd2*, *Pparγ1*, *Pparγ2*, *Cebpα*, *Fabp4*, and *Perilipin* expression in BMSCs from WT and *Prx1-Cre*, *Setd2*^*fl/fl*^ mice after adipocyte differentiation for 6 days. Data represent mean ± SD, *n =* 4. (C) Alp staining and Alizarin red S staining after osteoblast differentiation for 7 days (upper) and 21 days (lower), respectively; scale bar = 1 mm. Data are representative from 3 independent experiments. (D) Alp activity quantification was measured by phosphatase substrate assay. Data represent mean ± SD, *n =* 4. (E) qPCR analysis of *Setd2*, *Runx2*, *Osx*, *Col1α1*, *Alp*, and *Bsp* expression after osteoblast differentiation for 7 days; cells were from WT and *Prx1-Cre*, *Setd2*^*fl/fl*^ mice. Data represent mean ± SD, *n =* 4. Data used in the generation of this figure can be found in S1 Data. Alp, alkaline phosphatase; BMSC, bone marrow mesenchymal stem cell; Bsp, bone sialoprotein; Cebpα, CCAAT enhancer binding protein α; Col1α1, Collagen type 1 alpha 1; Fabp4, fatty acid binding protein 4; Osx, Sp7 transcription factor; Pparγ1/2, peroxisome proliferative activated receptor γ1/2; qPCR, quantitative PCR; Runx2, runt-related transcription factor 2; SETD2, SET-domain-containing 2; WT, wild-type.

SETD2 deficiency leads to a decrease of H3K36me3 level, providing a unique tool to examine the role of H3K36me3 on BMSC fate. We examined the effects of SETD2 depletion on the adipocyte differentiation of BMSCs. BMSCs from *Prx1-Cre*, *Setd2*^*fl/fl*^ and wild-type (WT) mice were cultured in adipogenic medium for 6 days, and the lipid droplets were determined by Oil-red O staining. Then, we found that adipogenic differentiation was increased in *Setd2* knockout cells, as demonstrated by increased Oil-red O staining compared with cells from WT mice (Fig 2A and 2B), as well as the enhanced expression of characteristic adipocyte markers—including peroxisome proliferative activated receptor γ1/2 (*Pparγ1/2*), CCAAT/enhancer binding protein α (*C/EBPα*), fatty acid binding protein 4 (*Fabp4*), and *Perilipin* in adipocytes differentiated from *Prx1-Cre*, *Setd2*^*fl/fl*^ BMSCs compared to adipocytes differentiated from WT BMSCs (Fig 2C).

We next examined the role of SETD2 depletion in the osteoblast differentiation. Cells from *Prx1-Cre*, *Setd2*^*fl/fl*^, and WT mice were cultured in osteogenic medium for 1 week and 3 weeks as described in the Materials and methods section. Osteogenic differentiation was decreased in *Setd2* knockout cells as demonstrated by decreased alkaline phosphatase (ALP) activity (Fig 2D, upper panel and 2E: after 1-week culture) and decreased bone matrix formation determined by Alizarin red S staining (Fig 2E, lower panel: after 3-week culture). The declined osteogenesis in *Setd2* knockout cells was proved again by the decreased expression of a series of osteogenic marker genes (Fig 2F), including runt-related transcription factor 2 (*Runx2*), Sp7 transcription factor (*Osx*), Collagen type 1 alpha 1 (*Col1α1*), alkaline phosphatase (*Alp*), and bone sialoprotein (*Bsp*). Taken together, our data suggested that SETD2 regulates MSC fate by enhancing osteoblast differentiation and repressing adipocyte differentiation in vitro.

### SETD2-deficient mice show increased bone marrow adipocytes and decreased bone mass

**Fig 3:**
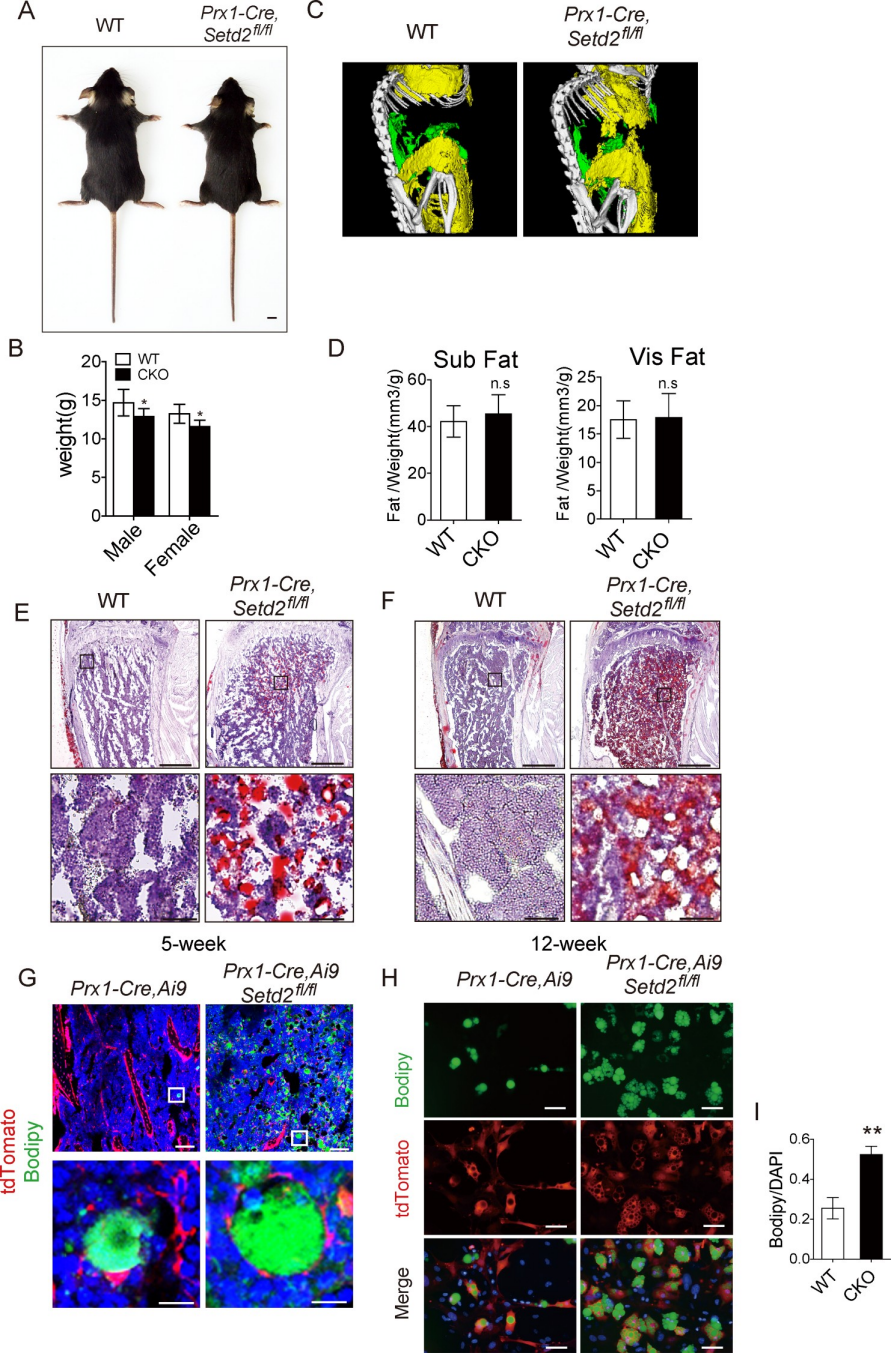
SETD2 loss of function in BMSCs showed increased bone marrow adipogenesis. (A) Gross images of 5-week-old *Prx1-Cre*, *Setd2*^*fl/fl*^ mice and its littermates. Images are representative of more than 6 mice per genotype, scale bar = 5 mm. (B) Weight of male and female mice was measured with 5-week-old mice. Results represent mean ± SD, male: *n =* 7; female: WT: *n =* 4, CKO: *n =* 6. (C) Analysis of white adipose tissue of *Prx1-Cre*, *Setd2*^*fl/fl*^ mice and its littermates; yellow means subcutaneous fat, and green means visceral fat. Results are representative of 3 repeats per group. (D) Quantitative analysis of subcutaneous fat and visceral fat. Results represent mean ± SD, *n =* 3. (E, F) Oil red O stains in distal tibia of *Prx1-Cre*, *Setd2*^*fl/fl*^ mice and WT control mice at the age of 5 weeks and 12 weeks. Images are representative of 3 mice per genotype. Scale bar = 1 mm (upper); scale bar = 100 μm (lower). (G) Lipid dye Bodipy 493/503 staining in distal femur of *Prx1-Cre*, *Setd2*^*fl/fl*^, *Ai9/+* mice and its control *Prx1-Cre*, *Ai9/+* mice at the age of 12 weeks. Images are representative of 3 mice per genotype; scale bar = 100 μm (upper); scale bar = 10 μm (lower). (H) Bodipy 493/503 staining of adipocytes differentiated from BMSCs isolated from *Prx1-Cre*, *Setd2*^*fl/fl*^, *Ai9/+* mice and its control *Prx1-Cre*, *Ai9/+* mice for 6 days. Images are representative from 3 independent experiments; scale bar = 100 μm. (I) Quantification of the ratio of differentiated adipocyte number to total BMSCs by Bodipy 493/503^+^ lipid drop/DAPI. Results represent mean ± SD, *n =* 4 random fields per condition. Data used in the generation of this figure can be found in S1 Data. BMSC, bone marrow mesenchymal stem cell; CKO, conditional knockout; SETD2, SET-domain-containing 2; WT, wild-type.

We further sought to examine the impact of altered BMSC differentiation on the intact animals. *Prx1-Cre*, *Setd2*^*fl/fl*^ mice were viable and fertile with a mild decreased body weight in both male and female mice (Fig 3A and 3B). However, the body fat store showed no significant difference between *Prx1-Cre*, *Setd2*^*fl/fl*^ mice and WT mice (Fig 3C and 3D). Consistent with increased adipogenic differentiation of *Setd2*-depleted BMSCs, 5-week-old Pr*x1-Cre*, *Setd2*^*fl/fl*^ mice displayed a significant adipocyte accumulation in bone marrow examined by Oil-red O staining (Fig 3E). Those increased bone marrow adipocytes were further accumulated in 12-week-old *Prx1-Cre*, *Setd2*^*fl/fl*^ mice (Fig 3F). We next asked whether the accumulated adipocytes were from *Prx1-Cre*-positive cells. We bred *Prx1-Cre*, *Setd2*^*fl/fl*^ with Ai9 mice and observed that adipocytes, labeled by lipid drop dye, bodipy 493/503, were colocalized with tdTomato fluorescence in both *Prx1-Cre*, *Ai9/+* mice and *Prx1-Cre*, *Ai9/+*, *Setd2*^*fl/fl*^ mice (Fig 3G), demonstrating that those accumulated bone marrow adipocytes in *Prx1-Cre*, *Setd2*^*fl/fl*^ mice were derived from *Prx1-Cre*-expressing cells in vivo. Consistently, in vitro adipogenic differentiation assay using BMSCs obtained from *Prx1-Cre*, *Ai9/+* mice and *Prx1-Cre*, *Ai9/+*, *Setd2*^*fl/fl*^ mice also proved that *Prx1-cre*-positive cells could turn into bodipy 493/503 positive adipocytes, and the lack of *Setd2* in BMSCs led to more adipocytes (Fig 3H and 3I).

**Fig 4:**
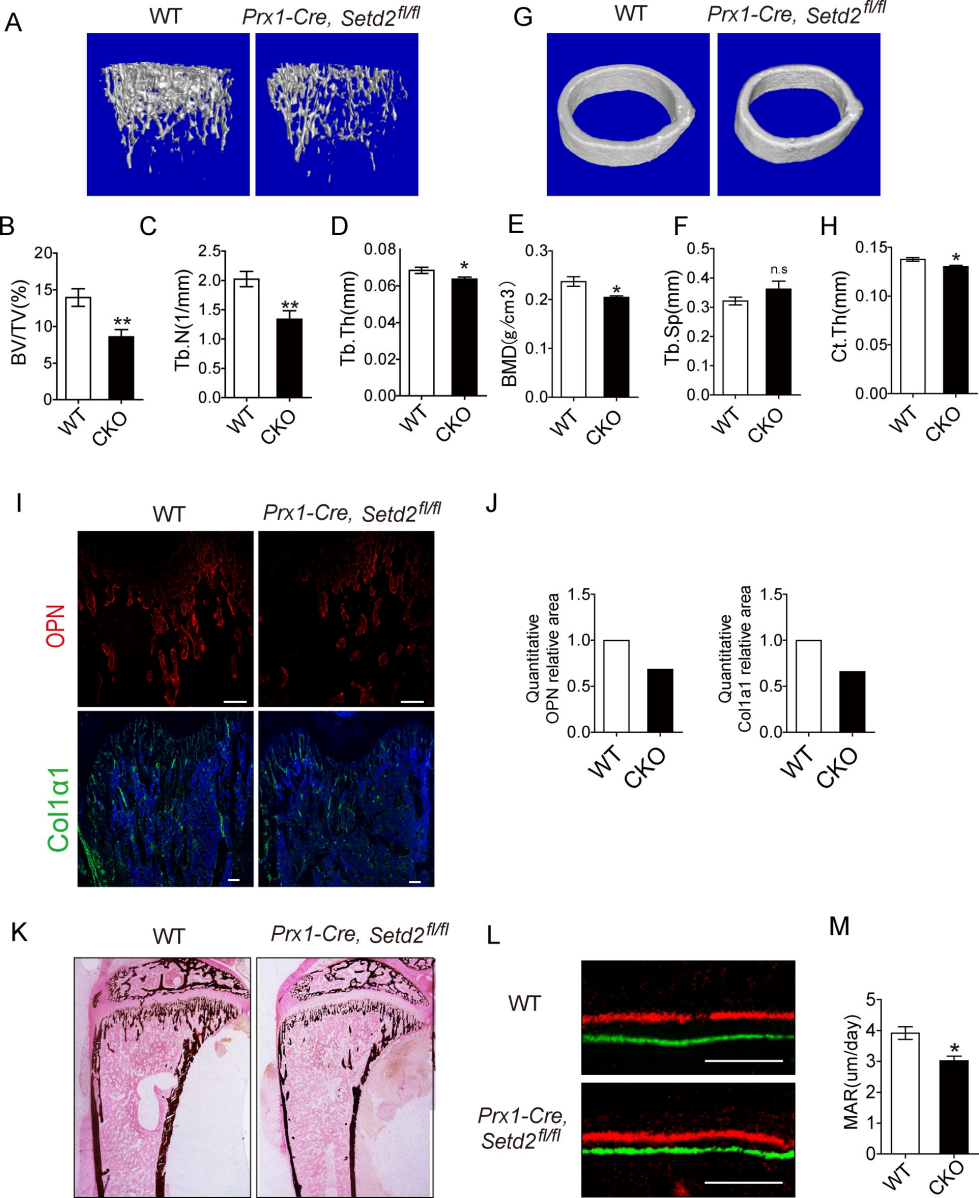
SETD2 loss of function in BMSCs showed decreased bone mass. (A) 3D μ-CT images of trabecular bone of distal femurs isolated from 5-week-old male WT and *Prx1-Cre*, *Setd2*^*fl/fl*^ mice. (B–F) μ-CT analysis of distal femur from 5 weeks old male WT and *Prx1-Cre*, *Setd2*^*fl/fl*^ mice for bone volume per tissue volume, trabecular number, trabecular thickness, BMD, and trabecular separation. Results are presented as the mean ± SD. (n ≥ 6 per group, significant difference by Student *t* test; *p < 0.05; **p < 0.01). (G) 3D μ-CT images of cortical bone of distal femurs isolated from 5-week-old male WT and *Prx1-Cre*, *Setd2*^*fl/fl*^ mice. (H) μ-CT analysis of cortical thickness (Ct.Th) of middle shaft of femur from 5-week-old male WT and *Prx1-Cre*, *Setd2*^*fl/fl*^ mice. (I) Immunofluorescence assay of Opn and Col1α1 of distal femur of 5-week-old WT and *Prx1-Cre*, *Setd2*^*fl/fl*^ mice. Scale bar = 100μm. (J) Quantification of the area of Opn and Col1α1 staining. The y-axis was normalized with the area of WT mice. (K) Representative images of Von kossa staining of 5-week-old WT and *Prx1-Cre*, *Setd2*^*fl/fl*^ mice. (L) Representative images of dual Calcein–Alizarin red S labeling of proximal tibia from WT and *Prx1-Cre*, *Setd2*^*fl/fl*^ mice. Scale bar = 100 μm. (M) Quantification of MAR. Results are presented as the mean ± SD, *n =* 3 per group. Data used in the generation of this figure can be found in S1 Data. BMD, bone mineral density; BMSC, bone marrow mesenchymal stem cell; BV/TV, bone volume per tissue volume; Col1α1, Collagen type 1 alpha 1; MAR, mineral apposition rate; Opn, osteopontin; SETD2, SET-domain-containing 2; Tb. N, trabecular number; Tb. Sp, trabecular space; Tb. Th, trabecular thickness; WT, wild-type.

We next examined the bone mass of *Prx1-Cre*, *Setd2*^*fl/fl*^ mice to test the hypothesis that *Prx1-Cre*, *Setd2*^*fl/fl*^ mice have decreased osteogenesis. We did micro-quantitative computed tomography (μ-QCT) analysis for the femurs isolated from *Prx1-Cre*, *SetD2*^*fl/fl*^ mice and WT control mice. The distal femurs of 5-week-old *Prx1-Cre*, *Setd2*^*fl/fl*^ mice exhibited reduced bone mass (Fig 4A), as evidenced further by significantly decreased trabecular bone volume (BV/TV) (Fig 4B), trabecular number (Tb. N) (Fig 4C), trabecular thickness (Tb. Th) (Fig 4D), and bone mineral density (BMD) (Fig 4E), accompanied by increased trabecular space (Tb. Sp) (Fig 4F). In addition to the trabecular phenotype, *Prx1-Cre*, *Setd2*^*fl/fl*^ mice also exhibit mild decreased thickness of cortical bone (Fig 4G and 4H). Consistent with μ-QCT analysis, immunofluorescence staining of distal femur of 5-week-old mice displayed decreased expression of osteoblast marker osteopontin (Opn) and Col1α1 in *Prx1-Cre*, *Setd2*^*fl/fl*^ mice (Fig 4I and 4J). Von-kossa staining showed decreased bone mineral deposition in *Prx1-Cre*, *Setd2*^*fl/fl*^ mice (Fig 4K). The mineral apposition rate (MAR) was also decreased in *Prx1-Cre*, *Setd2*^*fl/fl*^ mice compared with control WT littermates by fluorescent double labeling of the mineralizing front with Calcein and Alizarin red S (Fig 4L and 4M). Together, these results show that *Prx1-Cre*, *Setd2*^*fl/fl*^ mice have a phenotype consistent with enhanced adipogenesis and impaired osteogenesis in vitro, suggesting that SETD2 could regulate the fate of MSCs in vivo.

### SETD2 deficiency affects genome-wide gene expression and H3K36me3 level

**Fig 5:**
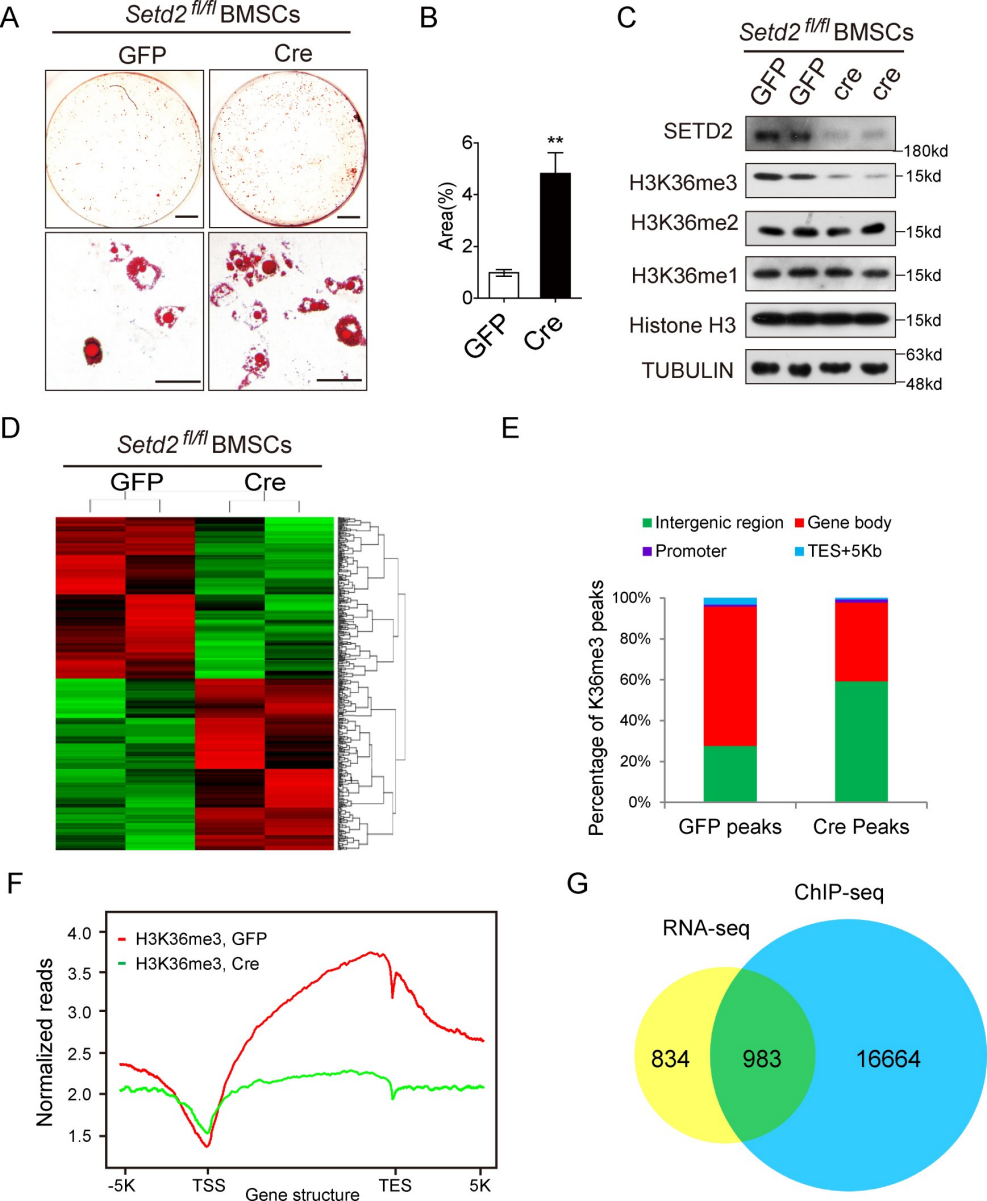
*Setd2* deficiency altered genome-wide H3K36me3 occupancy and downstream gene expression. (A) Oil Red O staining of BMSCs, which were isolated from *Setd2*^*fl/fl*^ mice and infected with lentivirus expressing GFP and Cre, induced by adipogenesis medium for 6 days. Scale bar = 1 mm (upper); scale bar = 100 μm (lower). (B) Quantitative analysis of Oil Red O staining area. Results are presented as the mean ± SD, *n =* 6. (C) Western blot analysis of SETD2 and H3K36me1/2/3 level in BMSCs isolated from *Setd2*^*fl/fl*^ mice and infected with lentivirus expressing GFP and Cre. (D) Heat map of RNA-seq data to compare the gene expression of BMSCs from control (*Setd2*^*fl/fl*^ BMSCs expressing GFP) and *Setd2*-knockout cells (*Setd2*^*fl/fl*^ BMSCs expressing Cre). The genes are ordered by clustering tightness. Each row represents a single RNA-seq data. (E) Analysis of the occupancy of H3K36me3 ChIP-seq peaks in gene bodies and intergenic regions. The lack of *Setd2* caused the decrease of peaks located in gene bodies and the increase in intergenic regions. (F) Normalized read density of H3K36me3 ChIP-seq signals of *Setd2*^*fl/fl*^ BMSCs expressing GFP or Cre from 5 kb upstream of the TSS to 5 kb downstream of the TES. (G) Venn diagram illustration of H3K36me3 peaks of *Setd2*^*fl/fl*^ BMSCs expressing GFP or Cre and their overlap with differential expression genes determined by RNA-seq. Data used in the generation of this figure can be found in S1 Data. BMSC, bone marrow mesenchymal stem cell; ChIP-seq, chromatin immunoprecipitation sequencing; GFP, green fluorescent protein; H3K36me3, H3 lysine 36 trimethylation; RNA-seq, RNA sequencing; SETD2, SET-domain-containing 2; TES, transcription end site; TSS, transcription start site.

We next sought to investigate the molecular mechanisms by which SETD2 regulates BMSC fate. To harvest enough cultured cells and directly assess the contribution of SETD2 to the lineage switch, we cultured BMSCs of *Setd2*^*fl/fl*^ mice and then infected the cells with lentivirus expressing green fluorescent protein (GFP) or Cre recombinase. *Setd2*^*fl/fl*^ BMSCs infected with Cre lentivirus showed more intensive adipogenesis (Fig 5A and 5B), consistent with elevated expression of adipocyte markers (S2A Fig). Reduced SETD2 expression accompanied with the decreased H3K36me3 level by Cre lentivirus expression was confirmed by western blot analysis (Fig 5C). We then performed RNA sequencing (RNA-seq) and H3K36me3 chromatin immunoprecipitation sequencing (ChIP-seq) analysis to identify SETD2-regulated genes (Series GSE120361). Next-generation sequencing using RNA from GFP or Cre lentivirus-infected *Setd2*^*fl/fl*^ BMSCs, respectively, revealed that the global transcriptome was changed dramatically between Cre-expressed cells compared to the GFP-expressed cells, indicating the significant function of SETD2 in BMSCs (Fig 5D). Among a total of 17,063 genes expressed, 904 genes were up-regulated, and 913 genes were down-regulated (fold change > 1.5) in SETD2-depleted cells. The expression levels of 13 genes were examined by qPCR and validated the RNA-seq data (S2B Fig). To further identify genes directly regulated by SETD2 and H3K36me3 on a genome-wide scale, we performed ChIP using an H3K36me3-specific antibody followed by next-generation sequencing (ChIP-seq) assays. A total of 23,001 and 147,216 H3K36me3 peaks were identified in SETD2-depleted BMSCs (*Setd2*^*fl/fl*^ cells expressing Cre) and control cells (*Setd2*^*fl/fl*^ cells expressing GFP), respectively. The reduction of H3K36me3 peaks in SETD2-deficient cells (Fig 5E and 5F, S3 Fig) certified that SETD2 is the major histone methytransferase of H3K36me3, consistent with the decrease of H3K36me3 level in SETD2-deficient cells determined by western blot analysis (Fig 5C). H3K36me3 was widely located in whole genomic regions, including gene bodies, promoters, 3′ UTR, and intergenic zones, except regions surrounding transcription start sites (TSSs) where the occupancy of H3K36me3 peaks were dramatically decreased (Fig 5E and 5F). SETD2 deficiency led to the decrease of H3K36me3 level at gene bodies and transcription end sites (TESs + 5 kilo base pairs [Kb]), indicating that SETD2 is responsible for the trimethylation on these regions (Fig 5E and 5F). Only a small percentage of H3K36me3 peaks resided in 5′ proximal regions (0–5 Kb), reflecting that H3K36me3 and SETD2 regulates gene transcription mainly through other regulation zones but not promoter regions (Fig 5E and 5F). We next analyzed H3K36me3 peaks that were lost in SETD2-deficient cells but present in control cells and found that the peaks were from 17,647 genes. We integrated the ChIP-seq data with the RNA-seq data and found that 983 genes (S1 Table) showed both the change of expression level and the loss of H3K36me3 peaks by SETD2 deficiency (Fig 5G), indicating that the altered H3K36me3 level on the genomic regions might regulate the expression levels of these genes.

### LBP downstream of SETD2 regulates the fate of MSCs

**Fig 6:**
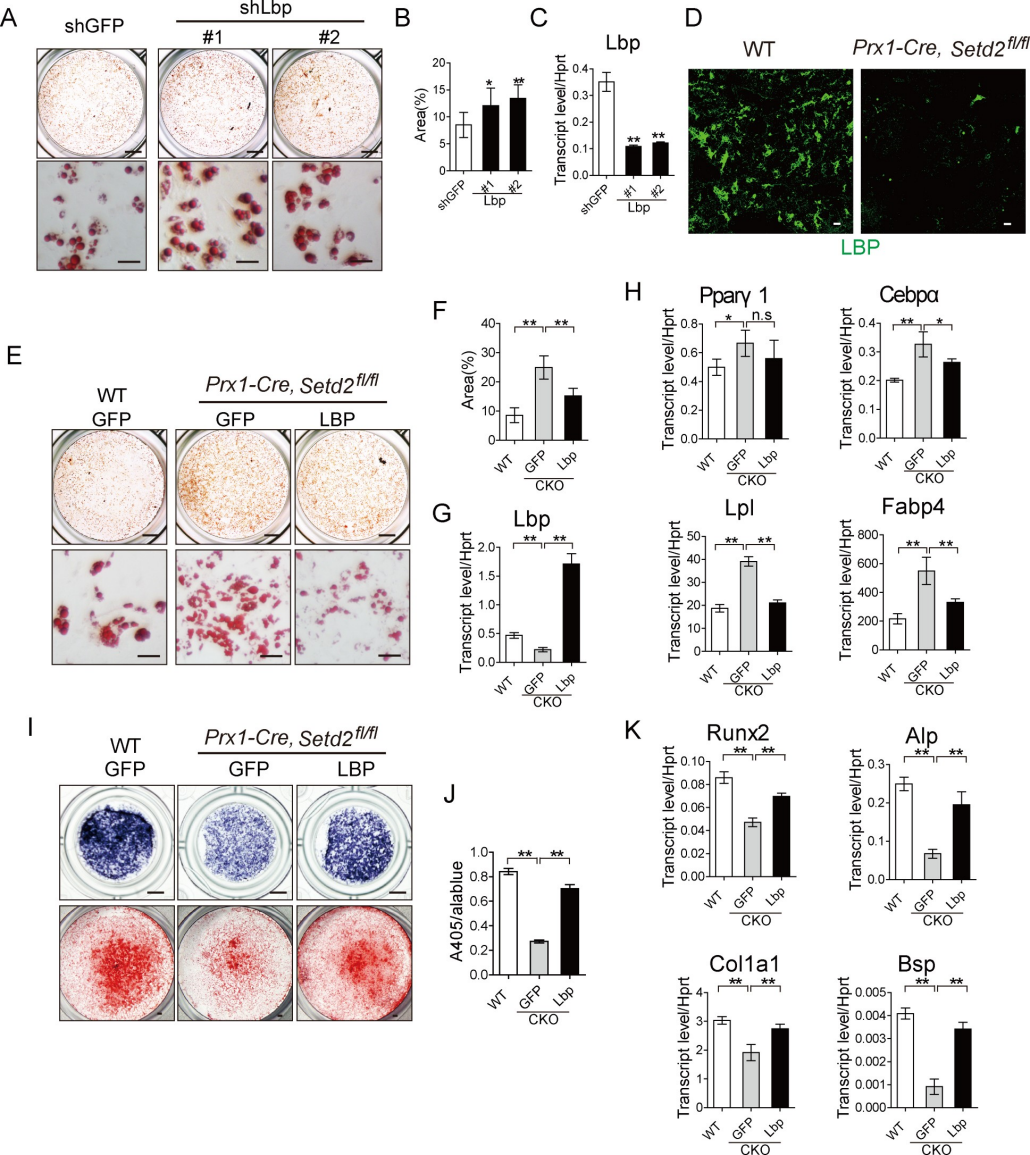
Enhanced LBP expression partially rescued the increased adipogenesis in *Setd2-*deficient BMSCs. (A) Morphological images of BMSCs infected with Lbp shRNA lentivirus induced by adipogenesis medium for 6 days; cells were stained with Oil Red O. Scale bar = 1 mm (upper); scale bar = 100 μm (lower). (B) Quantitative analysis of Oil Red O staining area. Results are presented as the mean ± SD, *n =* 7. (C) Knockdown efficiency of *Lbp* by qPCR analysis. (D) Immunofluorescence assay of LBP of distal femur of 5-week-old WT and *Prx1-Cre*, *Setd2*^*fl/fl*^ mice. Scale bar = 10 μm. (E) Morphological images of adipocyte differentiation at day 6 from BMSCs of WT and *Prx1-Cre*, *Setd2*^*fl/fl*^ mice infected with GFP and Lbp lentivirus. Differentiated adipocytes were stained with Oil Red O. Scale bar = 1 mm (upper); scale bar = 100 μm. (lower). (F) Quantitative analysis of Oil Red O staining area. Results are presented as the mean ± SD, *n =* 6. (G–H) Analysis of *Lbp*, *PPARγ1*, *Cebpα*, *Lpl*, and *Fabp4* via qPCR at day 6 of adipocyte differentiation from BMSCs of WT and *Prx1-Cre*, *Setd2*^*fl/fl*^ mice infected with GFP and Lbp lentivirus. (I) Differentiation was evaluated by Alp staining and by Alizarin red S staining after culture in osteoblast differentiation media for 7 days (upper) and 21 days (lower), respectively. Cells isolated from WT and *Prx1-Cre*, *Setd2*^*fl/fl*^ mice were infected with GFP and Lbp lentivirus. Scale bar = 1 mm. (J) Alp activity quantification was measured by phosphatase substrate assay, *n =* 4. (K) qPCR analysis of *Runx2*, *Col1α1*, *Alp*, and *Bsp* expression in day 7 of osteoblast differentiation from cells of WT and *Prx1-Cre*, *Setd2*^*fl/fl*^ mice. Results are presented as the mean ± SD, *n =* 4. Data used in the generation of this figure can be found in S1 Data. Alp, alkaline phosphatase; BMSC, bone marrow mesenchymal stem cell; Bsp, bone sialoprotein; Cebpα, CCAAT enhancer binding protein α; Col1α1, Collagen type 1 alpha 1; Fabp4, fatty acid binding protein 4; LBP, lipopolysaccharide-binding protein; Pparγ1/2, peroxisome proliferative activated receptor γ1/2; qPCR, quantitative PCR; shRNA, short hairpin RNA; WT, wild-type.

As our above data demonstrated that SETD2 deficiency could facilitate adipogenesis and suppress osteogenesis both in vitro and in vivo, we hypothesized that some of SETD2- and/or H3K36me3-regulated genes would affect BMSC fate determination. Previous studies have demonstrated that H3K36me3 could activate mRNA transcription initiation and elongation to increase transcription level [23,24]. Therefore, we focused on genes down-regulated by SETD2 deficiency in the previous sorted gene list (S1 Table). We also analyzed the expression pattern of those genes in BioGPS (http://biogps.org/) and focused on a list of genes that have been reported or predicted to be involved in adipocyte metabolism. Plasma long pentraxin 3 (PTX3) was reported to be associated with type 2 diabetes, human obesity, and metabolic syndrome [25–27]. LBP is a negative regulator of adipocyte differentiation and adipose tissue browning [28,29]. β-1,3-galactosyltransferase 2 (B3galt2) transfers galactose from UDP-galactose to substrates and is highly expressed in white and brown adipose tissue [30]. qPCR confirmed that the expression levels of these 3 candidates (*Ptx3*, *Lbp*, and *B3galt2*) were decreased in the bone marrow cells derived from *Prx1-Cre*, *Setd2*^*fl/fl*^ mice (S4A Fig). As a control, the expression level of some fundamental pathway-associated genes was stable (S4B Fig). We next sought to determine the effects of those targets on adipogenesis. The cultured BMSCs from 4- to 6-week-old mice were infected with lentivirus expressing those candidate genes. And overexpression of LBP—not PTX3 and B3GALT2—could restrain the differentiation of adipocytes accompanied with decreased expression of adipogenesis markers, including *Pparγ1*, *Cebpα*, *Lpl*, and *Fabp4*, determined by qPCR (S4C–S4E Fig). Consistently, Lbp knockdown with lentivirus expressing Lbp shRNAs promoted BMSC adipogenesis (Fig 6A–6C). Immunofluorescence staining of distal femur using LBP specific antibody confirmed decreased expression of LBP in the bone marrow of *Prx1-Cre*, *Setd2*^*fl/fl*^ mice (Fig 6D). Furthermore, the expression level of Lbp was decreased during adipogenesis and increased during osteogenesis, consistent with the changes of SETD2 expression (S4F and S4G Fig). To further determine the function of LBP, we cultured *Setd2*-deficient BMSCs and infected the cells with lentivirus expressing LBP. Overexpression of LBP could partially rescue the enhanced adipogenesis in SETD2-deficient cells, evidenced by the partially decreased Oil-red O staining (Fig 6E–6G). In addition, the expression level of adipocyte marker genes—including *Pparγ1*, *Cebpα*, *Lpl*, and *Fabp4*—determined by qPCR was also decreased in SETD2-deficient cells with LBP lentivirus treatment (Fig 6H). We next examined whether LBP could regulate the osteoblast differentiation. Meanwhile, overexpression of Lbp could partially rescue the impaired osteoblast differentiation resulting from loss of SETD2, which was determined by Alp activity and its quantification (Fig 6I, upper panel and 6J) as an early-stage osteoblast differentiation marker, and Alizarin red S staining (Fig 6I, lower panel) as a later-stage mineral deposition marker. In addition, the osteoblast markers—including *Runx2*, *Col1α1*, *Alp*, and *Bsp*—were also partially rescued in SETD2-deficient cells by Lbp lentivirus (Fig 6K).

Next, we administrated recombinant LBP in the osteogenic differentiation medium in WT mBMSCs for 7 days and harvested the cells for Alp staining, Alp quantification, Alizarin red S staining, and qPCR. We found that recombinant LBP could promote osteogenesis, as evidenced by increased Alp activity and Alizarin red S staining (S5A and S5B Fig). Consistently, the expression of osteoblast marker genes, including *Osx*, *Alp*, and *Bsp*, are also increased by LBP (S5C Fig). In addition, we also added recombinant LBP into the adipogenic differentiation medium in WT mBMSCs for 6 days, harvested the cells for Oil Red O staining, and performed RT-qPCR. Consistently, we found that recombinant LBP could repress adipogenesis, determined by decreased Oil Red O staining and the decrease of the expression of adipocyte marker genes, including *Pparγ1*, *Cebpα*, *fabp4*, and *Perilipin* (S5D–S5F Fig). Taken together, these data supported that LBP, whose expression level is regulated by SETD2, is a novel regulator of MSC fate.

### H3K36me3 mediated by SETD2 regulates transcriptional initiation and elongation of the Lbp gene

**Fig 7:**
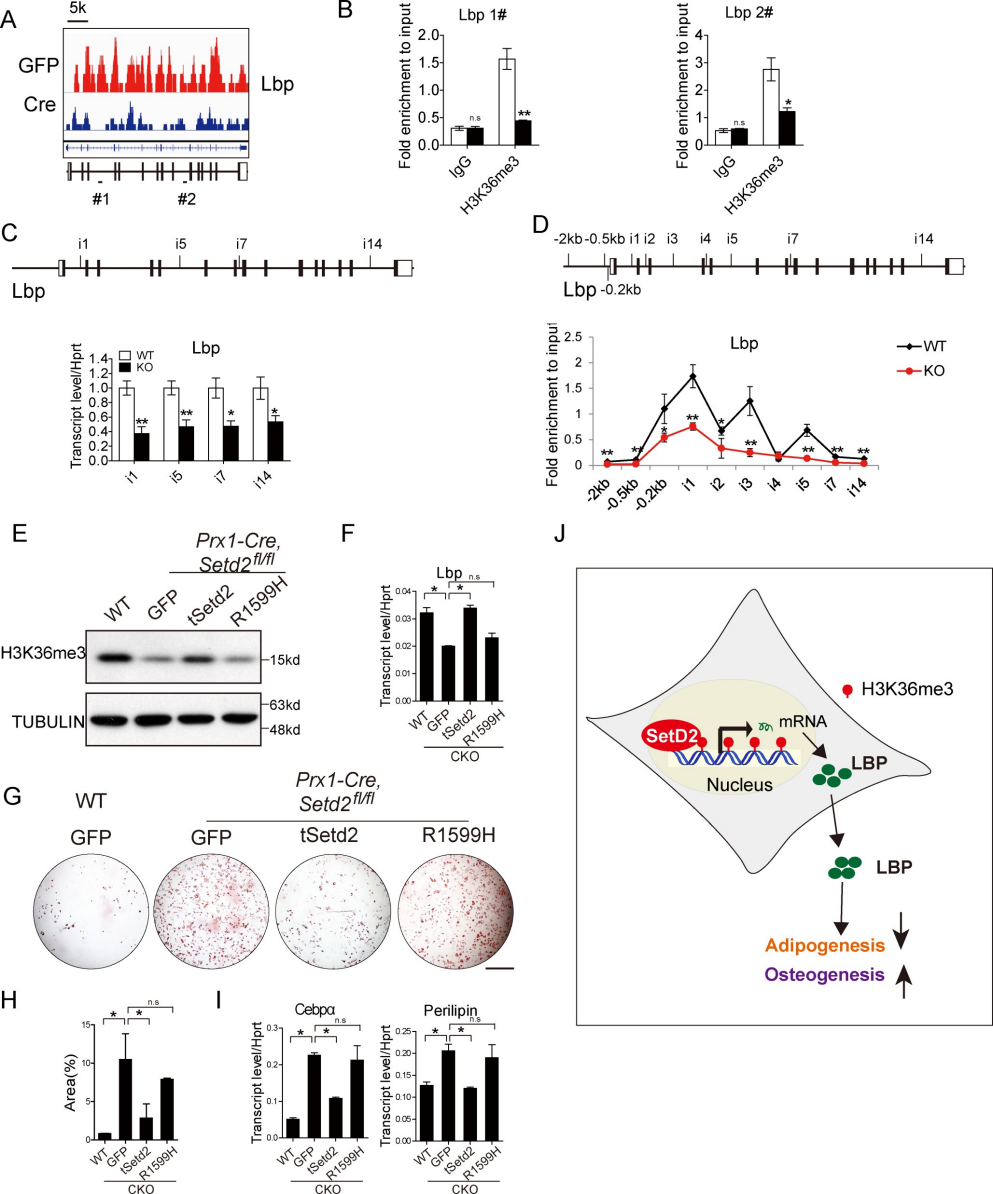
SETD2 regulates the transcription level of Lbp via altering the trimethylation level of H3K36. (A) ChIP-seq profiles of H3K36me3 occupancy to the locus of Lbp gene in BMSCs from *Setd2*^*fl/fl*^ BMSCs expressing GFP or Cre recombinase. Scale bar: 5 kb. All panels have the same signal scale of 0–5 RPM on the y-axis. (B) ChIP-qPCR analysis of H3K36me3 occupancy to the locus of Lbp gene, using IgG as the control. The location of primer #1 and #2 are indicated in the diagram of A. (C) qPCR analysis of the expression of Lbp heterogeneous nuclear RNA introns. The location of primers is indicated in the upper diagram. (D) ChIP-qPCR analysis of RNA polymerase II binding to the locus of Lbp genes. The location of primers for ChIP-qPCR (black bars) is indicated in the upper diagram. (E) Western blot of H3K36me3 in WT and *Setd2* KO BMSCs transfected with t*Setd2* WT and t*Setd2* R1599H mutation lentivirus. (F) qPCR analysis of *Lbp* in WT and *Setd2* KO BMSCs infected with WT t*Setd2* and t*Setd2* R1599H mutation lentivirus. Results are presented as the mean ± SD, *n =* 4. (G) Morphological images of adipocyte differentiation at day 6 from BMSCs of WT and *Prx1-Cre*, *Setd2*^*fl/fl*^ mice infected with GFP, WT t*Setd2* and t*Setd2* R1599H mutation lentivirus. Differentiated adipocytes were stained with Oil Red O. Scale bar = 1 mm. (H) Quantitative analysis of Oil Red O staining area. Results are presented as the mean ± SD, *n =* 3. (I) Analysis of *Cebpα*, *Perilipin* via qPCR at day 6 of adipocyte differentiation after indicated treatment. (J) Graphic model. H3K36me3 mediated by SETD2 regulates BMSC lineage commitment by promoting Lbp expression. Data used in the generation of this figure can be found in S1 Data. BMSC, bone marrow mesenchymal stem cell; Cebpα, CCAAT enhancer binding protein α; ChIP, chromatin immunoprecipitation; GFP, green fluorescent protein; H3K36me3, H3 lysine 36 trimethylation; IgG, immunoglobulin G; KO, knockout; Lbp, lipopolysaccharide-binding protein; qPCR, quantitative PCR; SETD2, SET-domain-containing 2; WT, wild-type.

We next sought to answer how SETD2 regulates Lbp expression. ChIP-seq analyses demonstrated that the genomic region of the Lbp gene was occupied by H3K36me3, and *Setd2* deficiency led to the decrease of H3K36me3 occupancy (Fig 7A). However, the H3K36me3 profiles showed much fewer changes on the fundamental pathway-associated gene regions (S6 Fig). The decreased H3K36me3 peaks were further confirmed by ChIP-qPCR analysis using H3K36me3-specific antibody (Fig 7B). To examine whether H3K36me3 occupancy of the Lbp gene could affect its mRNA transcriptional initiation and/or elongation, we analyzed the expression of the intron-containing nascent transcripts. The expression of almost every analyzed intronic region, from N-terminal to C-terminal, was decreased in BMSCs of *Prx1-Cre*, *Setd2*^*fl/fl*^ mice compared to WT control mice (Fig 7C). And then we performed RNA pol II ChIP assay followed by real-time PCR with primers covering different intronic regions of the Lbp gene (Fig 7D). As expected, the binding affinity of RNA pol II was reduced significantly at nearly all examined intronic regions, especially at −0.5 kb, upstream from the TSS, and intron1 of Lbp in BMSCs of *Prx1-Cre*, *Setd2*^*fl/fl*^ mice (Fig 7D). These data indicated that loss of H3K36me3 occupancy could affect RNA pol II binding affinity around TSS and gene body and thus impair both mRNA transcriptional initiation and elongation of Lbp.

To further confirm that the mBMSC lineage commitment was dependent on H3K36me3 mediated by SETD2, we generated a truncated WT *Setd2* (amino acid 1,297–2,537), which includes function domains for catalyzing H3K36me3, and a SET domain mutant of truncated *Setd2* (R1599H), which failed to trimethylate H3K36 [31]. Overexpression of t*Setd2* could not inhibit the adipogenesis of WT mBMSCs (S7A and S7B Fig). The possible reason is that the overexpression of SETD2 could not make excessive trimethylation on H3K36 in WT BMSCs (S7C Fig). As expected, overexpression of WT t*Setd2* and mutated t*Setd2* in SETD2-deficient BMSCs showed that the WT t*Setd2* instead of mutated t*Setd2* could rescue the level of H3K36me3 (Fig 7E). Consistently, the Lbp expression was up-regulated by overexpression of WT t*Setd2* rather than mutated *tSetd2* (Fig 7F). Furthermore, the adipogenesis was repressed by WT t*Setd2* instead of mutated t*Setd2* in *Setd2-*deficient BMSCs, as proved by the Oil Red O staining and the indicated adipocyte markers (Fig 7G–7I). These results implicated that the transcription initiation and elongation of the Lbp gene were regulated by H3K36me3 occupancy, mediated by SETD2.

## Discussion

In this study, we found that H3K36me3, mediated by histone methyltransferase SETD2, plays a key role in BMSC fate. SETD2 conventional knockout mice are embryonic lethal at E10.5–E11.5 due to severe vascular defects [32], and *Setd2* knockout ESCs have defects for differentiation toward endoderm [33]. Here, we constructed a conditional knockout mouse model to determine the physiological functions of H3K36me3 and SETD2 during BMSC lineage commitment. Our observation that SETD2 deficiency in BMSCs leads to the decrease of H3K36me3 but not mono- or dimethylation is consistent with the previous report that homozygous disruption of *Setd2* impaired H3K36me3 but not mono- or dimethylation [32], confirming that SETD2 is the major histone methyltransferase for H3K36me3.

Age-related osteoporosis is characterized by activated adipogenesis and suppressed osteogenesis in bone marrow stromal cells, leading to progressive fat accumulation and bone loss [34,35]. However, the regulations of this process remain to be further understood, especially in vivo epigenetic regulations. We observed that SETD2 deficiency in BMSCs led to reduced bone formation and accumulation of fat at a young age, resembling the phenotype of age-related osteoporosis. Although *Setd2* is also depleted in chondrocytes, *Setd2* deficiency barely affected the chondrocyte formation and differentiation in vivo and in vitro (S8 Fig). We found that *Setd2* and Lbp were decreased in the aged mBMSCs (S9 Fig), supporting our hypothesis that loss of *Setd2* could shift the lineage commitment of BMSCs to adipogenesis by repressing the expression of Lbp. These results are consistent with the report that H3K36 methylation promotes longevity [36]. And the SETD2-deficient mice might serve as a novel model to investigate age-related osteoporosis. Zhou and colleagues demonstrated that adipocytes function as the niche component that promote hematopoietic regeneration, while Ambrosi and colleagues established that adipogenic cells reduce hematopoietic reconstitution [37,38]. In our study, we observed excess adipocyte accumulation in the bone marrow of *Prx1-Cre; Setd2*^*fl/fl*^ mice. In order to determine whether the lack of *Setd2* in BMSCs could influence the hematopoiesis progress, we analyzed the occupation of LSK cells (Lin^−^ Sca1^+^ C-kit^+^ cells) and HSCs (CD150^+^ CD48^−^ Lin^−^ Sca1^+^ C-kit^+^ cells) in total bone marrow cells of 3 pairs of 6-month-old mice. However, we barely detected any significant impact on percentage of both LSK cells and HSCs by the accumulation of adipocytes in *Setd2*-deficient mice (S10 Fig). Thus, we speculated that the function of marrow adipocytes on HSCs might depend on the state and environment of the bone marrow, and the factors that adipocytes secreted, rather than the number of adipocytes only.

In past decades, the study of H3K36me3 and SETD2 mainly focus on their function as tumor suppressors, including clear cell renal cell carcinoma, chondroblastomas, lymphoma, acute leukemia, intestinal tumorigenesis, and so on [19,39–42]. Also, we found that the proliferation rate was increased mildly in *Setd2*-deficient BMSCs (S11 Fig). In the mechanism, Set2, H3K36 histone methyltransferase in yeast, has been reported to be required for transcription elongation [16]. Also, SETD2 could directly regulate transcriptional initiation of Fgfr3 through histone H3K36me3 modification [15]. In our study, *Setd2* could contribute to RNA pol II–mediated transcription initiation and elongation of Lbp. Recently, the function of SETD2 has been attributed to the methylation of nonhistone proteins, such as α-TUBULIN at lysine 40 and STAT1 at lysine 525 [43,44]. The function of nonhistone substrates mediated by SETD2 on MSC fate control need to be further determined.

Lysine methylation is one of the most important histone post-translational modifications that regulate chromatin structure. KDM4/JMJD2 proteins—containing KDM4A/JMJD2A, KDM4B/JMJD2B, KDM4C/JMJD2C and KDM4D/JMJD2D—are demethylases that target histone H3 on lysines 9 and 36 [45]. Demethylation of the repressive mark H3K9me3 by KDM4A could facilitate PPARγ promoter-specific binding and function in adipocytes [46]. However, KDM4C has been reported to reduce PPARγ transcriptional activity for PPARγ response element (PPRE) genes and reduce pre-adipocyte 3T3L-1 cell differentiation [47]. The reason for the opposite function on the PPARγ between the KDM4A and KDM4C remains unclear. Increase of H3K9 trimethylation by KDM4B depletion has been reported to promote adipogenesis and repress osteogenesis in hMSCs [10]. KDM4D is unique within the KDM4 family in that it lacks both the PHD and Tudor domains and thus is only half the size of KDM4A–C [48]. However, KDM4D has not been studied in bone and MSC lineage balance. Although several reports have been made to investigate the function of KDM4 on MSC lineage commitment and adipocyte differentiation in vitro, it is still worthwhile to pursue deep insight into their physiological significance.

In our study, we performed RNA-seq and ChIP-seq analysis and identified that SETD2 and H3K36me3 could regulate the transcription of a panel of different genes. Among these genes, LBP could partially rescue the phenotype of *Setd2*-deficient BMSCs. We also demonstrated that the lack of H3K36me3 on the Lbp gene body leads to less Pol II occupancy and decreased transcription initiation and elongation. LBP has been reported as an essential component of the innate immune responses to gram-negative bacterial infection and can protect murine liver from different injuries [49,50]. LBP knockdown using shRNA or anti-LBP antibody led to increased markers of adipogenesis and decreased adipocyte inflammation in human adipocytes [51]. In our study, overexpression of LBP could partially change the balance between osteoblasts and adipocytes, suggesting that the decrease of LBP in *Setd2*-deficient BMSCs is responsible to a certain degree for dysregulated fate determination of MSCs in *Prx1-Cre*, *Setd2*^*fl/fl*^ mice, indicating that circulating LBP is not only an immune regulator but also a regulator for cell fate and cell metabolism. It will be worthwhile to further investigate how Lbp regulates adipogenesis and osteogenesis. Lbp might function as a lipid-binding protein or adipokine to stimulate some specific signaling pathways. Furthermore, the specific Lbp receptor on the membrane of BMSCs remains to be elucidated.

Overall, our study identified mammalian SETD2 as a novel regulator of MSC fate and suggested that modulation of H3K36me3 could have a therapeutic potential in osteoporosis. Our study also identified LBP as a downstream target of SETD2. Considering that LBP is a secreted protein and that it can increase BMSC osteogenesis and decrease adipogenesis, LBPs might be an attractive reagent for rejuvenating our bone.

## Materials and methods

### Ethics statement

Animals were bred and maintained under specific pathogen free (SPF) conditions in the institutional animal facility of the Shanghai Institute of Biochemistry and Cell Biology, Chinese Academy of Sciences. All animal experiments were performed with protocol approved by the Animal Care and Use Committee of Shanghai Institute of Biochemistry and Cell Biology, Chinese Academy of Sciences (approval number: SIBCB-NAF-14-001-S350-019).

### Mouse lines

*Setd2*^*fl/fl*^ mice were crossed with the *Prx1-Cre* strain (a gift from Andrew McMahon, Harvard University) to generate *Prx1-Cre*, *Setd2*^*fl/fl*^ mice. All mice analyzed were maintained on the C57BL/6 background.

### Cell culture

BMSCs were extracted from femurs and tibiae of 4- to 6-week-old mice. Marrow plug was flushed with a 1-mL syringe. These plugs were then dispelled into single cell and seeded in 10-cm dish containing α-MEM (Corning, Corning, NY) supplemented with 10% fetal bovine serum (FBS). For osteoblast differentiation, cells were cultured in α-MEM containing 10% FBS, 50 μg/ml L-ascorbic acid, and 1,080 mg/mL β-glycerophosphate. The osteoblast differentiation level assay was performed following a previously published method [52]. For quantitative analysis of Alp activity, osteoblasts were incubated with Alamar Blue and were then incubated with phosphatase substrate (Sigma-Aldrich, St. Louis, MO) dissolved in 6.5 mM Na_2_CO_3_, 18.5 mM NaHCO_3_, 2 mM MgCl_2_. Alp activity was then read with a luminometer (Envision). Bone nodule formation was stained with 1 mg/mL Alizarin red S solution (pH 5.5) after 14 or 21 days of induction. For adipocytes, BMSCs were plated in 96-well plate at a density of 30,000 cells/well and stimulated in adipogenic induction medium (α-MEM/10% FBS containing 1 uM dexamethasone, 0.1 mM rosiglitazone, 0.5 mM IBMX, 10 ug/mL insulin) for 1 day followed by adipogenic maintenance medium (α-MEM/10% FBS containing 10 ug/mL insulin) for 2 or 3 days. After mature adipocyte formation, cells were stained with 2 mg/mL Oil red O solution or Bodipy 493/503.

Human embryonic kidney cell line (HEK293T) was obtained from ATCC and maintained in DMEM (Corning, Corning, NY) containing 10% FBS.

### Histology and immunohistochemistry

Hematoxylin–eosin stain and immunohistochemistry were performed as previously described [53]. Tissues were fixed in 4% paraformaldehyde for 48 hours and incubated in 15% DEPC-EDTA (pH 7.8) for decalcification. Then, specimens were embedded in paraffin and sectioned at 8 μm. Immunohistochemistry was performed using Vector Rabbit kit according to the manufacturer’s instructions with antibodies against SETD2 (1:200; LSBIO, LS-B12660-50), H3K36me3 (1:500; Abcam, ab9050). Images were captured using a microscope (Olympus BX51, Tokyo, Japan).

### Immunofluorescence

Immunofluorescence was performed as previously described [54]. Freshly dissected bones were fixed in 4% paraformaldehyde for 48 hours and incubated in 15% DEPC-EDTA (pH 7.8) for decalcification. Then, specimens were embedded in paraffin or OCT and sectioned at 8 μm. Sections were blocked in PBS with 10% horse serum for 1 hour and then stained overnight with donkey-anti-OPN (1:1000; R&D, AF808), rabbit-anti-Collagen I (1:100; Rockland, 600-400-103), and rabbit-anti-Lbp (1:50; R&D; 11836-1AP). Donkey-anti-goat cy3 (1:1000; Jackson ImmunoResearch, 705-165-147) and donkey-anti-rabbit Alexa Fluor 488 (1:1000; Molecular Probes, A21206) were used as secondary antibodies. DAPI (Sigma, D8417) was used for counterstaining. Slides were mounted with anti-fluorescence mounting medium (Dako, S3023), and images were acquired with a Leica SP5 and SP8 confocal microscope.

### Analysis of bone formation rate by Calcein-Alizarin red S labeling

Five-week-old mice were injected intraperitoneally with 20 mg/kg Calcein (1 mg/mL in 2% NaHCO_3_ solution) and 40 mg/kg Alizarin red S (2 mg/mL in H_2_O) on day 0 and day 4 separately. On day 7, the mice were euthanized, and the bones were fixed, dehydrated, and embedded with Embed-812 (Electron Microscopy Sciences). The samples were sectioned as 5 μm with hard tissue cutter. The distance between Calcein and Alizarin red S positive bands were measured every 25 microns along the cortical bone surface for the length of the tibia using Image J. Consecutive sections were stained with silver nitrate to measure trabecular bone.

### μ-QCT analysis

Preparation of skeletal tissue and μ-QCT analysis were performed as previously described [55]. The mouse femurs were skinned and fixed in 70% ethanol. Scanning was performed with the instrument μ-QCT system SkyScan1176 (Bruker, Kartuizersweg, Belgium). For analysis of femoral bone mass, a region of trabecular bone 2.0 mm wide was contoured, starting 600 microns from the proximal end of the distal femoral growth plate. For femoral trabecular bone, a threshold of 80–255 permille was used. The region of interest of the femoral cortical bone was 1.0 mm wide, starting 3.7 mm from the proximal end of the distal femoral growth plate. For femoral cortical bone, a threshold of 125–255 permille was used. Three-dimensional reconstructions were created by stacking the two-dimensional images from the indicated regions.

### Real-time RT-PCR analysis

Total RNA was prepared using TRIzol (T9424; Sigma) and was reverse transcribed into cDNA with the PrimeScript RT Reagent Kit (PR037A; TakaRa). The real-time reverse transcriptase RT-PCR reaction was performed with the BioRad CFX96 system. The primer sets used were listed in S2 Table.

### ChIP-seq and RNA-seq

A total of 10^7^ bone marrow cells from *Setd2*^*fl/fl*^ mice were infected with lentivirus expressing GFP and Cre, respectively. After 4-day puromycin treatment, GFP- and Cre-BMSCs were fixed by 1% formaldehyde for 10 minutes and terminated by glycine for 5 minutes (final concentration = 0.125 M). After 2 times washing by precooling PBS (protease inhibitor containing), cells were scraped and resuspended in SDS-lysis buffer, respectively (50 mM Tris-HCl [pH 7.5], 10 mM EDTA, 1% SDS and protease inhibitor), and sonicated. Cells were centrifuged to obtain cell extracts, which then were added to precleaning protein G agarose and rotated for 1 hour at 4 °C. Extracts were centrifuged, and supernatants were harvested to a new tube. ChIP assay was performed using H3K36me3 antibodies. Normal IgG was used as a negative control. qPCR was used to amplify various regions of the target gene genome, and primers for ChIP-qPCR primers are listed in S2 Table.

### ChIP-seq data processing

The high-throughput sequencing was performed by the Computational Biology Omics Core, CAS-MPG Partner Institute for Computer Biology (PICB), Shanghai Institutes for Biological Sciences, Chinese Academy of Sciences. The SOAP version 2.20 alignment tool was used to align ChIP-seq reads to the mouse genome build mm9 [56]. The reads with fewer than 2 mismatches that uniquely mapped to the genome were used in subsequent analyses. We calculated the distance from the peak centers to the annotated TSSs and then defined the nearest genes as peak-related genes.

### RNA-seq data processing

Raw reads were mapped to mm9 using the TopHat version 1.4.1 program [57]. We assigned fragment per kilo base per million (FPKM) as an expression value for each gene using Cufflinks version 1.3.0 software [58]. Then, Cuffdiff software was used to identify differentially expressed genes between treatment and control samples [59]. Differentially expressed gene heat maps were clustered by k-means clustering using the Euclidean distance as the distance and visualized using Java TreeView software [60] (series GSE120361).

### Antibodies

Anti-SETD2 (LS-B12660-50) was from LSBIO (Seattle, WA); anti-H3K36me1 (ab9048), anti-H3K36me2 (ab9049), and H3K36me3 (ab9050) were from Abcam (Cambridge, UK); rabbit-anti-Perilipin A/B (P1873) was from Sigma; rabbit-anti-Collagen I (600-400-103) was from Rockland (PA, USA); anti-Lbp (11836-1AP) and donkey-anti-OPN (AF808) were from R&D Systems (Minneapolis, MN); anit-Osx (sc-133871) and anti-tubulin (sc-23948) were from Santa Cruz Biotechnology (Texas, USA); donkey-anti-goat cy3 (705-165-147) was from Jackson ImmunoResearch (PA, USA); and donkey-anti-rabbit Alexa Fluor 488 (A21206) was from Molecular Probes.

### Statistics

Each experiment was performed more than twice, and the data are presented as the mean ± SD. Student *t* test was used to compare the effects of all treatments. Statistically significant differences are indicated as follows: “*” for *p* < 0.05 and “**” for *p* < 0.01.

## Supporting information

S1 Data Numerical data used in Figs 1, 2, 3, 4, 5, 6 and 7, S1, S2, S4, S5, S7, S9, S10 and S11 Figs. (XLSX)

S1 Table ChIP-seq and RNA-seq overlapped genes. (DOCX)

S2 Table Primers for RT-qPCR and ChIP-qPCR. (DOCX)

S1 Fig Characterization of *Prx1-Cre*, *Setd2*^*fl/fl*^ mice. (A) *Prx1-Cre*, *Setd2*^*fl/fl*^ mice construction strategy. (B) Southern blot of *Setd2* after PstI digestion, target allele was digested into 2 segments with length of 5k and 22k. (C) Genotyping of WT, *Setd2*^*fl/+*^ and *Setd2*^*fl/fl*^ mice. (D) qPCR analysis of *Setd2* expression in mBMSCs and liver from WT and *Prx1-Cre*, *Setd2*^*fl/fl*^ mice. Results are presented as the mean ± SD, *n =* 4 per condition. (E) Western blot analysis of SETD2 in liver. (F) Representative image by fluorescence microscopy from the femur shown prx1 positive cells using *Prx1-Cre*, *Ai9/+* mice. Scale bar = 1 mm. (G) Immunohistochemistry assay of H3K36me1/2 level in hindlimb growth plate of *Prx1-Cre*, *Setd2*^*fl/fl*^ mice and WT control mice. Data used in the generation of this figure can be found in S1 Data.
(TIF)

S2 Fig *Setd2* deficiency altered genome-wide H3K36me3 occupancy and downstream gene expression. (A) Analysis of *Setd2*, *Pparγ1*, *Pparγ2*, *Cebpα*, *Fabp4*, and *Perilipin* via qPCR of BMSCs isolated from *Setd2*^*fl/fl*^ mice treated with Cre and GFP lentivirus induced by adipogenesis medium for 6 days. Results are presented as the mean ± SD, *n =* 4 per condition. (B) Relative expression of differential genes in the control (GFP) versus *Setd2*-deficient (Cre) BMCSs. Results are presented as the mean ± SD, *n =* 4 per condition. Data used in the generation of this figure can be found in S1 Data.
(TIF)

S3 Fig H3K36me3 ChIP-seq profiles of 19+X/Y chromosomes of mouse. The black bars on top of each panel show 10-kb scale. All panels have the same signal scale of 0–5 RPM on the y-axis.
(TIF)

S4 Fig Screen of *Setd2* regulated genes. (A, B) Relative expression levels of indicated genes in WT and *Prx1-Cre*, *Setd2*^*fl/fl*^ mice. Results are presented as the mean ± SD, *n =* 4 per condition. (C) Morphological image of BMSCs at day 6 induced by adipogenesis medium, BMSCs were infected with lentivirus expressing GFP, Ptx, Lbp, and B3galt2. Cells were stained with Oil Red O. Upper panels, stained dishes, scale bar = 1 mm; lower panels, representative fields under the microscope, scale bar = 100 μm. (D) Quantitative analysis of Oil Red staining. Results are presented as the mean ± SD, *n =* 4 per condition. (E) Expression analysis of indicated genes. Results are presented as the mean ± SD, *n =* 4 per condition. (F–G) qPCR analysis of *Lbp* during adipogenesis (panel F) and osteogenesis (panel G). Data used in the generation of this figure can be found in S1 Data.
(TIF)

S5 Fig Recombinant LBP promotes osteogenesis and represses adipogenesis. (A) Alp activity and Alizarin red S staining after osteoblast differentiation for 7 days (upper) and 21 days (lower), respectively, with rLBP treatment. Scale bar = 1 mm. (B) Alp activity quantification was measured by phosphatase substrate assay. The results are represented as mean ± SD, *n =* 4 for each treatment. (C) qPCR analysis of *Osx*, *Alp*, and *Bsp* expression after osteoblast differentiation for 7 days with rLBP administration; cells were from WT mBMSCs. (D) Oil Red O staining after adipogenesis for 6 days, scale bar = 1 mm. (E) Quantitative analysis of Oil Red O staining, the results are represented as mean ± SD, *n =* 3. (F) Expression analysis of indicated genes, including *Pparγ1*, *Cebpα*, *Fabp4*, and *Perilipin*. Data used in the generation of this figure can be found in S1 Data.
(TIF)

S6 Fig ChIP-seq profiles of indicated fundamental genes. ChIP-seq profiles of indicated fundamental genes by H3K36me3 antibody shown in Integrated Genomic Viewer. The black bars on top of each panel show 5-kb scale. All panels have the same signal scale of 0–5 RPM on the y-axis. The expanded RefSeq gene is shown below each panel.
(TIF)

S7 Fig SETD2 regulates the transcription level of Lbp via altering the trimethylation level of H3K36. (A–B) Oil Red O staining and quantification of adipocytes differentiated from mBMSC which were infected with lentivirus expressing GFP and t*Setd2* followed by adipocyte differentiation for 6 days, scale bar = 1 mm. (C) H3K36me3 levels in WT cells infected with lentivirus expressing GPF and t*Setd2*. Data used in the generation of this figure can be found in S1 Data.
(TIF)

S8 Fig *Setd2* deficiency barely affected the chondrocyte differentiation and cartilage formation. (A) Safranin O staining at embryonic day 16.5 in WT and *Prx1-Cre; Setd2*^*fl/fl*^ mice, scale bar = 100 μm. (B) Safranin O staining at 5 weeks at the cartilage, scale bar = 100 μm. (C) Alcien blue staining for micromass culture at D7; chondrocyte progenitors were isolated from *Setd2*^*fl/fl*^ mice at P3 and infected with GFP and Cre-lentivirus, scale bar = 1 mm.
(TIF)

S9 Fig Expression levels of *Setd2* and Lbp in aging mouse bone marrow. (A–C) Analysis of *Pparγ1*, *Setd2*, and *Lbp* via qPCR of BMSCs isolated from 20-week and 60-week WT mice. Results are presented as the mean ± SD, *n =* 3 mice per condition. Data used in the generation of this figure can be found in S1 Data.
(TIF)

S10 Fig Analysis of bone marrow hematopoiesis cells in the mutant mice. (A) Flow cytometric analysis of Lineage-Sca-1+ c-kit+ LSK cells and CD150+ CD48− Lineage-Sca-1+ c-kit+ HSCs of bone marrow cells that are from WT and *Prx1-Cre; Setd2*^*fl/fl*^ mice. (B) Quantification of LSK cells and HSCs. Results are presented as the mean ± SD, *n =* 3 per condition. Data used in the generation of this figure can be found in S1 Data.
(TIF)

S11 Fig Proliferation was increased after loss of *Setd2* in BMSCs. Data used in the generation of this figure can be found in S1 Data.
(TIF)

## Abbreviations

μ-QCT: micro-quantitative computed tomography
ALP: alkaline phosphatase
B3galt2: β-1,3-galactosyltransferase 2
BMD: bone mineral density
BMSC: bone marrow mesenchymal stem cell
Bsp: bone sialoprotein
BV/TV: bone volume per tissue volume
CEBPα: CCAAT enhancer binding protein α
ChIP-seq: chromatin immunoprecipitation sequencing
Col1α1: Collagen type 1 alpha 1
ESC: embryonic stem cell
EZH2: enhancer of zeste homolog 2
Fabp4: fatty acid binding protein 4
FBS: fetal bovine serum
FPKM: fragment per kilo base per million
GFP: green fluorescent protein
H3K36me3: H3 lysine 36 trimethylation
HEK293T: human embryonic kidney cell line
hMSC: human mesenchymal stem cell
IgG: immunoglobulin G
Kb: kilo base pairs
KDM6A/B: Lysine-specific demthylase 6A/B
LBP: lipopolysaccharide-binding protein
MAR: mineral apposition rate
mBMSC: mouse bone marrow mesenchymal stem cell
MSC: mesenchymal stem cell
Opn: osteopontin
Osx: Sp7 transcription factor
PICB: Partner Institute for Computer Biology
Pparγ1/2: peroxisome proliferative activated receptor γ1/2
PPRE: PPARγ response element
PTX3: plasma long pentraxin 3
qPCR: quantitative PCR
RNA-seq: RNA sequencing
RT-PCR: reverse transcription PCR
Runx2: runt-related transcription factor 2
SETD2: SET-domain-containing 2
shRNA: short hairpin RNA
SPF: specific pathogen free
Tb. N: trabecular number
Tb. Sp: trabecular space
Tb. Th: trabecular thickness
TES: transcription end site
TSS: transcription start site
WT: wild-type
