# Supplementary material for: H3K36 trimethylation mediated by SETD2 regulates the fate of bone marrow mesenchymal stem cells
Source: PLoS Biol. 2018 Nov 13;16(11):e2006522. doi: 10.1371/journal.pbio.2006522 (PMC6233919; doi:10.1371/journal.pbio.2006522)
Supplement: S1 Table — (DOCX) [file pbio.2006522.s002.docx]

| **Supplemental Table 1. ChIP-seq and RNA-seq overlapped genes** | | | | |
| --- | --- | --- | --- | --- |
|  |  |  |  |  |
| Down regulated |  |  |  |  |
|  | Symbol | Fold Change | KO_Normalized | WT_Normalized |
| 1 | Igsf5 | 0 | 0 | 5.293121069 |
| 2 | Phxr4 | 0.082672447 | 0.463923617 | 5.61158686 |
| 3 | BC051019 | 0.086776351 | 0.463923617 | 5.346198701 |
| 4 | Ppil6 | 0.087518751 | 0.463247344 | 5.293121069 |
| 5 | Amd-ps7 | 0.093794414 | 0.463247344 | 4.938965147 |
| 6 | Fcrl5 | 0.142996739 | 0.926494688 | 6.4791316 |
| 7 | LOC102632324 | 0.152698704 | 0.927170961 | 6.071898046 |
| 8 | LOC102632865 | 0.172244003 | 1.390418305 | 8.072375684 |
| 9 | Gm6478 | 0.175324758 | 1.390418305 | 7.930530288 |
| 10 | Gm19410 | 0.177744543 | 3.244083955 | 18.25138427 |
| 11 | Ldhal6b | 0.185153056 | 1.389742032 | 7.505909234 |
| 12 | LOC102633868 | 0.185729771 | 0.927170961 | 4.992042779 |
| 13 | LOC102640510 | 0.187992004 | 1.391094579 | 7.399753971 |
| 14 | Siglec15 | 0.194121174 | 0.927847235 | 4.779732252 |
| 15 | LOC102638565 | 0.204744526 | 0.927847235 | 4.531731593 |
| 16 | LOC102635346 | 0.207020004 | 0.927170961 | 4.478653962 |
| 17 | LOC102633270 | 0.209350064 | 0.926494688 | 4.42557633 |
| 18 | Gm6997 | 0.212046023 | 0.927170961 | 4.372498698 |
| 19 | Clic5 | 0.212959281 | 1.391094579 | 6.532209231 |
| 20 | Gm5665 | 0.216477264 | 1.391094579 | 6.426053968 |
| 21 | Gm18180 | 0.222867554 | 0.927170961 | 4.160188171 |
| 22 | LOC102640993 | 0.223827659 | 1.854341923 | 8.284686211 |
| 23 | Gm9826 | 0.234632747 | 5.561673222 | 23.70373823 |
| 24 | Zfp433 | 0.235273695 | 1.391094579 | 5.91266515 |
| 25 | Cttnbp2 | 0.237174038 | 1.389742032 | 5.859587519 |
| 26 | LOC102640058 | 0.244036498 | 1.391094579 | 5.700354623 |
| 27 | Pm20d1 | 0.261128376 | 1.853665649 | 7.09867568 |
| 28 | Arhgef15 | 0.262939546 | 1.391770852 | 5.293121069 |
| 29 | LOC102632428 | 0.266792823 | 2.318941814 | 8.691919764 |
| 30 | Gm10125 | 0.268197168 | 2.316912993 | 8.638842133 |
| 31 | LOC102632445 | 0.272594752 | 1.389742032 | 5.098198043 |
| 32 | Got1l1 | 0.284716866 | 1.391094579 | 4.885887516 |
| 33 | LOC102638598 | 0.284855279 | 1.391770852 | 4.885887516 |
| 34 | Aqp9 | 0.286202232 | 1.854341923 | 6.4791316 |
| 35 | LOC102639045 | 0.287105073 | 4865.869625 | 16948.04472 |
| 36 | Lars2 | 0.287375751 | 23644.77067 | 82278.23869 |
| 37 | Csrnp3 | 0.287703911 | 1.390418305 | 4.832809884 |
| 38 | Avil | 0.288487341 | 33.83869686 | 117.2969905 |
| 39 | Gm18537 | 0.292963946 | 2.31826554 | 7.913142788 |
| 40 | LOC102632667 | 0.308090588 | 1.854341923 | 6.018820414 |
| 41 | Lmntd2 | 0.308588427 | 2.31623672 | 7.505909234 |
| 42 | Gm4131 | 0.310303507 | 1.389742032 | 4.478653962 |
| 43 | Mir1191 | 0.310718332 | 1.853665649 | 5.965742782 |
| 44 | C77370 | 0.310888372 | 3.709360119 | 11.93148556 |
| 45 | Asb15 | 0.314177906 | 1.390418305 | 4.42557633 |
| 46 | LOC102640781 | 0.31657829 | 1.855018196 | 5.859587519 |
| 47 | Hist1h3e | 0.318146368 | 1.391094579 | 4.372498698 |
| 48 | LOC102640226 | 0.318301033 | 1.391770852 | 4.372498698 |
| 49 | 1500002F19Rik | 0.326062494 | 1.391094579 | 4.266343435 |
| 50 | LOC102640018 | 0.326221007 | 1.391770852 | 4.266343435 |
| 51 | LOC102632656 | 0.329849123 | 1.389742032 | 4.213265803 |
| 52 | LOC102634060 | 0.330009634 | 1.390418305 | 4.213265803 |
| 53 | Setd2 | 0.331060041 | 472.3655395 | 1426.827406 |
| 54 | LOC102639975 | 0.337066121 | 1.85569447 | 5.505431596 |
| 55 | Brinp3 | 0.339632853 | 8.345214927 | 24.57128297 |
| 56 | Pcdh11x | 0.344488007 | 2.780836611 | 8.072375684 |
| 57 | Ntrk2 | 0.346725917 | 1.853665649 | 5.346198701 |
| 58 | LOC102640306 | 0.346768083 | 2.780836611 | 8.019298052 |
| 59 | 1700109H08Rik | 0.348221597 | 4.173283737 | 11.9845632 |
| 60 | Gm16853 | 0.350458297 | 1.855018196 | 5.293121069 |
| 61 | B3galt2 | 0.351934443 | 2.317589267 | 6.585286863 |
| 62 | Ccdc151 | 0.353255374 | 4.633825987 | 13.11749609 |
| 63 | LOC102631674 | 0.354271927 | 4.170578643 | 11.77225267 |
| 64 | Klk8 | 0.354897625 | 2.31826554 | 6.532209231 |
| 65 | LOC102636011 | 0.355406867 | 1.390418305 | 3.912187512 |
| 66 | LOC102634451 | 0.360470321 | 1.391094579 | 3.859109881 |
| 67 | LOC102635436 | 0.360470321 | 1.391094579 | 3.859109881 |
| 68 | Alas2 | 0.365408476 | 2.781512884 | 7.612064498 |
| 69 | BC051142 | 0.366499026 | 2.31623672 | 6.319898704 |
| 70 | Mx1 | 0.366952254 | 6.489520457 | 17.68491782 |
| 71 | LOC102634016 | 0.371324071 | 1.853665649 | 4.992042779 |
| 72 | Vstm5 | 0.372361866 | 4.172607463 | 11.20578622 |
| 73 | Rasgrf2 | 0.375314584 | 1.853665649 | 4.938965147 |
| 74 | Tldc2 | 0.378159574 | 3.246789049 | 8.585764501 |
| 75 | Mir3057 | 0.379391798 | 1.853665649 | 4.885887516 |
| 76 | LOC102635695 | 0.38355857 | 1.853665649 | 4.832809884 |
| 77 | Gm8810 | 0.383838438 | 1.855018196 | 4.832809884 |
| 78 | F2rl2 | 0.383978372 | 1.85569447 | 4.832809884 |
| 79 | LOC102633015 | 0.385057056 | 2.317589267 | 6.018820414 |
| 80 | Dcstamp | 0.385057056 | 2.317589267 | 6.018820414 |
| 81 | Ltb4r2 | 0.385746821 | 3.708007572 | 9.612542136 |
| 82 | LOC102631929 | 0.385968336 | 2.780836611 | 7.204830944 |
| 83 | Sorcs1 | 0.387312738 | 5.101130972 | 13.17057373 |
| 84 | LOC102634150 | 0.388369576 | 2.316912993 | 5.965742782 |
| 85 | LOC102636709 | 0.388596295 | 2.31826554 | 5.965742782 |
| 86 | Awat2 | 0.392199077 | 2.318941814 | 5.91266515 |
| 87 | Prr16 | 0.397832377 | 11.12740408 | 27.97008167 |
| 88 | LOC102635342 | 0.403478186 | 4.171254916 | 10.33824148 |
| 89 | Ryr3 | 0.405295729 | 3.709360119 | 9.15223095 |
| 90 | Epha7 | 0.40639498 | 12.97836464 | 31.93534681 |
| 91 | Gm8590 | 0.407569572 | 3.246789049 | 7.96622042 |
| 92 | Atp6v0a4 | 0.407617745 | 6.956149168 | 17.06537374 |
| 93 | Slc12a5 | 0.408919975 | 9.273062162 | 22.6769606 |
| 94 | Ccdc158 | 0.413762229 | 4.636531081 | 11.20578622 |
| 95 | Gm18446 | 0.41657477 | 6.489520457 | 15.57828492 |
| 96 | 4930405O22Rik | 0.419005748 | 2.781512884 | 6.638364495 |
| 97 | LOC102633288 | 0.420964138 | 2.317589267 | 5.505431596 |
| 98 | LOC102633304 | 0.421086976 | 2.31826554 | 5.505431596 |
| 99 | Gm17399 | 0.423313772 | 3.244760228 | 7.66514213 |
| 100 | Nek11 | 0.423937382 | 1.853665649 | 4.372498698 |
| 101 | Gm6147 | 0.424092047 | 1.854341923 | 4.372498698 |
| 102 | Gpr98 | 0.425186177 | 2.31826554 | 5.452353965 |
| 103 | LOC102637087 | 0.425607974 | 2.780160337 | 6.532209231 |
| 104 | LOC102640428 | 0.425815032 | 2.781512884 | 6.532209231 |
| 105 | LOC102633587 | 0.426603981 | 3.708007572 | 8.691919764 |
| 106 | Palmd | 0.427059476 | 23.17927404 | 54.27645412 |
| 107 | Fam189a1 | 0.427530476 | 19.93519008 | 46.62869949 |
| 108 | Soat2 | 0.429274881 | 15.29730645 | 35.6352238 |
| 109 | Cdhr4 | 0.432036089 | 3.709360119 | 8.585764501 |
| 110 | Nrip2 | 0.432036089 | 7.418720239 | 17.171529 |
| 111 | LOC102639983 | 0.432114856 | 3.710036393 | 8.585764501 |
| 112 | LOC102637867 | 0.433628765 | 2.31826554 | 5.346198701 |
| 113 | Lrrc19 | 0.434644316 | 1.854341923 | 4.266343435 |
| 114 | LOC102632349 | 0.434802829 | 1.855018196 | 4.266343435 |
| 115 | LOC102635490 | 0.434802829 | 3.710036393 | 8.532686869 |
| 116 | Sirpb1a | 0.435282068 | 3.244083955 | 7.452831603 |
| 117 | Kcnk10 | 0.436099186 | 9.734956959 | 22.32280468 |
| 118 | LOC102634041 | 0.436401236 | 5.562349495 | 12.74595267 |
| 119 | Cd300e | 0.436956832 | 7.418043965 | 16.97660598 |
| 120 | Cd69 | 0.437721519 | 2.316912993 | 5.293121069 |
| 121 | Slfn5os | 0.438104813 | 2.318941814 | 5.293121069 |
| 122 | LOC102638890 | 0.438587082 | 3.245436502 | 7.399753971 |
| 123 | LOC102636131 | 0.439626157 | 7.416691418 | 16.87045071 |
| 124 | F5 | 0.439782402 | 41.25403574 | 93.8055628 |
| 125 | 9330136K24Rik | 0.439959342 | 1.853665649 | 4.213265803 |
| 126 | LOC102640483 | 0.440119852 | 1.854341923 | 4.213265803 |
| 127 | Lgr5 | 0.44077029 | 9.269680794 | 21.03063888 |
| 128 | Tas1r3 | 0.441825253 | 7.883996403 | 17.84415071 |
| 129 | 9130208D14Rik | 0.444925809 | 6.954796621 | 15.63136255 |
| 130 | Slc9b2 | 0.450920575 | 6.952767801 | 15.41905202 |
| 131 | Gm10614 | 0.453209271 | 4.17193119 | 9.205308582 |
| 132 | Gm15401 | 0.457093742 | 3.244760228 | 7.09867568 |
| 133 | Gm19006 | 0.458096111 | 2.781512884 | 6.071898046 |
| 134 | Muc6 | 0.459506484 | 2.31826554 | 5.045120411 |
| 135 | LOC101056014 | 0.45970457 | 3094.377291 | 6731.230217 |
| 136 | LOC102640818 | 0.462023523 | 2.780836611 | 6.018820414 |
| 137 | LOC102633533 | 0.462248242 | 2.782189158 | 6.018820414 |
| 138 | Prlr | 0.463833628 | 4.171254916 | 8.992998055 |
| 139 | Mir3101 | 0.464033 | 3.244760228 | 6.992520417 |
| 140 | LOC102631891 | 0.464392162 | 2.31826554 | 4.992042779 |
| 141 | Rpsa-ps9 | 0.464931955 | 6.954796621 | 14.95874084 |
| 142 | Agt | 0.465052532 | 6.026273113 | 12.9582632 |
| 143 | Kcnk12 | 0.466020819 | 2.780160337 | 5.965742782 |
| 144 | Gm16712 | 0.467064788 | 7.416691418 | 15.87936321 |
| 145 | LOC102638784 | 0.467619794 | 11.58929888 | 24.7835935 |
| 146 | LOC102637522 | 0.468588483 | 3.708007572 | 7.913142788 |
| 147 | Fras1 | 0.468839547 | 18.54139041 | 39.54741131 |
| 148 | 4931408D14Rik | 0.470433013 | 2.781512884 | 5.91266515 |
| 149 | Jpx | 0.472726397 | 5.565054589 | 11.77225267 |
| 150 | Ovol1 | 0.474205144 | 2.316912993 | 4.885887516 |
| 151 | Mir8091 | 0.474343558 | 2.317589267 | 4.885887516 |
| 152 | Lphn3 | 0.474343558 | 4.635178534 | 9.771775031 |
| 153 | LOC102633302 | 0.474481971 | 2.31826554 | 4.885887516 |
| 154 | 1700047I17Rik2 | 0.478155313 | 39.37737679 | 82.35269105 |
| 155 | Hs3st1 | 0.478235274 | 22.24872171 | 46.52254423 |
| 156 | Tuba8 | 0.479273296 | 2.31623672 | 4.832809884 |
| 157 | LOC102640426 | 0.48072781 | 9.735633232 | 20.25186191 |
| 158 | D4Ertd617e | 0.481766952 | 6.49019673 | 13.47165202 |
| 159 | Gm18787 | 0.487122458 | 3.708007572 | 7.612064498 |
| 160 | Tm6sf2 | 0.488585356 | 3.243407681 | 6.638364495 |
| 161 | LOC102633203 | 0.491962816 | 5.565054589 | 11.31194148 |
| 162 | Dlx6os1 | 0.492405916 | 7.418043965 | 15.0648961 |
| 163 | Pdzrn4 | 0.494624524 | 8.344538653 | 16.87045071 |
| 164 | Zdhhc23 | 0.495891963 | 2.317589267 | 4.673576989 |
| 165 | Fam209 | 0.501007373 | 1.853665649 | 3.699876985 |
| 166 | Pgam1-ps1 | 0.501007373 | 1.853665649 | 3.699876985 |
| 167 | Enpep | 0.501075783 | 5.100454698 | 10.17900859 |
| 168 | BC021891 | 0.501190156 | 1.854341923 | 3.699876985 |
| 169 | 6430550D23Rik | 0.503459739 | 4.63450226 | 9.205308582 |
| 170 | Mamdc4 | 0.507085035 | 12.98039346 | 25.59806061 |
| 171 | 4933406M09Rik | 0.508299325 | 1.853665649 | 3.646799354 |
| 172 | LOC102632247 | 0.508484768 | 1.854341923 | 3.646799354 |
| 173 | Cadps2 | 0.508484768 | 1.854341923 | 3.646799354 |
| 174 | Erdr1 | 0.508984444 | 6.487491636 | 12.74595267 |
| 175 | LOC102640518 | 0.509143618 | 3.244760228 | 6.372976336 |
| 176 | Cntd1 | 0.510148993 | 2.781512884 | 5.452353965 |
| 177 | Sorbs2os | 0.511300321 | 11.12402272 | 21.75633823 |
| 178 | Fbxw25 | 0.513316437 | 5.097749604 | 9.931007926 |
| 179 | A830082N09Rik | 0.514655736 | 3.708007572 | 7.204830944 |
| 180 | Gm2762 | 0.515806674 | 1.853665649 | 3.593721722 |
| 181 | 1700125H03Rik | 0.517776509 | 2.318941814 | 4.478653962 |
| 182 | Stag3 | 0.51985038 | 14.83473538 | 28.53654812 |
| 183 | Cd160 | 0.520152124 | 2.780836611 | 5.346198701 |
| 184 | Gm7434 | 0.520152124 | 2.780836611 | 5.346198701 |
| 185 | LOC101055727 | 0.52027862 | 2.781512884 | 5.346198701 |
| 186 | LOC102638100 | 0.521750231 | 5.098425878 | 9.771775031 |
| 187 | Gm15950 | 0.522161484 | 3.706655026 | 7.09867568 |
| 188 | Grem1 | 0.522489489 | 484.4194383 | 927.1371931 |
| 189 | Gm4787 | 0.522542554 | 3.709360119 | 7.09867568 |
| 190 | Grpr | 0.523731379 | 8.807785997 | 16.81737308 |
| 191 | LOC102633993 | 0.523986401 | 2.318941814 | 4.42557633 |
| 192 | Oip5 | 0.524704091 | 20.39776115 | 38.8747896 |
| 193 | Arl9 | 0.52524027 | 2.780160337 | 5.293121069 |
| 194 | Rtkn2 | 0.526172282 | 16.6917824 | 31.72303629 |
| 195 | Zbp1 | 0.526427416 | 48.21694764 | 91.59277463 |
| 196 | LOC102640247 | 0.527215785 | 22.71670297 | 43.0880554 |
| 197 | Gm4117 | 0.530572122 | 3.710036393 | 6.992520417 |
| 198 | Npas3 | 0.530642592 | 9.271709615 | 17.47260729 |
| 199 | LOC102632921 | 0.530668835 | 3.710712666 | 6.992520417 |
| 200 | Mmp16 | 0.534038212 | 94.57617199 | 177.0962637 |
| 201 | Mia | 0.534501152 | 3.245436502 | 6.071898046 |
| 202 | 1700102P08Rik | 0.534723907 | 3.246789049 | 6.071898046 |
| 203 | Tet1 | 0.534986472 | 25.03091087 | 46.78793239 |
| 204 | Ifit1 | 0.536452193 | 206.7503329 | 385.40309 |
| 205 | Rgcc | 0.536644216 | 28.74703372 | 53.56814228 |
| 206 | Bsn | 0.537312018 | 19.00463775 | 35.36983564 |
| 207 | Tnfsf11 | 0.538132119 | 69.06578321 | 128.3435438 |
| 208 | Kcnq5 | 0.538225913 | 24.10576873 | 44.78745475 |
| 209 | Slc13a3 | 0.539709495 | 46.82517679 | 86.75996475 |
| 210 | Adamts9 | 0.539963029 | 125.1619933 | 231.7973389 |
| 211 | Cmpk2 | 0.541844674 | 32.91017336 | 60.73728309 |
| 212 | Gm15446 | 0.542508641 | 11.13078545 | 20.51725007 |
| 213 | Kng1 | 0.542864611 | 6.025596839 | 11.09963096 |
| 214 | Scube2 | 0.542909111 | 2.31623672 | 4.266343435 |
| 215 | LOC102633913 | 0.543226138 | 2.317589267 | 4.266343435 |
| 216 | 3000002C10Rik | 0.543305395 | 4.635854807 | 8.532686869 |
| 217 | Gm9992 | 0.543384651 | 2.31826554 | 4.266343435 |
| 218 | Fbxw18 | 0.543618317 | 5.562349495 | 10.23208622 |
| 219 | Cfap70 | 0.543816598 | 5.564378316 | 10.23208622 |
| 220 | 4930594M22Rik | 0.5441255 | 3.246112775 | 5.965742782 |
| 221 | Rspo3 | 0.54511227 | 32.91558354 | 60.38312717 |
| 222 | Sfxn4 | 0.548275765 | 17.61624827 | 32.13026984 |
| 223 | LOC102636463 | 0.548895704 | 3.245436502 | 5.91266515 |
| 224 | Acsl6 | 0.549745458 | 16.22312487 | 29.51024812 |
| 225 | Dlg2 | 0.551895427 | 6.956149168 | 12.60410728 |
| 226 | Slfn4 | 0.553636915 | 3.244083955 | 5.859587519 |
| 227 | Agmo | 0.553752328 | 3.244760228 | 5.859587519 |
| 228 | Scn8a | 0.553838572 | 1.852989376 | 3.345721063 |
| 229 | LOC102633778 | 0.554242834 | 1.854341923 | 3.345721063 |
| 230 | Kcnc3 | 0.55565924 | 41.25471201 | 74.24462524 |
| 231 | Agtr1a | 0.556583124 | 6.956149168 | 12.49795201 |
| 232 | Mgst2 | 0.556622325 | 11.58794633 | 20.81832836 |
| 233 | LOC102640673 | 0.556788599 | 24.10915009 | 43.30036593 |
| 234 | 1810019D21Rik | 0.557641017 | 11.12537526 | 19.95078362 |
| 235 | Spire2 | 0.559721993 | 18.08287698 | 32.30689023 |
| 236 | Cass4 | 0.559778003 | 4.17193119 | 7.452831603 |
| 237 | Nr2f1 | 0.559977154 | 23.64252138 | 42.22051066 |
| 238 | Iqcd | 0.560393036 | 5.099102151 | 9.099153318 |
| 239 | Kcnn3 | 0.560658096 | 22.25210308 | 39.68925671 |
| 240 | Grin3b | 0.560788142 | 12.05186995 | 21.49095007 |
| 241 | Snord89 | 0.563177265 | 2.781512884 | 4.938965147 |
| 242 | Zcchc16 | 0.563314191 | 2.782189158 | 4.938965147 |
| 243 | Zswim5 | 0.564204571 | 3.246112775 | 5.753432255 |
| 244 | Sdcbp2 | 0.564542492 | 16.68975358 | 29.56332575 |
| 245 | Zfp960 | 0.565164421 | 12.05592759 | 21.33171717 |
| 246 | Fam169b | 0.565414666 | 10.19955685 | 18.03907374 |
| 247 | Prkag3 | 0.565776661 | 8.343186106 | 14.74643031 |
| 248 | Onecut2 | 0.566418739 | 17.61760082 | 31.1034922 |
| 249 | Dgki | 0.566426264 | 5.565054589 | 9.824852663 |
| 250 | Zfp354b | 0.567388175 | 12.5151173 | 22.05741652 |
| 251 | Malat1 | 0.567734672 | 9418.078869 | 16588.87388 |
| 252 | LOC102638353 | 0.567767946 | 12.05119368 | 21.22556191 |
| 253 | Cyp2d22 | 0.568689543 | 22.24872171 | 39.12279026 |
| 254 | Gm5345 | 0.568953654 | 20.85897968 | 36.66200143 |
| 255 | Nespas | 0.56901849 | 2.780160337 | 4.885887516 |
| 256 | Rufy4 | 0.569156904 | 2.780836611 | 4.885887516 |
| 257 | Gm1720 | 0.569156904 | 2.780836611 | 4.885887516 |
| 258 | Gpr85 | 0.56932822 | 20.3991137 | 35.83014682 |
| 259 | Adora3 | 0.569433731 | 2.782189158 | 4.885887516 |
| 260 | Rab11fip4 | 0.569433731 | 2.782189158 | 4.885887516 |
| 261 | Trp53tg5 | 0.569433731 | 2.782189158 | 4.885887516 |
| 262 | Cnr1 | 0.570505972 | 28.27837619 | 49.567187 |
| 263 | Esr1 | 0.572261326 | 24.57645508 | 42.94621001 |
| 264 | Tmem254c | 0.572865441 | 12.97092563 | 22.6421856 |
| 265 | Flrt3 | 0.572880112 | 23.18333168 | 40.46803368 |
| 266 | 1700094D03Rik | 0.573727447 | 19.46991392 | 33.93582445 |
| 267 | Pecam1 | 0.573887928 | 16.22447742 | 28.27115996 |
| 268 | Gm19327 | 0.575267888 | 2.780160337 | 4.832809884 |
| 269 | LOC102635282 | 0.575547756 | 2.781512884 | 4.832809884 |
| 270 | Ptx3 | 0.575812362 | 501.5954325 | 871.1091771 |
| 271 | 1700034H15Rik | 0.575967558 | 2.783541705 | 4.832809884 |
| 272 | F830016B08Rik | 0.576997076 | 25.95470046 | 44.98237777 |
| 273 | D5Ertd605e | 0.577079764 | 7.416691418 | 12.85210794 |
| 274 | H2-K2 | 0.579234096 | 4.173283737 | 7.204830944 |
| 275 | Gm16340 | 0.579933409 | 6.026273113 | 10.39131911 |
| 276 | Gm18720 | 0.581599488 | 4.633149714 | 7.96622042 |
| 277 | Pkdrej | 0.581939058 | 4.635854807 | 7.96622042 |
| 278 | Rnu7 | 0.582080546 | 2.782189158 | 4.779732252 |
| 279 | Il34 | 0.582638078 | 89.93964091 | 154.3662255 |
| 280 | Ccdc125 | 0.582828161 | 34.30600185 | 58.86126335 |
| 281 | Gnrh1 | 0.585078027 | 7.881967583 | 13.47165202 |
| 282 | Shisa2 | 0.585385189 | 13.90688815 | 23.75681587 |
| 283 | Egf | 0.586345849 | 9.736309506 | 16.60506255 |
| 284 | Pcdh18 | 0.587263772 | 314.7620304 | 535.9806708 |
| 285 | Map7d2 | 0.587316726 | 15.76190635 | 26.83714877 |
| 286 | Gm4951 | 0.588260876 | 35.22979144 | 59.88804098 |
| 287 | Kbtbd8 | 0.589412384 | 17.1536772 | 29.10301457 |
| 288 | Acer2 | 0.589829139 | 17.61489572 | 29.86440404 |
| 289 | Kctd14 | 0.591580844 | 1.853665649 | 3.133410536 |
| 290 | LOC102633081 | 0.591941041 | 4.170578643 | 7.045598049 |
| 291 | St8sia6 | 0.592920989 | 2.319618087 | 3.912187512 |
| 292 | Zkscan2 | 0.59337843 | 7.416015145 | 12.49795201 |
| 293 | Ppp4r4 | 0.593396747 | 6.954120348 | 11.71917504 |
| 294 | Cmss1 | 0.593838922 | 25.49280566 | 42.9288225 |
| 295 | Maneal | 0.594138236 | 5.101130972 | 8.585764501 |
| 296 | Vcan | 0.594272408 | 6011.582424 | 10115.87 |
| 297 | Mest | 0.594917723 | 50.06723192 | 84.15824566 |
| 298 | Lsm7 | 0.595980606 | 6.952767801 | 11.66609741 |
| 299 | Zfp473 | 0.596579158 | 8.807785997 | 14.76381781 |
| 300 | Gm10557 | 0.596857591 | 39.40037009 | 66.01301666 |
| 301 | Qrfp | 0.596917815 | 4.17396001 | 6.992520417 |
| 302 | Lbp | 0.597675563 | 5.099778425 | 8.532686869 |
| 303 | 4930599N23Rik | 0.598334227 | 6.492901824 | 10.8516303 |
| 304 | Miat | 0.600711555 | 3.243407681 | 5.399276333 |
| 305 | Snora43 | 0.600836808 | 3.244083955 | 5.399276333 |
| 306 | LOC102635047 | 0.600899434 | 6.488844183 | 10.79855267 |
| 307 | LOC101056104 | 0.600936482 | 5.563702042 | 9.258386214 |
| 308 | Trpm3 | 0.601051662 | 10.66145165 | 17.73799545 |
| 309 | Ifit3 | 0.60246734 | 124.6980697 | 206.9789704 |
| 310 | Plscr2 | 0.603687531 | 299.9469069 | 496.8578805 |
| 311 | Gm11110 | 0.603742808 | 14.83473538 | 24.57128297 |
| 312 | Fmo1 | 0.604328005 | 5.563025769 | 9.205308582 |
| 313 | Afap1l1 | 0.60440147 | 5.563702042 | 9.205308582 |
| 314 | Fbln7 | 0.605136682 | 9.737662053 | 16.09167374 |
| 315 | Sarnp | 0.605219759 | 76.0266663 | 125.618282 |
| 316 | Gm12734 | 0.605413523 | 12.51782239 | 20.67648296 |
| 317 | Phf11d | 0.60590075 | 46.81909032 | 77.27188051 |
| 318 | Phf11b | 0.605972165 | 8.3458912 | 13.77273031 |
| 319 | LOC102635305 | 0.606496141 | 10.66145165 | 17.57876256 |
| 320 | 4930404I05Rik | 0.607054972 | 3.245436502 | 5.346198701 |
| 321 | Gm6297 | 0.607054972 | 3.245436502 | 5.346198701 |
| 322 | Shank1 | 0.607277368 | 12.05119368 | 19.84462835 |
| 323 | Bend5 | 0.607452665 | 8.807109724 | 14.49842965 |
| 324 | Tmem200a | 0.60908282 | 83.90795761 | 137.7611629 |
| 325 | Pla2g5 | 0.609102968 | 4.636531081 | 7.612064498 |
| 326 | Mir7652 | 0.609162196 | 6.955472895 | 11.41809675 |
| 327 | LOC101055823 | 0.609458338 | 2.319618087 | 3.806032249 |
| 328 | LOC102637103 | 0.609883648 | 10.66686183 | 17.48999479 |
| 329 | Gm19178 | 0.61057206 | 3.707331299 | 6.071898046 |
| 330 | B3galt1 | 0.611033059 | 23.18062658 | 37.93677973 |
| 331 | LOC101669761 | 0.611670606 | 7.417367692 | 12.12640859 |
| 332 | Pcdhb21 | 0.611965471 | 15.29663018 | 24.99590403 |
| 333 | LOC102633307 | 0.612341084 | 11.58794633 | 18.92400598 |
| 334 | Ddx60 | 0.612405419 | 73.70907703 | 120.3599359 |
| 335 | Rps18-ps3 | 0.61301455 | 3.244760228 | 5.293121069 |
| 336 | Ntn1 | 0.613062944 | 389.4199191 | 635.2038125 |
| 337 | Tbxa2r | 0.613129504 | 11.12537526 | 18.14522901 |
| 338 | Ckap2 | 0.613343448 | 285.558512 | 465.5768528 |
| 339 | 4933408B17Rik | 0.613379963 | 4.636531081 | 7.558986866 |
| 340 | Pax3 | 0.615956457 | 3.707331299 | 6.018820414 |
| 341 | 4930480K23Rik | 0.616181177 | 3.708683846 | 6.018820414 |
| 342 | Sv2a | 0.616181177 | 3.708683846 | 6.018820414 |
| 343 | Gm18874 | 0.617468417 | 41.72269327 | 67.57057061 |
| 344 | LOC102635447 | 0.617717446 | 4.636531081 | 7.505909234 |
| 345 | LOC102638022 | 0.618365546 | 4.170578643 | 6.744519758 |
| 346 | Iigp1 | 0.618374962 | 84.82701328 | 137.177309 |
| 347 | Mreg | 0.618480457 | 16.22582996 | 26.23499219 |
| 348 | Gm6182 | 0.619094859 | 3.244083955 | 5.240043438 |
| 349 | Nr3c2 | 0.619861288 | 6.024244292 | 9.718697399 |
| 350 | Cabp1 | 0.619954482 | 9.273738435 | 14.95874084 |
| 351 | AW011738 | 0.620280106 | 12.97904091 | 20.92448362 |
| 352 | Ifitm5 | 0.621211012 | 2.782189158 | 4.478653962 |
| 353 | Galnt13 | 0.621328966 | 69.99836436 | 112.6591036 |
| 354 | Mir8108 | 0.621469403 | 6.490873004 | 10.44439674 |
| 355 | LOC102632235 | 0.621663384 | 3.708683846 | 5.965742782 |
| 356 | Gpc3 | 0.62181437 | 8.34386238 | 13.41857438 |
| 357 | Wasf3 | 0.622537962 | 27.81580512 | 44.68129948 |
| 358 | Osbpl6 | 0.622758187 | 17.61692454 | 28.28854746 |
| 359 | 5031414D18Rik | 0.623012548 | 24.57239744 | 39.44125605 |
| 360 | Neat1 | 0.623046429 | 2101.46105 | 3372.880338 |
| 361 | Aspm | 0.623184695 | 204.4225995 | 328.0289155 |
| 362 | Sytl5 | 0.623510439 | 152.971712 | 245.339456 |
| 363 | Gm20199 | 0.623573721 | 4.172607463 | 6.691442127 |
| 364 | Cenpf | 0.624042161 | 293.4343932 | 470.2156548 |
| 365 | Gm527 | 0.624112263 | 9.269680794 | 14.85258557 |
| 366 | Dapk2 | 0.624555099 | 7.418720239 | 11.87840793 |
| 367 | Adamtsl3 | 0.624627355 | 266.0879218 | 425.9946665 |
| 368 | Gm13248 | 0.625198279 | 4.172607463 | 6.674054626 |
| 369 | Gm14288 | 0.625299607 | 3.243407681 | 5.186965806 |
| 370 | Ptger4 | 0.625651448 | 78.81426564 | 125.9715228 |
| 371 | Zbtb26 | 0.625693677 | 99.19985387 | 158.5438012 |
| 372 | LOC102634026 | 0.626105544 | 7.880615036 | 12.58671978 |
| 373 | LOC102640300 | 0.626213521 | 2.316912993 | 3.699876985 |
| 374 | Lst1 | 0.626271938 | 42.18458806 | 67.35826008 |
| 375 | Syt9 | 0.626396304 | 2.317589267 | 3.699876985 |
| 376 | Gm10389 | 0.626396304 | 2.317589267 | 3.699876985 |
| 377 | Penk | 0.626579086 | 4.636531081 | 7.399753971 |
| 378 | AK157302 | 0.626670477 | 4.637207354 | 7.399753971 |
| 379 | Stxbp6 | 0.626970867 | 37.54805698 | 59.88804098 |
| 380 | Hsd17b1 | 0.627015264 | 3.707331299 | 5.91266515 |
| 381 | Nudt15 | 0.627058438 | 6.02762566 | 9.612542136 |
| 382 | Gsto2 | 0.627274745 | 8.805757177 | 14.03811847 |
| 383 | Galnt14 | 0.627358395 | 3.709360119 | 5.91266515 |
| 384 | LOC102635191 | 0.627358395 | 3.709360119 | 5.91266515 |
| 385 | LOC102632226 | 0.627358395 | 3.709360119 | 5.91266515 |
| 386 | Tmem74b | 0.627563789 | 8.809814818 | 14.03811847 |
| 387 | Ism2 | 0.628127936 | 10.66348047 | 16.97660598 |
| 388 | LOC102637030 | 0.628388662 | 27.81039493 | 44.25667843 |
| 389 | LOC102637810 | 0.628476981 | 12.04916486 | 19.17200664 |
| 390 | Zfp953 | 0.630880313 | 31.98570749 | 50.7001199 |
| 391 | Mir145a | 0.63109087 | 6.490873004 | 10.28516385 |
| 392 | 5730480H06Rik | 0.631673048 | 10.1982043 | 16.14475137 |
| 393 | Map7 | 0.632642628 | 18.54612432 | 29.31532509 |
| 394 | Fam169a | 0.632925754 | 3.708683846 | 5.859587519 |
| 395 | Ms4a4a | 0.63321454 | 11.59065143 | 18.3044619 |
| 396 | Zfp783 | 0.633444213 | 33.38288853 | 52.70059754 |
| 397 | Papolb | 0.633625771 | 4.172607463 | 6.585286863 |
| 398 | LOC102635872 | 0.63383116 | 4.17396001 | 6.585286863 |
| 399 | Ern2 | 0.634364573 | 6.490873004 | 10.23208622 |
| 400 | 4933413G19Rik | 0.634812575 | 8.810491091 | 13.87888557 |
| 401 | Prrg3 | 0.635018966 | 11.58997516 | 18.25138427 |
| 402 | N4bp2l1 | 0.635257815 | 19.00531403 | 29.91748167 |
| 403 | Slc35g1 | 0.635276865 | 60.26746504 | 94.86803056 |
| 404 | Kctd16 | 0.635698681 | 2.31826554 | 3.646799354 |
| 405 | Fbn2 | 0.635700984 | 75.10625807 | 118.1471477 |
| 406 | LOC102632568 | 0.635769595 | 5.098425878 | 8.019298052 |
| 407 | Ano7 | 0.635983405 | 2.780836611 | 4.372498698 |
| 408 | LOC102640372 | 0.636190241 | 13.44702217 | 21.13679415 |
| 409 | LOC102633214 | 0.636292736 | 2.782189158 | 4.372498698 |
| 410 | Lck | 0.636447401 | 2.782865431 | 4.372498698 |
| 411 | H2-Q6 | 0.637118448 | 88.54854633 | 138.9828636 |
| 412 | 4930565N06Rik | 0.637237061 | 15.75920125 | 24.73051587 |
| 413 | Mylk3 | 0.63753954 | 6.489520457 | 10.17900859 |
| 414 | Sybu | 0.637605978 | 6.49019673 | 10.17900859 |
| 415 | Serp2 | 0.638510404 | 11.58591751 | 18.14522901 |
| 416 | Dock3 | 0.638594895 | 3.708007572 | 5.806509887 |
| 417 | LOC101055753 | 0.638611596 | 12.05119368 | 18.87092835 |
| 418 | Klf5 | 0.639114272 | 18.54341923 | 29.0142468 |
| 419 | Dlk2 | 0.639400118 | 46.36260572 | 72.50953576 |
| 420 | LOC102640216 | 0.639497507 | 9.271709615 | 14.49842965 |
| 421 | LOC102639643 | 0.639867069 | 5.561673222 | 8.691919764 |
| 422 | Has1 | 0.64052486 | 6.95073898 | 10.8516303 |
| 423 | Depdc1a | 0.641366949 | 24.56969234 | 38.30832315 |
| 424 | Ttn | 0.64172436 | 9.735633232 | 15.17105136 |
| 425 | Drp2 | 0.641728202 | 28.27567109 | 44.0617554 |
| 426 | Elovl4 | 0.642396144 | 38.47184658 | 59.88804098 |
| 427 | Lphn2 | 0.642434562 | 452.9192952 | 705.0045588 |
| 428 | Tgtp1 | 0.642716341 | 47.75234775 | 74.29770287 |
| 429 | Zfp72 | 0.643962884 | 28.73891844 | 44.62822185 |
| 430 | Tfcp2l1 | 0.644392686 | 6.490873004 | 10.07285332 |
| 431 | Eda | 0.645069431 | 78.3483132 | 121.4571787 |
| 432 | Pkia | 0.645221628 | 199.79486 | 309.6530734 |
| 433 | Samd10 | 0.646578493 | 31.52246014 | 48.75271989 |
| 434 | Gm5595 | 0.646788025 | 34.77330683 | 53.7630653 |
| 435 | Il18rap | 0.646900766 | 73.71516349 | 113.9512694 |
| 436 | LOC102634849 | 0.647043329 | 6.024920566 | 9.311463845 |
| 437 | LOC102636279 | 0.647477241 | 14.37351686 | 22.19926191 |
| 438 | Nrcam | 0.649219182 | 81.58766324 | 125.6704445 |
| 439 | Cml1 | 0.649326551 | 8.345214927 | 12.85210794 |
| 440 | Slc24a3 | 0.64952668 | 132.5868 | 204.1283355 |
| 441 | Cxadr | 0.649543182 | 106.6260131 | 164.155388 |
| 442 | Mansc1 | 0.650279799 | 17.15232465 | 26.37683758 |
| 443 | Angpt2 | 0.650853731 | 33.83937314 | 51.99228569 |
| 444 | 6330549D23Rik | 0.651588308 | 10.65807028 | 16.35706189 |
| 445 | Fgf9 | 0.651649446 | 2.780160337 | 4.266343435 |
| 446 | Olfml2a | 0.651966473 | 2.781512884 | 4.266343435 |
| 447 | Snora34 | 0.651966473 | 2.781512884 | 4.266343435 |
| 448 | Tctex1d4 | 0.652124987 | 2.782189158 | 4.266343435 |
| 449 | Gm15191 | 0.652124987 | 2.782189158 | 4.266343435 |
| 450 | Gm17970 | 0.652124987 | 2.782189158 | 4.266343435 |
| 451 | Ppef2 | 0.652442014 | 2.783541705 | 4.266343435 |
| 452 | Slfn5 | 0.652947866 | 483.5071454 | 740.4988524 |
| 453 | Masp2 | 0.653059108 | 4.635854807 | 7.09867568 |
| 454 | Gm13139 | 0.653108944 | 62.12113069 | 95.11603122 |
| 455 | LOC102634533 | 0.653154376 | 4.636531081 | 7.09867568 |
| 456 | Malt1 | 0.653342189 | 82.97334764 | 126.9983004 |
| 457 | Zfp423 | 0.653346445 | 27.35323405 | 41.86635474 |
| 458 | Cep55 | 0.653470067 | 158.0782532 | 241.9058823 |
| 459 | Itgb7 | 0.653535452 | 29.20081324 | 44.68129948 |
| 460 | 2610307P16Rik | 0.654729125 | 10.19955685 | 15.57828492 |
| 461 | Adam32 | 0.654734498 | 4.172607463 | 6.372976336 |
| 462 | Trim30d | 0.654869694 | 143.7054126 | 219.4412323 |
| 463 | Sema3c | 0.655003017 | 407.4798028 | 622.103704 |
| 464 | Zfpm2 | 0.655086978 | 221.1143819 | 337.5343873 |
| 465 | Zgrf1 | 0.655681832 | 78.80141645 | 120.1824004 |
| 466 | Fam217b | 0.655736027 | 45.8986821 | 69.9956693 |
| 467 | Traf1 | 0.655955811 | 19.93789518 | 30.39518036 |
| 468 | Pde1a | 0.656337214 | 8.807109724 | 13.41857438 |
| 469 | Cdkl5 | 0.656426018 | 17.15164838 | 26.12883693 |
| 470 | Sema6a | 0.656813864 | 18.54544805 | 28.23546983 |
| 471 | Vpreb1 | 0.65697168 | 3.244760228 | 4.938965147 |
| 472 | Tgfbr3 | 0.657029658 | 339.3608025 | 516.5075859 |
| 473 | Gm5820 | 0.657108606 | 3.245436502 | 4.938965147 |
| 474 | LOC102639767 | 0.657108606 | 3.245436502 | 4.938965147 |
| 475 | Svep1 | 0.657841589 | 1448.23225 | 2201.490866 |
| 476 | Ccdc64b | 0.657978893 | 4.635854807 | 7.045598049 |
| 477 | Slc5a3 | 0.658455101 | 203.5076015 | 309.0683043 |
| 478 | 4933431G14Rik | 0.659912261 | 4.170578643 | 6.319898704 |
| 479 | Ska1 | 0.659988713 | 24.57036862 | 37.22846788 |
| 480 | Gm5904 | 0.660019268 | 2.780836611 | 4.213265803 |
| 481 | Syt1 | 0.660019268 | 2.780836611 | 4.213265803 |
| 482 | 4930481A15Rik | 0.660432326 | 7.879938762 | 11.93148556 |
| 483 | Slc4a4 | 0.660464032 | 196.5528048 | 297.598045 |
| 484 | Ptger3 | 0.661672547 | 8.808462271 | 13.31241912 |
| 485 | Akap5 | 0.662043706 | 7.418720239 | 11.20578622 |
| 486 | LOC102640610 | 0.662049161 | 8.344538653 | 12.60410728 |
| 487 | Treml1 | 0.662289435 | 6.026273113 | 9.099153318 |
| 488 | Bmpr1b | 0.662508029 | 34.3046493 | 51.77997516 |
| 489 | Spats1 | 0.662512403 | 6.028301933 | 9.099153318 |
| 490 | LOC102640017 | 0.66277994 | 4.63450226 | 6.992520417 |
| 491 | Abca13 | 0.662876654 | 4.635178534 | 6.992520417 |
| 492 | B3gat2 | 0.663021724 | 9.272385888 | 13.98504083 |
| 493 | Frmd5 | 0.663070081 | 9.273062162 | 13.98504083 |
| 494 | Tns4 | 0.663594782 | 11.12469899 | 16.76429545 |
| 495 | Slc7a2 | 0.664230525 | 284.1701226 | 427.8185237 |
| 496 | LOC102638508 | 0.664385491 | 3.246112775 | 4.885887516 |
| 497 | LOC102639688 | 0.664385491 | 3.246112775 | 4.885887516 |
| 498 | Ccser1 | 0.664821542 | 10.66280419 | 16.0385961 |
| 499 | Gm20257 | 0.665353523 | 9.269680794 | 13.9319632 |
| 500 | LOC102635552 | 0.66619276 | 20.86236104 | 31.31580273 |
| 501 | Tmprss7 | 0.66632492 | 6.02762566 | 9.046075687 |
| 502 | Gm7162 | 0.666532552 | 7.881967583 | 11.8253303 |
| Up regulated |  |  |  |  |
| 1 | Zfp85os | 1.501583812 | 4.173283737 | 2.779254614 |
| 2 | Nup210 | 1.501760398 | 22.2514268 | 14.81689544 |
| 3 | Jag2 | 1.502083582 | 10.66280419 | 7.09867568 |
| 4 | 1700084E18Rik | 1.502092019 | 3.244083955 | 2.159710533 |
| 5 | Gm11359 | 1.502092019 | 3.244083955 | 2.159710533 |
| 6 | Mgat5b | 1.502092019 | 3.244083955 | 2.159710533 |
| 7 | Gpr135 | 1.502474962 | 23.64522648 | 15.73751781 |
| 8 | Fgf22 | 1.502874848 | 6.491549277 | 4.319421066 |
| 9 | Ldb2 | 1.503027126 | 12.0532225 | 8.019298052 |
| 10 | Cerkl | 1.503031413 | 3.246112775 | 2.159710533 |
| 11 | Rnu73b | 1.503031413 | 3.246112775 | 2.159710533 |
| 12 | LOC102632284 | 1.503387685 | 5.562349495 | 3.699876985 |
| 13 | Sh2d5 | 1.503487198 | 8.809814818 | 5.859587519 |
| 14 | Kcnk13 | 1.507440784 | 93.6395332 | 62.11821664 |
| 15 | Rab3b | 1.510240953 | 76.48923737 | 50.64704227 |
| 16 | E2f2 | 1.511168358 | 42.18729316 | 27.91700404 |
| 17 | Gm2810 | 1.512428148 | 26.42606309 | 17.47260729 |
| 18 | Spef1 | 1.516868171 | 124.246319 | 81.90976737 |
| 19 | Nr4a2 | 1.517090897 | 116.3690854 | 76.70541406 |
| 20 | LOC102635108 | 1.517844019 | 7.416015145 | 4.885887516 |
| 21 | Proser2 | 1.518331724 | 46.82247169 | 30.83810405 |
| 22 | 2610027K06Rik | 1.518397674 | 7.418720239 | 4.885887516 |
| 23 | Cspg4 | 1.518816631 | 140.0007864 | 92.17754371 |
| 24 | Gpr137c | 1.519838239 | 34.30397303 | 22.57080533 |
| 25 | Dok7 | 1.520621051 | 6.487491636 | 4.266343435 |
| 26 | Hpx | 1.521572132 | 25.96619711 | 17.06537374 |
| 27 | Frmd8 | 1.521804922 | 2481.184559 | 1630.42222 |
| 28 | Gfra2 | 1.522616646 | 71.86014529 | 47.19516594 |
| 29 | Myh11 | 1.522631798 | 1126.506632 | 739.8417879 |
| 30 | Il27 | 1.52526886 | 5.562349495 | 3.646799354 |
| 31 | Susd2 | 1.525333481 | 41.25944592 | 27.0494593 |
| 32 | 4930538K18Rik | 1.525454304 | 5.563025769 | 3.646799354 |
| 33 | Hook1 | 1.525454304 | 5.563025769 | 3.646799354 |
| 34 | 2700069I18Rik | 1.527643085 | 9.735633232 | 6.372976336 |
| 35 | Bmp2 | 1.528594638 | 13.90891697 | 9.099153318 |
| 36 | Sema3d | 1.529586451 | 69.53511702 | 45.46007646 |
| 37 | E030024N20Rik | 1.530074879 | 8.342509833 | 5.452353965 |
| 38 | Snora78 | 1.530074879 | 4.171254916 | 2.726176982 |
| 39 | Gm9574 | 1.530322946 | 4.17193119 | 2.726176982 |
| 40 | Cacna1e | 1.530571012 | 4.172607463 | 2.726176982 |
| 41 | Ppp1r14c | 1.530819079 | 4.173283737 | 2.726176982 |
| 42 | Tmem156 | 1.530936821 | 12.98174601 | 8.479609237 |
| 43 | Rbm20 | 1.533002575 | 11.58794633 | 7.558986866 |
| 44 | Tpd52-ps | 1.539117042 | 32.45030738 | 21.08371651 |
| 45 | LOC102639843 | 1.539937952 | 3.244083955 | 2.106632901 |
| 46 | Cyyr1 | 1.540258973 | 6.489520457 | 4.213265803 |
| 47 | LOC102632435 | 1.540328427 | 21.78682691 | 14.14427373 |
| 48 | Dmpk | 1.540380101 | 7507.527798 | 4873.8151 |
| 49 | Il18bp | 1.540813701 | 209.5372559 | 135.9912985 |
| 50 | Gstm6 | 1.540901015 | 3.246112775 | 2.106632901 |
| 51 | Fam19a1 | 1.541222036 | 3.246789049 | 2.106632901 |
| 52 | Dlec1 | 1.54384312 | 11.58794633 | 7.505909234 |
| 53 | Wdr17 | 1.544113418 | 11.58997516 | 7.505909234 |
| 54 | Cul9 | 1.545311515 | 259.6078692 | 167.9971104 |
| 55 | Wfikkn1 | 1.546604766 | 13.44296453 | 8.691919764 |
| 56 | Gm19025 | 1.547008377 | 19.0059903 | 12.28564149 |
| 57 | LOC100503002 | 1.547391278 | 23.63981629 | 15.27720663 |
| 58 | LOC102632600 | 1.548224006 | 5.097749604 | 3.292643432 |
| 59 | Ttll9 | 1.548840174 | 5.099778425 | 3.292643432 |
| 60 | Col19a1 | 1.549045563 | 5.100454698 | 3.292643432 |
| 61 | Cpsf4l | 1.549045563 | 5.100454698 | 3.292643432 |
| 62 | Fbxw15 | 1.549250952 | 5.101130972 | 3.292643432 |
| 63 | Mycbpap | 1.549966352 | 30.12730792 | 19.4373948 |
| 64 | Fuz | 1.55008636 | 60.72597847 | 39.17586789 |
| 65 | Mroh8 | 1.550279807 | 12.51444102 | 8.072375684 |
| 66 | Ramp1 | 1.551443342 | 91.31991511 | 58.86126335 |
| 67 | Mkrn1-ps1 | 1.551696 | 7.416691418 | 4.779732252 |
| 68 | Cd300lf | 1.556768163 | 46.82247169 | 30.07671457 |
| 69 | Ctsw | 1.556932272 | 15.29663018 | 9.824852663 |
| 70 | C1qtnf5 | 1.558965265 | 262.8675075 | 168.6166545 |
| 71 | Gm4356 | 1.56020338 | 4.170578643 | 2.673099351 |
| 72 | Pvrig-ps | 1.560456372 | 4.171254916 | 2.673099351 |
| 73 | Gpat2 | 1.560456373 | 8.342509833 | 5.346198701 |
| 74 | LOC102640449 | 1.560709365 | 4.17193119 | 2.673099351 |
| 75 | Gm10420 | 1.560709365 | 4.17193119 | 2.673099351 |
| 76 | Klkb1 | 1.561215349 | 4.173283737 | 2.673099351 |
| 77 | LOC102633880 | 1.561303707 | 24.57104489 | 15.73751781 |
| 78 | Nek10 | 1.561425314 | 10.19955685 | 6.532209231 |
| 79 | Snapc5 | 1.561883671 | 216.4947576 | 138.6113202 |
| 80 | Gm12799 | 1.569406813 | 7.418043965 | 4.72665462 |
| 81 | LOC102635673 | 1.569692966 | 7.419396512 | 4.72665462 |
| 82 | Fabp7 | 1.570896178 | 67.68686156 | 43.0880554 |
| 83 | LOC102632388 | 1.571500772 | 6.954796621 | 4.42557633 |
| 84 | Thoc6 | 1.571784552 | 292.9927866 | 186.4077276 |
| 85 | Bean1 | 1.573223448 | 22.25210308 | 14.14427373 |
| 86 | LOC102636194 | 1.573381634 | 5.097073331 | 3.2395658 |
| 87 | Ak8 | 1.574216651 | 10.19955685 | 6.4791316 |
| 88 | LOC102635559 | 1.574216651 | 5.099778425 | 3.2395658 |
| 89 | Snora41 | 1.574425405 | 5.100454698 | 3.2395658 |
| 90 | Gm7525 | 1.574425405 | 5.100454698 | 3.2395658 |
| 91 | Gm9079 | 1.5759423 | 13.44702217 | 8.532686869 |
| 92 | Rps15a-ps4 | 1.576156941 | 12.974307 | 8.231608579 |
| 93 | Rgs3 | 1.576407072 | 593.4029408 | 376.4274795 |
| 94 | Nrg2 | 1.576615161 | 8.345214927 | 5.293121069 |
| 95 | Ankrd55 | 1.582861279 | 15.29933527 | 9.665619768 |
| 96 | 9430060I03Rik | 1.582931247 | 15.30001155 | 9.665619768 |
| 97 | Gm19325 | 1.583703255 | 6.02762566 | 3.806032249 |
| 98 | Kcp | 1.587535013 | 10.20158567 | 6.426053968 |
| 99 | Pdzd9 | 1.591460668 | 15.29798273 | 9.612542136 |
| 100 | Gm3219 | 1.594644756 | 12.05389877 | 7.558986866 |
| 101 | Hs3st3a1 | 1.598318005 | 43.57297755 | 27.26176982 |
| 102 | LOC102633886 | 1.60033241 | 10.19888058 | 6.372976336 |
| 103 | LOC102636299 | 1.60033241 | 10.19888058 | 6.372976336 |
| 104 | Hist1h3i | 1.600650757 | 5.100454698 | 3.186488168 |
| 105 | LOC102636956 | 1.605741004 | 6.026273113 | 3.752954617 |
| 106 | Wnt11 | 1.605835495 | 156.2367604 | 97.29312926 |
| 107 | Fam198a | 1.606101399 | 6.02762566 | 3.752954617 |
| 108 | Ddah2 | 1.606143316 | 800.1870874 | 498.2040391 |
| 109 | Npas1 | 1.612795779 | 7.879938762 | 4.885887516 |
| 110 | LOC102638993 | 1.612934193 | 7.880615036 | 4.885887516 |
| 111 | Trim15 | 1.613072606 | 7.881291309 | 4.885887516 |
| 112 | Hsf4 | 1.613095582 | 84.83986248 | 52.59444227 |
| 113 | Gm11839 | 1.613349433 | 7.882643856 | 4.885887516 |
| 114 | Eci3 | 1.615409794 | 8.807785997 | 5.452353965 |
| 115 | Kank3 | 1.615940014 | 83.90254742 | 51.92182056 |
| 116 | Hoxc5 | 1.616296416 | 158.542853 | 98.09020886 |
| 117 | 4931402G19Rik | 1.619391374 | 10.66415674 | 6.585286863 |
| 118 | Mill2 | 1.622926253 | 37.5500858 | 23.13727178 |
| 119 | Anapc15-ps | 1.624988615 | 4.171254916 | 2.566944087 |
| 120 | Ccdc170 | 1.62525207 | 4.17193119 | 2.566944087 |
| 121 | Rtn4r | 1.626438086 | 41.71998817 | 25.65113824 |
| 122 | 4921513I03Rik | 1.627332931 | 5.099102151 | 3.133410536 |
| 123 | Syt14 | 1.628959398 | 6.026949386 | 3.699876985 |
| 124 | Acaa1b | 1.629202873 | 6.95073898 | 4.266343435 |
| 125 | Spocd1 | 1.629678414 | 6.952767801 | 4.266343435 |
| 126 | Apoe | 1.629679257 | 2620.628096 | 1608.063725 |
| 127 | Hoxa4 | 1.631415346 | 17.61692454 | 10.79855267 |
| 128 | Synpo2l | 1.631928605 | 10.6600991 | 6.532209231 |
| 129 | Slc40a1 | 1.632470068 | 667.0748228 | 408.6291296 |
| 130 | Trem1 | 1.63254978 | 10.66415674 | 6.532209231 |
| 131 | Pdf | 1.632962108 | 253.5775385 | 155.2868479 |
| 132 | Ndufaf3 | 1.634591934 | 146.5051848 | 89.62798713 |
| 133 | Dync1i1 | 1.63519752 | 30.597318 | 18.71169545 |
| 134 | Dact2 | 1.635497416 | 35.23520163 | 21.5440277 |
| 135 | Adam5 | 1.636736914 | 26.42471054 | 16.14475137 |
| 136 | Tmem117 | 1.640207422 | 14.83744048 | 9.046075687 |
| 137 | Eno1b | 1.644348773 | 82.49589855 | 50.16934358 |
| 138 | Nrip3 | 1.644995227 | 11.58997516 | 7.045598049 |
| 139 | A530013C23Rik | 1.64571502 | 10.66280419 | 6.4791316 |
| 140 | Dsp | 1.646172416 | 19.46653255 | 11.8253303 |
| 141 | Exoc3l | 1.647821267 | 43.11446412 | 26.16452706 |
| 142 | D630029K05Rik | 1.65285366 | 6.02762566 | 3.646799354 |
| 143 | LOC102638255 | 1.654274108 | 32.91625982 | 19.89770598 |
| 144 | Mfsd2b | 1.657191633 | 11.58794633 | 6.992520417 |
| 145 | Lgals4 | 1.659308223 | 10.66280419 | 6.426053968 |
| 146 | Axdnd1 | 1.661026157 | 9.732928138 | 5.859587519 |
| 147 | 1810010D01Rik | 1.661256983 | 9.734280685 | 5.859587519 |
| 148 | 4732491K20Rik | 1.663462074 | 19.93586636 | 11.9845632 |
| 149 | Zfand4 | 1.665685176 | 34.76519155 | 20.87140599 |
| 150 | Nkain4 | 1.666841952 | 7.878586215 | 4.72665462 |
| 151 | Sag | 1.667534251 | 4.63450226 | 2.779254614 |
| 152 | Ppm1j | 1.667736576 | 29.67082331 | 17.79107308 |
| 153 | Pkd1l2 | 1.668020909 | 4.635854807 | 2.779254614 |
| 154 | Scml4 | 1.671838878 | 8.3458912 | 4.992042779 |
| 155 | Them6 | 1.672386605 | 70.93500315 | 42.41543369 |
| 156 | Oit3 | 1.673360456 | 26.42335799 | 15.79059544 |
| 157 | Rpl36-ps2 | 1.674031542 | 14.37284059 | 8.585764501 |
| 158 | Gm12191 | 1.674652628 | 20.39640861 | 12.17948622 |
| 159 | Ros1 | 1.675411753 | 3.707331299 | 2.212788165 |
| 160 | Snord66 | 1.675717374 | 3.708007572 | 2.212788165 |
| 161 | 2310033P09Rik | 1.675991666 | 149.7418298 | 89.34521147 |
| 162 | Slc5a11 | 1.676328615 | 3.709360119 | 2.212788165 |
| 163 | Npy | 1.679978662 | 44.04501645 | 26.21760469 |
| 164 | Fbxo41 | 1.680019876 | 10.2009094 | 6.071898046 |
| 165 | Cd4 | 1.686939166 | 116.8248937 | 69.25258246 |
| 166 | Lrrd1 | 1.689257943 | 8.343186106 | 4.938965147 |
| 167 | Plxdc1 | 1.69042667 | 34.77263056 | 20.5703277 |
| 168 | Cd200 | 1.691123974 | 297.157955 | 175.7162453 |
| 169 | Gm3435 | 1.695024759 | 17.61354317 | 10.39131911 |
| 170 | Cfap126 | 1.696268227 | 19.00801912 | 11.20578622 |
| 171 | Ube2ql1 | 1.698048948 | 12.05389877 | 7.09867568 |
| 172 | LOC102634280 | 1.699349134 | 22.71264533 | 13.36549675 |
| 173 | Gm15559 | 1.699436482 | 18.08084816 | 10.63931977 |
| 174 | LOC102632189 | 1.700248577 | 4.635178534 | 2.726176982 |
| 175 | LOC102635286 | 1.70074471 | 4.636531081 | 2.726176982 |
| 176 | Rps12-ps9 | 1.700992777 | 4.637207354 | 2.726176982 |
| 177 | Snord72 | 1.701240843 | 4.637883628 | 2.726176982 |
| 178 | Ccdc148 | 1.703229636 | 17.15638229 | 10.07285332 |
| 179 | Smagp | 1.703858026 | 17.61489572 | 10.33824148 |
| 180 | LOC100503496 | 1.704884183 | 6.488844183 | 3.806032249 |
| 181 | Gdf9 | 1.705239553 | 6.49019673 | 3.806032249 |
| 182 | Fbxl22 | 1.705529226 | 82.51551048 | 48.38117647 |
| 183 | Cdh24 | 1.71166988 | 116.3555599 | 67.97780416 |
| 184 | Mmp12 | 1.713818243 | 2380.031628 | 1388.730478 |
| 185 | Oscar | 1.716430561 | 7.413986324 | 4.319421066 |
| 186 | LOC102637406 | 1.716587127 | 3.707331299 | 2.159710533 |
| 187 | LOC102637946 | 1.716900259 | 11.12402272 | 6.4791316 |
| 188 | Gm21451 | 1.716962885 | 9.270357068 | 5.399276333 |
| 189 | Veph1 | 1.717143805 | 16.68840103 | 9.718697399 |
| 190 | LOC102633931 | 1.71721339 | 5.563025769 | 3.2395658 |
| 191 | LOC102632739 | 1.71721339 | 7.417367692 | 4.319421066 |
| 192 | Lrp2bp | 1.717526521 | 3.709360119 | 2.159710533 |
| 193 | Khk | 1.726849911 | 58.87704674 | 34.09505735 |
| 194 | Ccdc73 | 1.729536769 | 6.490873004 | 3.752954617 |
| 195 | Gm15784 | 1.734262067 | 4.635854807 | 2.673099351 |
| 196 | Jmjd8 | 1.735205406 | 179.8819869 | 103.6661056 |
| 197 | Scnn1a | 1.737992874 | 34.30532558 | 19.73847309 |
| 198 | Ttc16 | 1.738577263 | 7.417367692 | 4.266343435 |
| 199 | Rilp | 1.73915442 | 40.33159869 | 23.19034942 |
| 200 | Mir3060 | 1.739211317 | 7.420072786 | 4.266343435 |
| 201 | Tnfsf13os | 1.740430409 | 10.1982043 | 5.859587519 |
| 202 | LOC102632695 | 1.740661235 | 10.19955685 | 5.859587519 |
| 203 | LOC102633979 | 1.745307818 | 13.90350678 | 7.96622042 |
| 204 | Gm2011 | 1.745604942 | 5.562349495 | 3.186488168 |
| 205 | Gm14373 | 1.745604942 | 5.562349495 | 3.186488168 |
| 206 | Tnni3 | 1.745675686 | 8.34386238 | 4.779732252 |
| 207 | Tstd1 | 1.745817174 | 5.563025769 | 3.186488168 |
| 208 | Cyp4f41-ps | 1.746453869 | 5.565054589 | 3.186488168 |
| 209 | Lpar2 | 1.752832103 | 45.42731947 | 25.9165264 |
| 210 | Tppp3 | 1.753555231 | 48.67478479 | 27.75777114 |
| 211 | Dner | 1.754131809 | 13.44566963 | 7.66514213 |
| 212 | Liph | 1.757386587 | 14.37284059 | 8.178530947 |
| 213 | Pygm | 1.759837367 | 7.414662598 | 4.213265803 |
| 214 | Zfp459 | 1.759837368 | 3.707331299 | 2.106632901 |
| 215 | LOC102635973 | 1.760158388 | 3.708007572 | 2.106632901 |
| 216 | LOC102633775 | 1.761442473 | 3.710712666 | 2.106632901 |
| 217 | Gramd2 | 1.762253973 | 19.93451381 | 11.31194148 |
| 218 | Gm16062 | 1.76693094 | 12.98106973 | 7.346676339 |
| 219 | Col17a1 | 1.769911799 | 4.637207354 | 2.620021719 |
| 220 | Igfbp2 | 1.773949471 | 391.7273643 | 220.8221658 |
| 221 | Gm10561 | 1.780421069 | 41.73013228 | 23.43835007 |
| 222 | Csmd1 | 1.781003647 | 7.881967583 | 4.42557633 |
| 223 | Gm12892 | 1.781469504 | 15.29527763 | 8.585764501 |
| 224 | Ccdc85b | 1.78747869 | 1282.265267 | 717.3597503 |
| 225 | Iqck | 1.795539802 | 19.00801912 | 10.58624214 |
| 226 | Sh3d21 | 1.795983998 | 25.49821585 | 14.19735136 |
| 227 | Dnah3 | 1.802468532 | 7.881291309 | 4.372498698 |
| 228 | Guca1b | 1.803380841 | 11.58862261 | 6.426053968 |
| 229 | Postn | 1.804097392 | 37815.07478 | 20960.66152 |
| 230 | Rac3 | 1.804306476 | 193.3066921 | 107.1362846 |
| 231 | Sox6 | 1.80487531 | 16.23124015 | 8.992998055 |
| 232 | Grid2ip | 1.805652678 | 3.708007572 | 2.05355527 |
| 233 | Pbld2 | 1.81582818 | 27.35526287 | 15.0648961 |
| 234 | Bmf | 1.815943189 | 26.42471054 | 14.55150728 |
| 235 | Fbxo36 | 1.818325586 | 44.96812977 | 24.73051587 |
| 236 | Spock2 | 1.823957531 | 24.57172116 | 13.47165202 |
| 237 | Fbxw10 | 1.824865482 | 30.59258409 | 16.76429545 |
| 238 | Baiap3 | 1.82713122 | 20.86236104 | 11.41809675 |
| 239 | Rasgrp2 | 1.829580974 | 38.47725677 | 21.03063888 |
| 240 | 1700112J16Rik | 1.830018028 | 6.025596839 | 3.292643432 |
| 241 | Gm16287 | 1.830018028 | 12.05119368 | 6.585286863 |
| 242 | Gm3320 | 1.835431323 | 5.101130972 | 2.779254614 |
| 243 | Tmprss11f | 1.841805345 | 4.173283737 | 2.265865797 |
| 244 | Calml4 | 1.8500482 | 14.83608793 | 8.019298052 |
| 245 | Kcnmb4 | 1.85282961 | 24.10779755 | 13.01134083 |
| 246 | Trim43c | 1.853332535 | 6.955472895 | 3.752954617 |
| 247 | Rab19 | 1.860210128 | 6.026273113 | 3.2395658 |
| 248 | Gm5466 | 1.860210128 | 6.026273113 | 3.2395658 |
| 249 | Ccdc38 | 1.860210128 | 6.026273113 | 3.2395658 |
| 250 | Rpl23a | 1.860367632 | 30.59528918 | 16.44582966 |
| 251 | Tmem108 | 1.860418883 | 6.026949386 | 3.2395658 |
| 252 | Oplah | 1.869321965 | 139.5483594 | 74.65185879 |
| 253 | Capn3 | 1.870174208 | 5.098425878 | 2.726176982 |
| 254 | Kcnk4 | 1.870670341 | 5.099778425 | 2.726176982 |
| 255 | Pnma2 | 1.870670341 | 5.099778425 | 2.726176982 |
| 256 | Scube1 | 1.87315054 | 24.57104489 | 13.11749609 |
| 257 | Immp2l | 1.873537189 | 19.93316126 | 10.63931977 |
| 258 | 9930014A18Rik | 1.875047807 | 448.27262 | 239.072635 |
| 259 | LOC102634056 | 1.879371693 | 6.953444074 | 3.699876985 |
| 260 | 5033403F01Rik | 1.879554476 | 13.9082407 | 7.399753971 |
| 261 | Flywch2 | 1.879737259 | 6.954796621 | 3.699876985 |
| 262 | LOC102636752 | 1.882354149 | 26.42471054 | 14.03811847 |
| 263 | Gm7061 | 1.884761817 | 4.170578643 | 2.212788165 |
| 264 | LOC102636155 | 1.885067438 | 4.171254916 | 2.212788165 |
| 265 | Themis | 1.885373058 | 4.17193119 | 2.212788165 |
| 266 | Ncf2-rs | 1.885678679 | 8.345214927 | 4.42557633 |
| 267 | Gm5823 | 1.892429984 | 25.49415821 | 13.47165202 |
| 268 | Slc13a5 | 1.89254382 | 332.8503175 | 175.8745631 |
| 269 | P2rx3 | 1.893133719 | 13.9082407 | 7.346676339 |
| 270 | Tmem223 | 1.89460762 | 279.0818408 | 147.3032399 |
| 271 | AA986860 | 1.89840516 | 31.52313642 | 16.60506255 |
| 272 | F13a1 | 1.902446777 | 55.16498153 | 28.9968593 |
| 273 | Reep2 | 1.902588131 | 17.61489572 | 9.258386214 |
| 274 | Vsig10l | 1.903458693 | 151.5988768 | 79.64390157 |
| 275 | Lhfpl4 | 1.906802801 | 5.097073331 | 2.673099351 |
| 276 | Slc16a8 | 1.907055794 | 5.097749604 | 2.673099351 |
| 277 | LOC102636984 | 1.907561778 | 5.099102151 | 2.673099351 |
| 278 | Klhl41 | 1.907561778 | 5.099102151 | 2.673099351 |
| 279 | Ccnb2-ps | 1.907814771 | 5.099778425 | 2.673099351 |
| 280 | Gm5628 | 1.908067763 | 5.100454698 | 2.673099351 |
| 281 | B3galt4 | 1.908140677 | 65.83522473 | 34.5022909 |
| 282 | Cd79b | 1.908723541 | 8.3458912 | 4.372498698 |
| 283 | Gipc2 | 1.924094573 | 6.028978207 | 3.133410536 |
| 284 | Slamf6 | 1.931082235 | 4.170578643 | 2.159710533 |
| 285 | Gm6263 | 1.931395367 | 8.342509833 | 4.319421066 |
| 286 | Lama3 | 1.931708498 | 4.17193119 | 2.159710533 |
| 287 | Gm12070 | 1.931708498 | 4.17193119 | 2.159710533 |
| 288 | Gm12279 | 1.931708498 | 4.17193119 | 2.159710533 |
| 289 | Fcho1 | 1.937279579 | 13.4436408 | 6.939442785 |
| 290 | Card11 | 1.937377034 | 13.44431708 | 6.939442785 |
| 291 | 4933430I17Rik | 1.942528036 | 11.58862261 | 5.965742782 |
| 292 | D230017M19Rik | 1.946722297 | 5.100454698 | 2.620021719 |
| 293 | C230062I16Rik | 1.949959389 | 9.734280685 | 4.992042779 |
| 294 | Stab2 | 1.956465236 | 14.37351686 | 7.346676339 |
| 295 | Snord55 | 1.956590269 | 6.026949386 | 3.080332905 |
| 296 | LOC102639290 | 1.970093481 | 3.243407681 | 1.646321716 |
| 297 | Gm16209 | 1.97050426 | 3.244083955 | 1.646321716 |
| 298 | LOC102637845 | 1.97050426 | 3.244083955 | 1.646321716 |
| 299 | Aldh1a3 | 1.972244714 | 28.28040501 | 14.33919676 |
| 300 | 4930415F15Rik | 1.976227313 | 7.416691418 | 3.752954617 |
| 301 | 1700003F12Rik | 1.978412339 | 11.59268025 | 5.859587519 |
| 302 | Atp7b | 1.980057804 | 4.171254916 | 2.106632901 |
| 303 | Gm7229 | 1.980378825 | 4.17193119 | 2.106632901 |
| 304 | Cmya5 | 1.984392627 | 42.64648286 | 21.49095007 |
| 305 | Tmem91 | 1.986185014 | 5.098425878 | 2.566944087 |
| 306 | C8g | 1.987016952 | 18.08017189 | 9.099153318 |
| 307 | Klhl35 | 1.994591123 | 10.66348047 | 5.346198701 |
| 308 | Tmem40 | 1.997799373 | 12.51985121 | 6.266821073 |
| 309 | LOC102640347 | 2.001138433 | 5.561673222 | 2.779254614 |
| 310 | Gm5082 | 2.001381761 | 5.562349495 | 2.779254614 |
| 311 | Fsd2 | 2.003624376 | 6.490873004 | 3.2395658 |
| 312 | 1700028K03Rik | 2.004760624 | 7.417367692 | 3.699876985 |
| 313 | LOC102632916 | 2.01434718 | 9.734956959 | 4.832809884 |
| 314 | Lamb3 | 2.014592208 | 21.32696093 | 10.58624214 |
| 315 | Sarm1 | 2.026183081 | 18.5440955 | 9.15223095 |
| 316 | Mirlet7c-2 | 2.036150008 | 3.244083955 | 1.593244084 |
| 317 | Gm6579 | 2.036150008 | 3.244083955 | 1.593244084 |
| 318 | Rbfox1 | 2.036574472 | 6.489520457 | 3.186488168 |
| 319 | Celsr1 | 2.037423398 | 3.246112775 | 1.593244084 |
| 320 | Gna14 | 2.037847861 | 3.246789049 | 1.593244084 |
| 321 | Plcd4 | 2.039603706 | 5.560320675 | 2.726176982 |
| 322 | LOC102633267 | 2.054975654 | 14.36945922 | 6.992520417 |
| 323 | Gm14446 | 2.056893287 | 12.05254623 | 5.859587519 |
| 324 | LOC102638848 | 2.071072521 | 6.489520457 | 3.133410536 |
| 325 | Nme3 | 2.072832163 | 55.62890514 | 26.83714877 |
| 326 | Itga2 | 2.080608497 | 5.561673222 | 2.673099351 |
| 327 | Pnldc1 | 2.081367473 | 5.563702042 | 2.673099351 |
| 328 | Otud7a | 2.088913351 | 19.00734285 | 9.099153318 |
| 329 | Colec10 | 2.08900524 | 61.64706297 | 29.51024812 |
| 330 | Dscam | 2.095028743 | 4.635854807 | 2.212788165 |
| 331 | Cd27 | 2.095028743 | 4.635854807 | 2.212788165 |
| 332 | Gm5481 | 2.095334363 | 4.636531081 | 2.212788165 |
| 333 | Lrrc48 | 2.105312041 | 11.59065143 | 5.505431596 |
| 334 | Fcrl1 | 2.109688259 | 13.44499335 | 6.372976336 |
| 335 | Plch2 | 2.119996239 | 9.269680794 | 4.372498698 |
| 336 | LOC101055995 | 2.1206149 | 9.272385888 | 4.372498698 |
| 337 | Riiad1 | 2.122758442 | 5.561673222 | 2.620021719 |
| 338 | Tcrb | 2.124482337 | 17.14961956 | 8.072375684 |
| 339 | Gm21284 | 2.127910451 | 6.026949386 | 2.832332246 |
| 340 | Gm20139 | 2.137156858 | 14.83067774 | 6.939442785 |
| 341 | Rhof | 2.139747652 | 37.08345709 | 17.3307619 |
| 342 | Prrt2 | 2.146382539 | 16.22447742 | 7.558986866 |
| 343 | LOC102632778 | 2.146829869 | 11.5913277 | 5.399276333 |
| 344 | Ibsp | 2.157928967 | 44.04569272 | 20.4110948 |
| 345 | Slc17a7 | 2.161945724 | 8.343186106 | 3.859109881 |
| 346 | Eef1a2 | 2.162120965 | 8.34386238 | 3.859109881 |
| 347 | Usp11 | 2.164625105 | 136.3029229 | 62.96837388 |
| 348 | Vmn2r29 | 2.168062188 | 6.025596839 | 2.779254614 |
| 349 | Fam131a | 2.171584733 | 76.03951549 | 35.01567972 |
| 350 | LOC102638354 | 2.181951862 | 3.708007572 | 1.699399348 |
| 351 | 4933417D19Rik | 2.182377584 | 6.954120348 | 3.186488168 |
| 352 | Grap | 2.189603637 | 23.6445502 | 10.79855267 |
| 353 | Mtl5 | 2.193161592 | 8.347243747 | 3.806032249 |
| 354 | Myl12b | 2.201406246 | 1176.170128 | 534.2812714 |
| 355 | Slc22a13b-ps | 2.201562325 | 4.637883628 | 2.106632901 |
| 356 | Atp8b3 | 2.21076967 | 6.026949386 | 2.726176982 |
| 357 | LOC102638847 | 2.210893703 | 12.05457505 | 5.452353965 |
| 358 | Naaladl1 | 2.217577728 | 7.419396512 | 3.345721063 |
| 359 | Fut11 | 2.24052598 | 300.4088017 | 134.0795886 |
| 360 | Amn | 2.252298282 | 3.708007572 | 1.646321716 |
| 361 | BC085271 | 2.252298282 | 3.708007572 | 1.646321716 |
| 362 | Elmo3 | 2.263514844 | 52.85212617 | 23.34958231 |
| 363 | Asb5 | 2.273353751 | 12.51579357 | 5.505431596 |
| 364 | Wdr27 | 2.289409099 | 7.416691418 | 3.2395658 |
| 365 | Adhfe1 | 2.295856518 | 12.51782239 | 5.452353965 |
| 366 | Isyna1 | 2.299867834 | 401.0281537 | 174.3700867 |
| 367 | Tgm1 | 2.32326026 | 15.29933527 | 6.585286863 |
| 368 | Fxyd2 | 2.326907306 | 3.707331299 | 1.593244084 |
| 369 | Gpr123 | 2.328074579 | 14.8367642 | 6.372976336 |
| 370 | A230056P14Rik | 2.347254215 | 8.809138544 | 3.752954617 |
| 371 | Gm15464 | 2.347644868 | 6.026273113 | 2.566944087 |
| 372 | Cpa4 | 2.371856203 | 11.58862261 | 4.885887516 |
| 373 | Nipal4 | 2.376077155 | 43.11446412 | 18.14522901 |
| 374 | Epor | 2.390226709 | 10.19752803 | 4.266343435 |
| 375 | Gm5786 | 2.408763924 | 25.03023459 | 10.39131911 |
| 376 | 4933439K11Rik | 2.408854178 | 3.710036393 | 1.540166452 |
| 377 | Susd4 | 2.41483904 | 8.80643345 | 3.646799354 |
| 378 | Stap2 | 2.420498678 | 5.099102151 | 2.106632901 |
| 379 | Pdlim3 | 2.428725872 | 6.492225551 | 2.673099351 |
| 380 | Flt1 | 2.432823346 | 7.881291309 | 3.2395658 |
| 381 | Derl3 | 2.438761268 | 10.66348047 | 4.372498698 |
| 382 | AI427809 | 2.440708669 | 12.05457505 | 4.938965147 |
| 383 | Gm13611 | 2.45190305 | 11.58929888 | 4.72665462 |
| 384 | LOC102631633 | 2.472922649 | 7.879938762 | 3.186488168 |
| 385 | LOC102633925 | 2.47715379 | 6.49019673 | 2.620021719 |
| 386 | LOC102635496 | 2.504029174 | 149.7391247 | 59.79927322 |
| 387 | Zfp114 | 2.532859969 | 4.16990237 | 1.646321716 |
| 388 | LOC102632124 | 2.534913861 | 4.173283737 | 1.646321716 |
| 389 | Snord33 | 2.54186751 | 9.269680794 | 3.646799354 |
| 390 | Slco4a1 | 2.550620931 | 20.86033222 | 8.178530947 |
| 391 | Clec2h | 2.575506953 | 5.562349495 | 2.159710533 |
| 392 | Gm11960 | 2.576028839 | 8.345214927 | 3.2395658 |
| 393 | B3gnt4 | 2.576289783 | 11.12808036 | 4.319421066 |
| 394 | Cend1 | 2.593584364 | 9.733604412 | 3.752954617 |
| 395 | Tshr | 2.594124956 | 9.735633232 | 3.752954617 |
| 396 | Cmtm8 | 2.640077072 | 5.561673222 | 2.106632901 |
| 397 | D330050G23Rik | 2.647488712 | 12.51376475 | 4.72665462 |
| 398 | Rps19-ps4 | 2.65370617 | 6.952767801 | 2.620021719 |
| 399 | Rgs4 | 2.65763703 | 1084.338951 | 408.0086704 |
| 400 | Tnfrsf25 | 2.661618129 | 14.37081177 | 5.399276333 |
| 401 | Mir3061 | 2.707875267 | 4.170578643 | 1.540166452 |
| 402 | Rnf152 | 2.70875345 | 4.17193119 | 1.540166452 |
| 403 | 1700028I16Rik | 2.709192541 | 4.172607463 | 1.540166452 |
| 404 | Gm9292 | 2.709192541 | 4.172607463 | 1.540166452 |
| 405 | LOC102637468 | 2.727539315 | 4.635178534 | 1.699399348 |
| 406 | Srp54b | 2.739582444 | 78.80750291 | 28.76624614 |
| 407 | Rltpr | 2.757216965 | 12.05592759 | 4.372498698 |
| 408 | A330074K22Rik | 2.763679947 | 8.80643345 | 3.186488168 |
| 409 | Sptbn5 | 2.774819308 | 7.417367692 | 2.673099351 |
| 410 | Robo3 | 2.790315193 | 6.026273113 | 2.159710533 |
| 411 | Gm20337 | 2.790628324 | 6.026949386 | 2.159710533 |
| 412 | Fndc5 | 2.790628324 | 6.026949386 | 2.159710533 |
| 413 | Pcdh20 | 2.801518286 | 53.3133447 | 19.03016124 |
| 414 | LOC102633356 | 2.815064768 | 4.63450226 | 1.646321716 |
| 415 | LOC102635338 | 2.815475547 | 4.635178534 | 1.646321716 |
| 416 | Pbx4 | 2.862389319 | 12.51579357 | 4.372498698 |
| 417 | Shisa6 | 2.910119754 | 4.636531081 | 1.593244084 |
| 418 | Kif17 | 3.007585283 | 7.879938762 | 2.620021719 |
| 419 | Slamf8 | 3.008955815 | 12.51782239 | 4.160188171 |
| 420 | LOC101055939 | 3.009530904 | 4.635178534 | 1.540166452 |
| 421 | Sh2d4b | 3.055710634 | 9.736985779 | 3.186488168 |
| 422 | LOC102634004 | 3.069247303 | 7.878586215 | 2.566944087 |
| 423 | Abcg4 | 3.080517946 | 6.489520457 | 2.106632901 |
| 424 | Hist1h4a | 3.088226835 | 11.58997516 | 3.752954617 |
| 425 | Fgf18 | 3.121924714 | 8.345214927 | 2.673099351 |
| 426 | Ccin | 3.142390304 | 6.953444074 | 2.212788165 |
| 427 | 2010005H15Rik | 3.143001545 | 6.954796621 | 2.212788165 |
| 428 | BC021767 | 3.185686368 | 8.346567474 | 2.620021719 |
| 429 | Gm5497 | 3.200028125 | 5.098425878 | 1.593244084 |
| 430 | Tcte2 | 3.218992277 | 6.952091527 | 2.159710533 |
| 431 | Gm5921 | 3.219305409 | 6.952767801 | 2.159710533 |
| 432 | Enpp6 | 3.219618541 | 13.90688815 | 4.319421066 |
| 433 | Gm18949 | 3.272330873 | 3.707331299 | 1.132932898 |
| 434 | Fgf2os | 3.272330873 | 3.707331299 | 1.132932898 |
| 435 | Gm10044 | 3.274718564 | 3.710036393 | 1.132932898 |
| 436 | Adamts16 | 3.301380425 | 6.954796621 | 2.106632901 |
| 437 | Gm16486 | 3.309869266 | 5.097749604 | 1.540166452 |
| 438 | Rhag | 3.401241353 | 9.272385888 | 2.726176982 |
| 439 | LOC102638483 | 3.433800515 | 3.708007572 | 1.079855267 |
| 440 | Gm6225 | 3.433800515 | 3.708007572 | 1.079855267 |
| 441 | LOC102634137 | 3.433800515 | 3.708007572 | 1.079855267 |
| 442 | LOC102636870 | 3.433800515 | 3.708007572 | 1.079855267 |
| 443 | LOC102636169 | 3.491634348 | 5.563025769 | 1.593244084 |
| 444 | Gm10432 | 3.492058811 | 5.563702042 | 1.593244084 |
| 445 | Rps11-ps2 | 3.492058811 | 5.563702042 | 1.593244084 |
| 446 | LOC102633906 | 3.610646719 | 3.707331299 | 1.026777635 |
| 447 | 4933411E08Rik | 3.612403085 | 5.563702042 | 1.540166452 |
| 448 | Grhl3 | 3.612622629 | 3.709360119 | 1.026777635 |
| 449 | 3110009E18Rik | 3.63722523 | 11.58997516 | 3.186488168 |
| 450 | Frmd7 | 3.660036055 | 6.025596839 | 1.646321716 |
| 451 | Gm13858 | 3.683610693 | 4.173283737 | 1.132932898 |
| 452 | Rpl15-ps2 | 3.860911942 | 4.169226096 | 1.079855267 |
| 453 | Trim34b | 3.863103864 | 8.343186106 | 2.159710533 |
| 454 | 4930506C21Rik | 3.863416994 | 4.17193119 | 1.079855267 |
| 455 | Cfap44 | 3.864043257 | 4.172607463 | 1.079855267 |
| 456 | Sgca | 3.864043257 | 4.172607463 | 1.079855267 |
| 457 | Gm18890 | 3.864669521 | 4.173283737 | 1.079855267 |
| 458 | Gm15428 | 3.864669521 | 4.173283737 | 1.079855267 |
| 459 | LOC102637026 | 3.913180538 | 6.026949386 | 1.540166452 |
| 460 | Il1b | 4.07272448 | 6.488844183 | 1.593244084 |
| 461 | Jakmip3 | 4.213957992 | 6.49019673 | 1.540166452 |
| 462 | Wnt7b | 4.293033473 | 4.635854807 | 1.079855267 |
| 463 | Il2rb | 4.364726499 | 14.37148804 | 3.292643432 |
| 464 | 4632428C04Rik | 4.364755167 | 6.954120348 | 1.593244084 |
| 465 | Gm10269 | 4.721397426 | 5.098425878 | 1.079855267 |
| 466 | LOC100534358 | 4.722649952 | 5.099778425 | 1.079855267 |
| 467 | 4930573O16Rik | 4.786801364 | 7.880615036 | 1.646321716 |
| 468 | Ngb | 4.964803897 | 5.097749604 | 1.026777635 |
| 469 | LOC102640578 | 5.151013904 | 5.562349495 | 1.079855267 |
| 470 | Hao1 | 5.152266431 | 5.563702042 | 1.079855267 |
| 471 | Tmem56 | 5.319776129 | 6.026949386 | 1.132932898 |
| 472 | Kdm4d | 5.58188291 | 6.02762566 | 1.079855267 |
| 473 | Gm3650 | 6.012751914 | 6.492901824 | 1.079855267 |
| 474 | Dnmt3aos | 7.363639847 | 4.171254916 | 0.566466449 |
| 475 | Gm20472 | 8.122308535 | 4.16990237 | 0.513388817 |
| 476 | LOC102632163 | 8.130212174 | 4.17396001 | 0.513388817 |
| 477 | 4930415O20Rik | 8.183811795 | 4.635854807 | 0.566466449 |
| 478 | Shisa9 | 8.999208361 | 5.097749604 | 0.566466449 |
| 479 | LOC102634748 | 9.029909989 | 4.635854807 | 0.513388817 |
| 480 | A930015D03Rik | 10.6407461 | 6.02762566 | 0.566466449 |
| 481 | LOC102631905 | 10.83589199 | 5.563025769 | 0.513388817 |
