# Supplementary material for: H3K36 trimethylation mediated by SETD2 regulates the fate of bone marrow mesenchymal stem cells
Source: PLoS Biol. 2018 Nov 13;16(11):e2006522. doi: 10.1371/journal.pbio.2006522 (PMC6233919; doi:10.1371/journal.pbio.2006522)
Supplement: S2 Table — (DOCX) [file pbio.2006522.s003.docx]

| **Supplemental Table 2. Primers for RT-qPCR and ChIP-qPCR** | |
| --- | --- |
|  |  |
| **RT primers** |  |
| Setd2-RT-F: TAAGGGCTGCTAAGGATCTTCC; | Grem1-RT-R: TTGGCTGGACTCAAGCACCTC; |
| Setd2-RT-R: GTGGCATCTATTATCTCGTCATTTT; | Agt-RT-F: GGACCTGCTGGCTGAGGACAA; |
| Cebpα-RT-F: CAAGAACAGCAACGAGTACCG; | Agt-RT-R: CGTAGATGGCGAACAGGAAGG; |
| Cebpα-RT-R: GTCACTGGTCAACTCCAGCAC; | Lgr5-RT-F: GACGACCTTCATAAGAAAGACGC; |
| Pparγ1-RT-F: TGAAAGAAGCGGTGAACCACTG; | Lgr5-RT-R: GCTACCAAATAGGTGCTCACAGG; |
| Pparγ1-RT-R: TGGCATCTCTGTGTCCAACCATG; | Dlk2-RT-F: GTGAACTTGTCCTACCTGCTCCA; |
| Pparγ2-RT-F: GTTTTATGCTGTTATGGGTG; | Dlk2-RT-R: CCAAACACCACCAGGGCAACC; |
| Pparγ2-RT-R: GTAATTTCTTGTGAAGTGCTCATAG; | Tet1-RT-F: CATTCTCACAAGGACATTCACAACA |
| Fabp4-RT-F: GGGGCCAGGCTTCTATTCC; | Tet1-RT-R: AGTAAAACGTAGTCGCCTCTTCCTG |
| Fabp4-RT-R: GGAGCTGGGTTAGGTATGGG; | Fndc5-RT-F: ACAGAGCCCAGCCAGTGAGCCT; |
| Lpl-RT-F: AGGACCCCTGAAGACAC; | Fndc5-RT-R: TTGGGCTCGTTGTCCTTGATG; |
| Lpl-RT-R: GGCACCCAACTCTCATA; | Il1b-RT-F: CCTCGTGCTGTCGGACCCATA; |
| Perilipin-RT-F: AGATCCCGGCTCTTCAATACC; | Il1b-RT-R: CCATCTTCTTCTTTGGGTATTGC; |
| Perilipin-RT-R: AGAACCTTGTCAGAGGTGCTT; | Igfbp2-RT-F: AGCATGGCCGGTACAACCTTAA; |
| Hprt-RT-F: GTTAAGCAGTACAGCCCCAAA; | Igfbp2-RT-R: CCAGTCTCCTGCTGCTCGTTGT; |
| Hprt-RT-R: AGGGCATATCCAACAACAAACTT; | Hao1-RT-F: ATTGTTGAGGCTGTGGAAGGG; |
| Alp-RT-F: GGGCAACTCCATCTTTGGTCTG; | Hao1-RT-R: GACATCTTGAACACCTTTCTCCC; |
| Alp-RT-R: TTCACCGTCCACCACCTTGT; | Lbp-I1-F: GCTTCAGTTGAGTAGGGATAGTGG; |
| Col1a1-RT-F: GCTCCTCTTAGGGGCCACT; | Lbp-I1-R: ATCCCTCTGATGGAACGACACC; |
| Col1a1-RT-R: CCACGTCTCACCATTGGGG; | Lbp-I2-F: GAACTGATTGATGCTGCCCTCT; |
| Runx2-RT-F: ATGATGACACTGCCACCTCTGACT; | Lbp-I2-R: GCCTTCCCTGATTGCACCAT; |
| Runx2-RT-R: ATGAAATGCTTGGGAACTGCCTGG; | Lbp-I3-F: TGGGAAGTGGCTGTCTGTGC; |
| Osx-RT-F: CTCTGCTTGAGGAAGAAGCTCAC; | Lbp-I3-R: AAGGGCTGGAATGCTAATGG; |
| Osx-RT-R: CTTCTTTGTGCCTCCTTTCCC; | Lbp-I4-F: GAGGTGCCACTTCAACAAATCA; |
| Bsp-RT-F: GACTTTTGAGTTAGCGGCACT; | Lbp-I4-R: GGGAATGAAGACTGGGAGGC; |
| BSP-RT-R: CCGCCAGCTCGTTTTCATC; | Lbp-I5-F: ATTACCCTTGGCTACCCTGC; |
| PTX3-RT-F: GGTGGTGGGTGGAAAGGAGAA; | Lbp-I5-R: CGTGGTCACAGGTGGCTTCT; |
| PTX3-RT-R: GCCAATCTGTAGGAGTCCACCC; | Lbp-I7-F: ATAGAATCAGGAGGACCAGGAA; |
| Lbp-RT-F: CGTGGTCACTAATGTATTTGCCTCA; | Lbp-I7-R: CACCTGCTGAGGACTGTGACTGAA; |
| ;Lbp-RT-R: GCTCAGCATTGACATCAGGGTA; | Lbp-I15-F: TGCCACCTGGGAAGTGATTG; |
| Klf5-RT-F: CCAAACTGGCGATTCACAACC; | Lbp-I15-R: CCCAGGCTTCACATTCTTGC; |
| Klf5-RT-R: AAACCTCCAGTCGCAGCCCTCC; | CDC40-RT-F: AGAAGCGGAGAAGAACCAAGG; |
| Nr2f1-RT-F: TGGACCACATCCGCATCTTTCA; | CDC40-RT-R: TTTCTGCCTCTTTGCTGTGATT; |
| Nr2f1-RT-R: ATGTGGGCAGCATCCGACAGG; | DCP1A-RT-F: CTCGGCTCTGAGATCCAAGAT; |
| Grem1-RT-F: CACCGCATACACTGTGGGAGC; | DCP1A-RT-R: CGGTTGACAATGGTAAAGCCA. |
| **Chip-Primers** |  |
| Lbp-C1-F: CCAGCGGGACCAGTAGATTGC; | Lbp-C2-F: TCTCAACCAATAAATGACAACCCTA; |
| Lbp-C1-R: GGTTTCTCCTGGCTGCTTTCA; | Lbp-C2-R: CGACTTAGAGGGAGACAGGCAC. |
