# Supplementary figures and images for: H3K36 trimethylation mediated by SETD2 regulates the fate of bone marrow mesenchymal stem cells
Source: PLoS Biol. 2018 Nov 13;16(11):e2006522. doi: 10.1371/journal.pbio.2006522 (PMC6233919; doi:10.1371/journal.pbio.2006522)

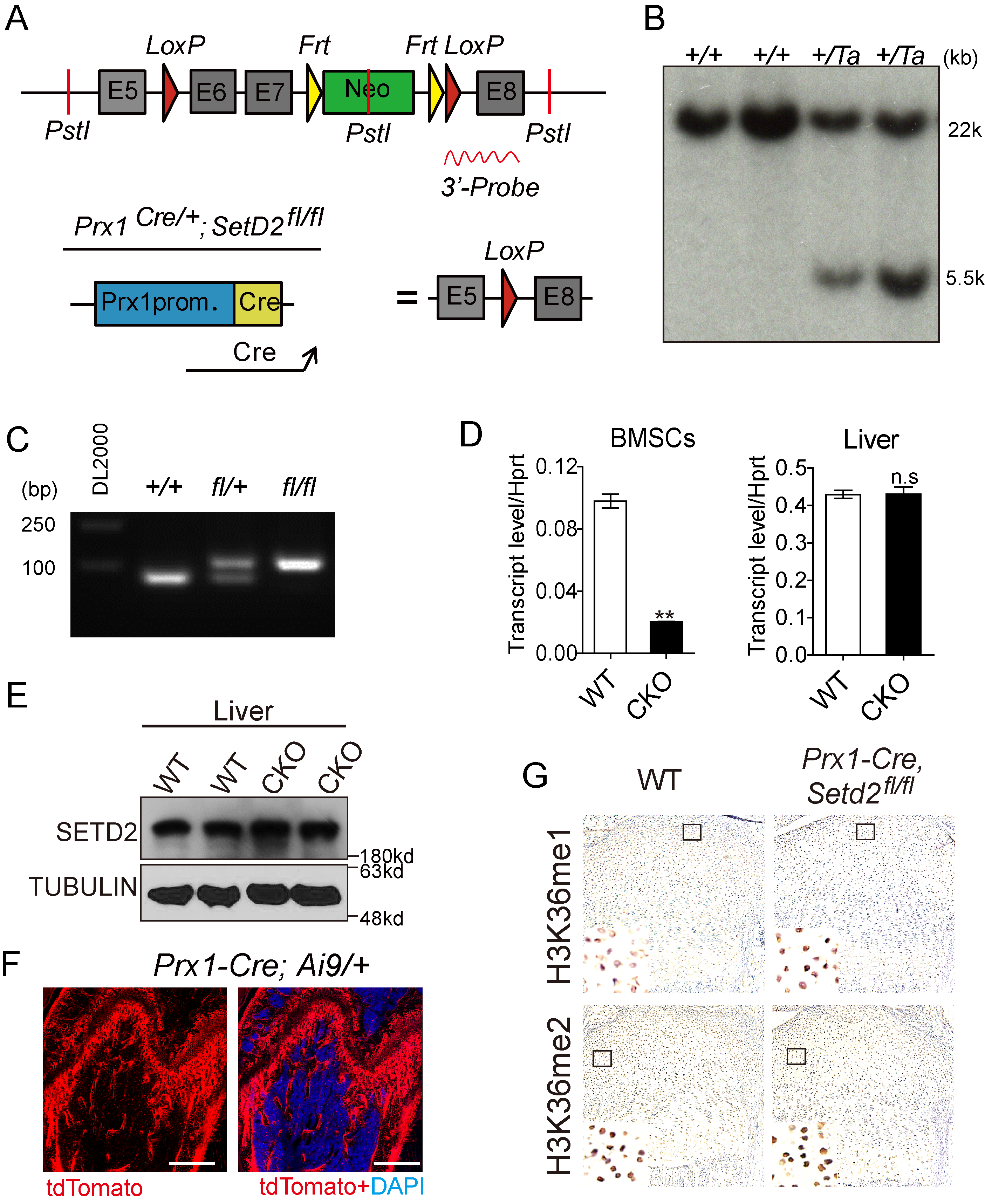

Supplement: S1 Fig — (A) Prx1-Cre, Setd2fl/fl mice construction strategy. (B) Southern blot of Setd2 after PstI digestion, target allele was digested into 2 segments with length of 5k and 22k. (C) Genotyping of WT, Setd2fl/+ and Setd2fl/fl mice. (D) qPCR analysis of Setd2 expression in mBMSCs and liver from WT and Prx1-Cre, Setd2fl/fl mice. Results are presented as the mean ± SD, n = 4 per condition. (E) Western blot analysis of SETD2 in liver. (F) Representative image by fluorescence microscopy from the femur shown prx1 positive cells using Prx1-Cre, Ai9/+ mice. Scale bar = 1 mm. (G) Immunohistochemistry assay of H3K36me1/2 level in hindlimb growth plate of Prx1-Cre, Setd2fl/fl mice and WT control mice. Data used in the generation of this figure can be found in S1 Data. (TIF) [file pbio.2006522.s004.tif]

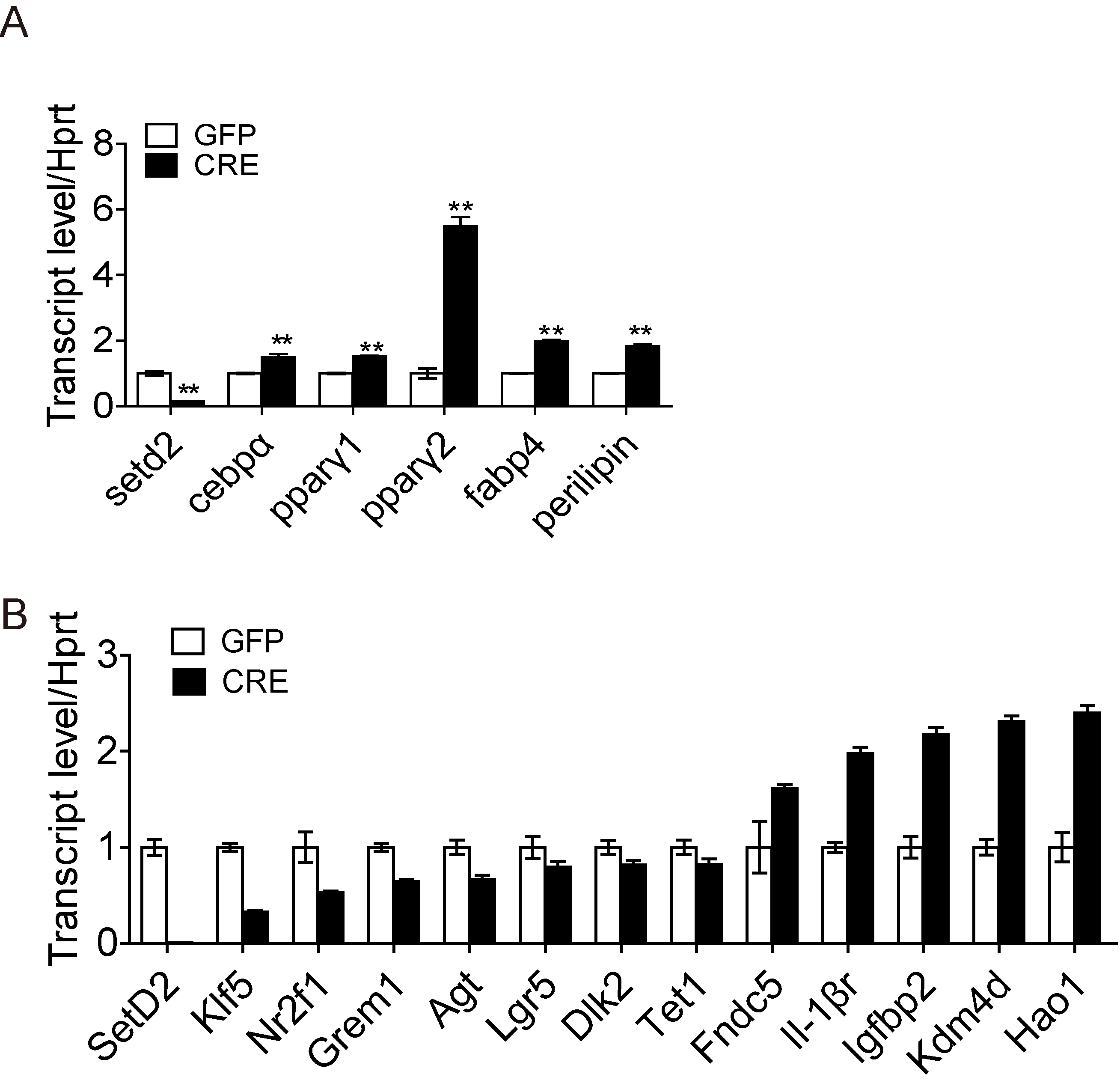

Supplement: S2 Fig — (A) Analysis of Setd2, Pparγ1, Pparγ2, Cebpα, Fabp4, and Perilipin via qPCR of BMSCs isolated from Setd2fl/fl mice treated with Cre and GFP lentivirus induced by adipogenesis medium for 6 days. Results are presented as the mean ± SD, n = 4 per condition. (B) Relative expression of differential genes in the control (GFP) versus Setd2-deficient (Cre) BMCSs. Results are presented as the mean ± SD, n = 4 per condition. Data used in the generation of this figure can be found in S1 Data. (TIF) [file pbio.2006522.s005.tif]

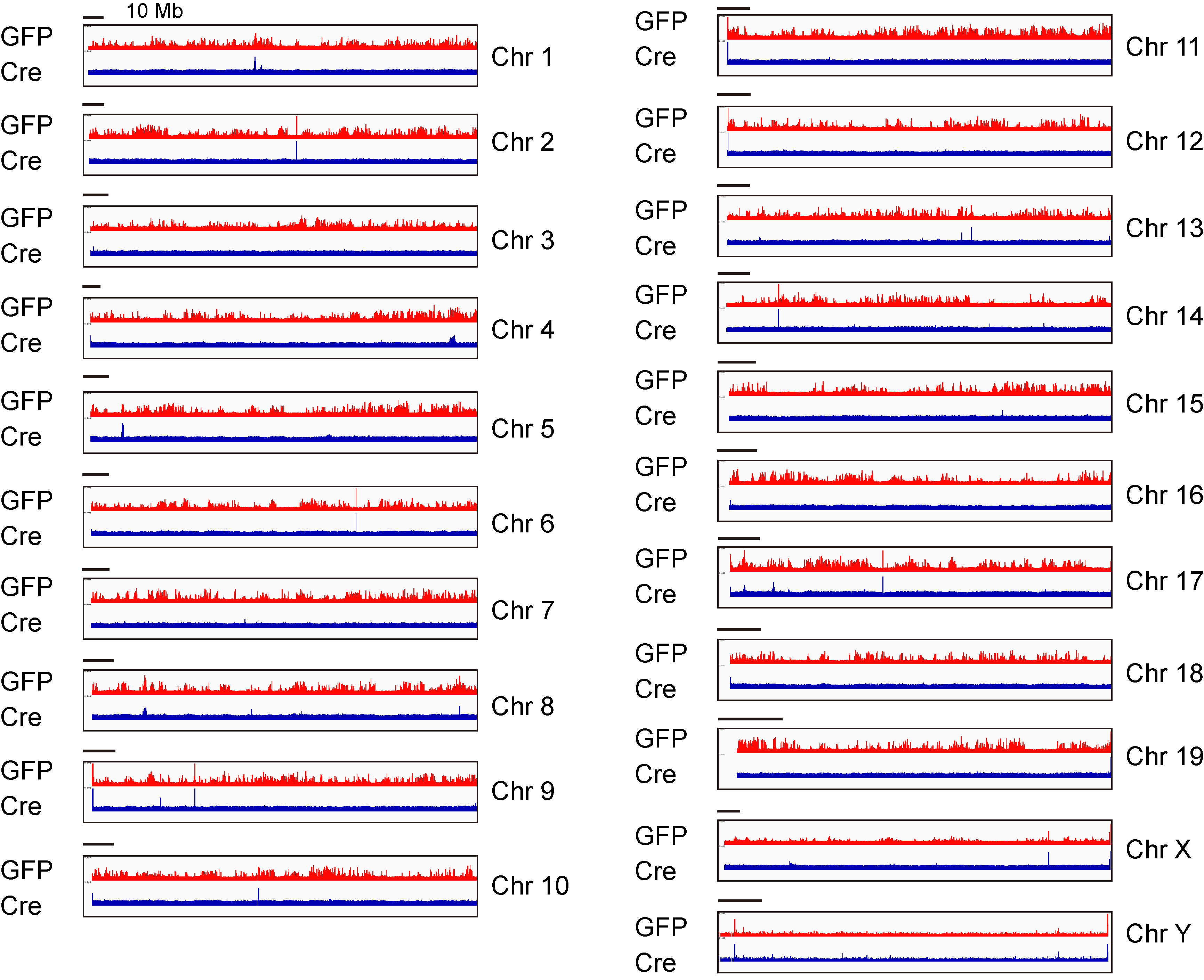

Supplement: S3 Fig — The black bars on top of each panel show 10-kb scale. All panels have the same signal scale of 0–5 RPM on the y-axis. (TIF) [file pbio.2006522.s006.tif]

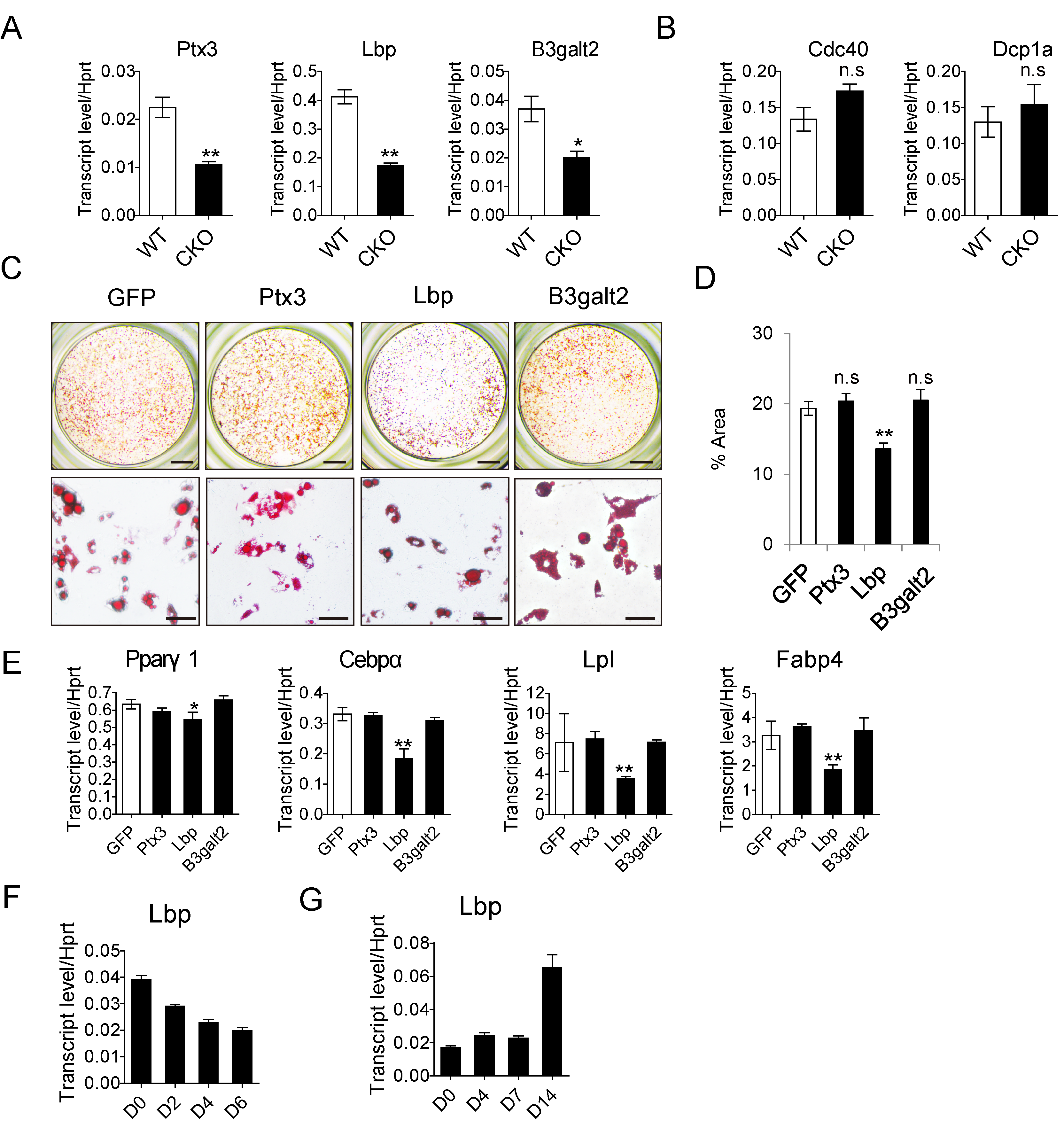

Supplement: S4 Fig — (A, B) Relative expression levels of indicated genes in WT and Prx1-Cre, Setd2fl/fl mice. Results are presented as the mean ± SD, n = 4 per condition. (C) Morphological image of BMSCs at day 6 induced by adipogenesis medium, BMSCs were infected with lentivirus expressing GFP, Ptx, Lbp, and B3galt2. Cells were stained with Oil Red O. Upper panels, stained dishes, scale bar = 1 mm; lower panels, representative fields under the microscope, scale bar = 100 μm. (D) Quantitative analysis of Oil Red staining. Results are presented as the mean ± SD, n = 4 per condition. (E) Expression analysis of indicated genes. Results are presented as the mean ± SD, n = 4 per condition. (F–G) qPCR analysis of Lbp during adipogenesis (panel F) and osteogenesis (panel G). Data used in the generation of this figure can be found in S1 Data. (TIF) [file pbio.2006522.s007.tif]

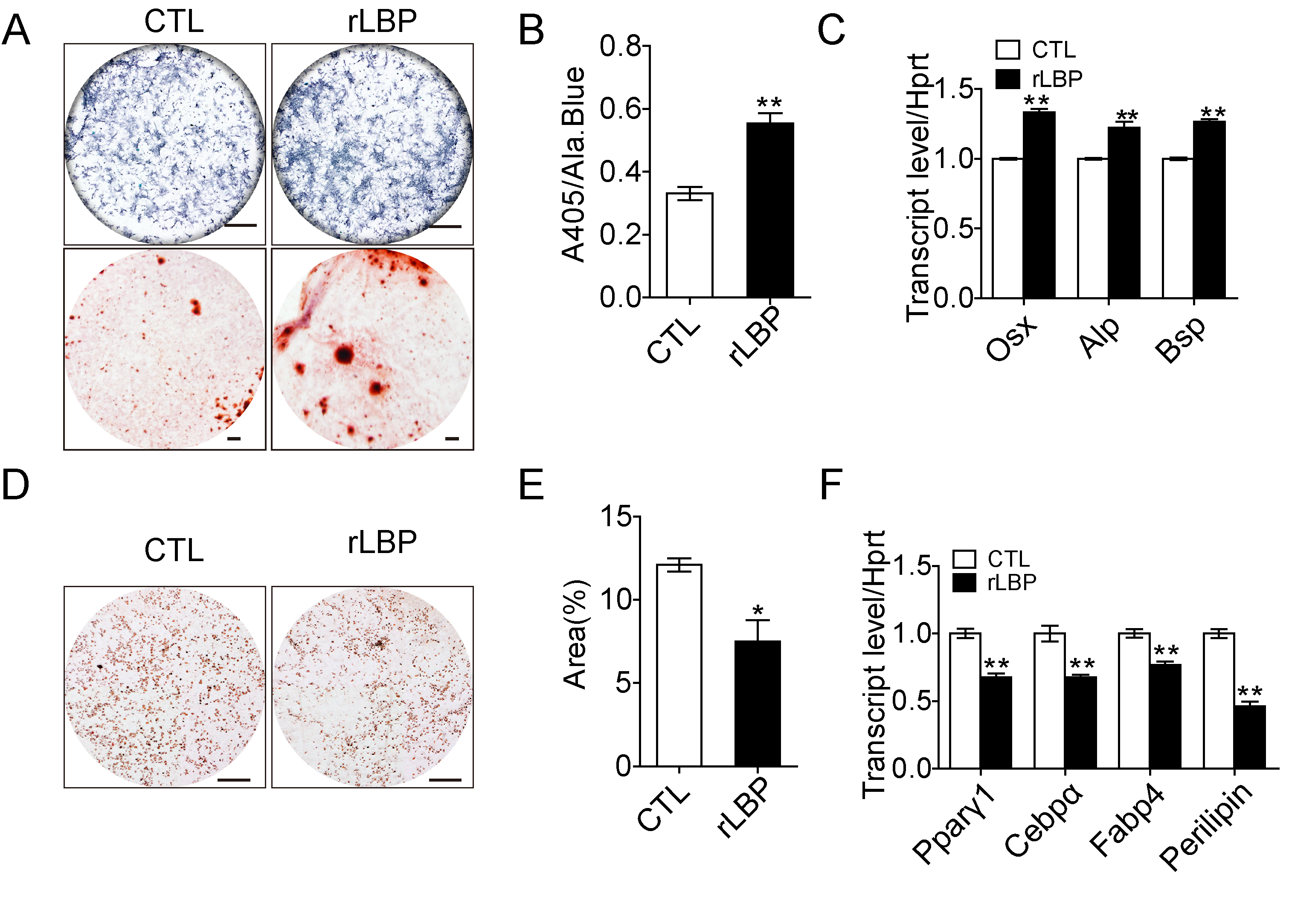

Supplement: S5 Fig — (A) Alp activity and Alizarin red S staining after osteoblast differentiation for 7 days (upper) and 21 days (lower), respectively, with rLBP treatment. Scale bar = 1 mm. (B) Alp activity quantification was measured by phosphatase substrate assay. The results are represented as mean ± SD, n = 4 for each treatment. (C) qPCR analysis of Osx, Alp, and Bsp expression after osteoblast differentiation for 7 days with rLBP administration; cells were from WT mBMSCs. (D) Oil Red O staining after adipogenesis for 6 days, scale bar = 1 mm. (E) Quantitative analysis of Oil Red O staining, the results are represented as mean ± SD, n = 3. (F) Expression analysis of indicated genes, including Pparγ1, Cebpα, Fabp4, and Perilipin. Data used in the generation of this figure can be found in S1 Data. (TIF) [file pbio.2006522.s008.tif]

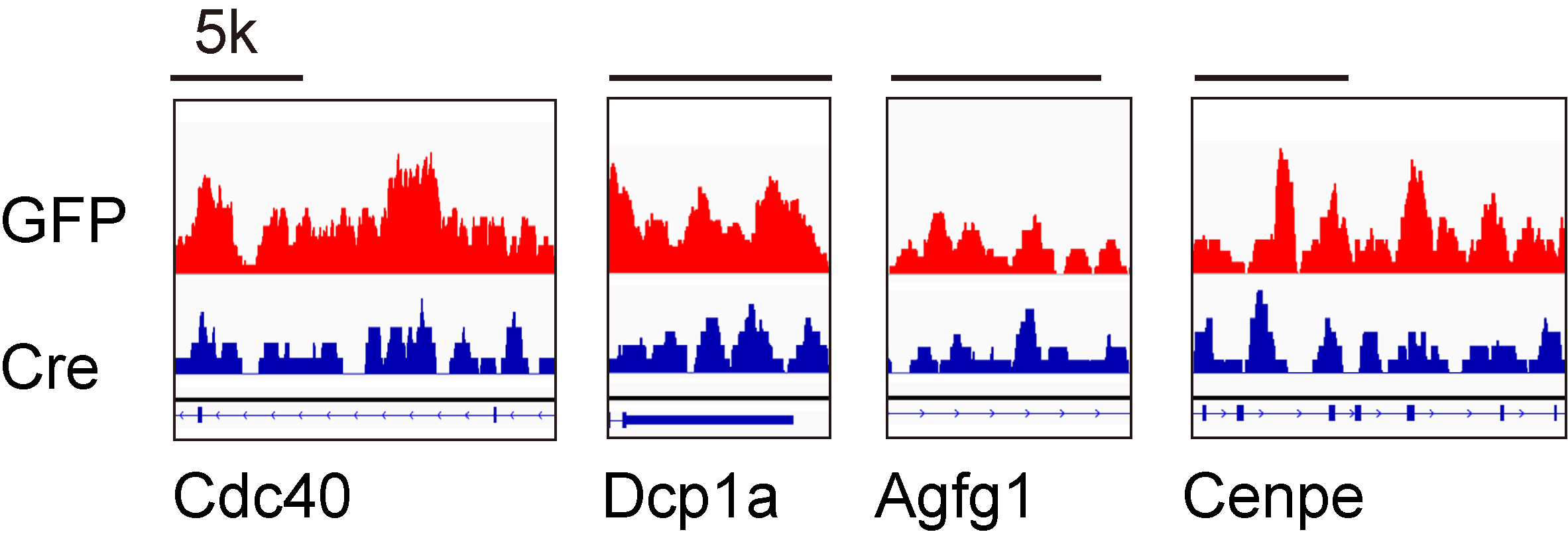

Supplement: S6 Fig — ChIP-seq profiles of indicated fundamental genes by H3K36me3 antibody shown in Integrated Genomic Viewer. The black bars on top of each panel show 5-kb scale. All panels have the same signal scale of 0–5 RPM on the y-axis. The expanded RefSeq gene is shown below each panel. (TIF) [file pbio.2006522.s009.tif]

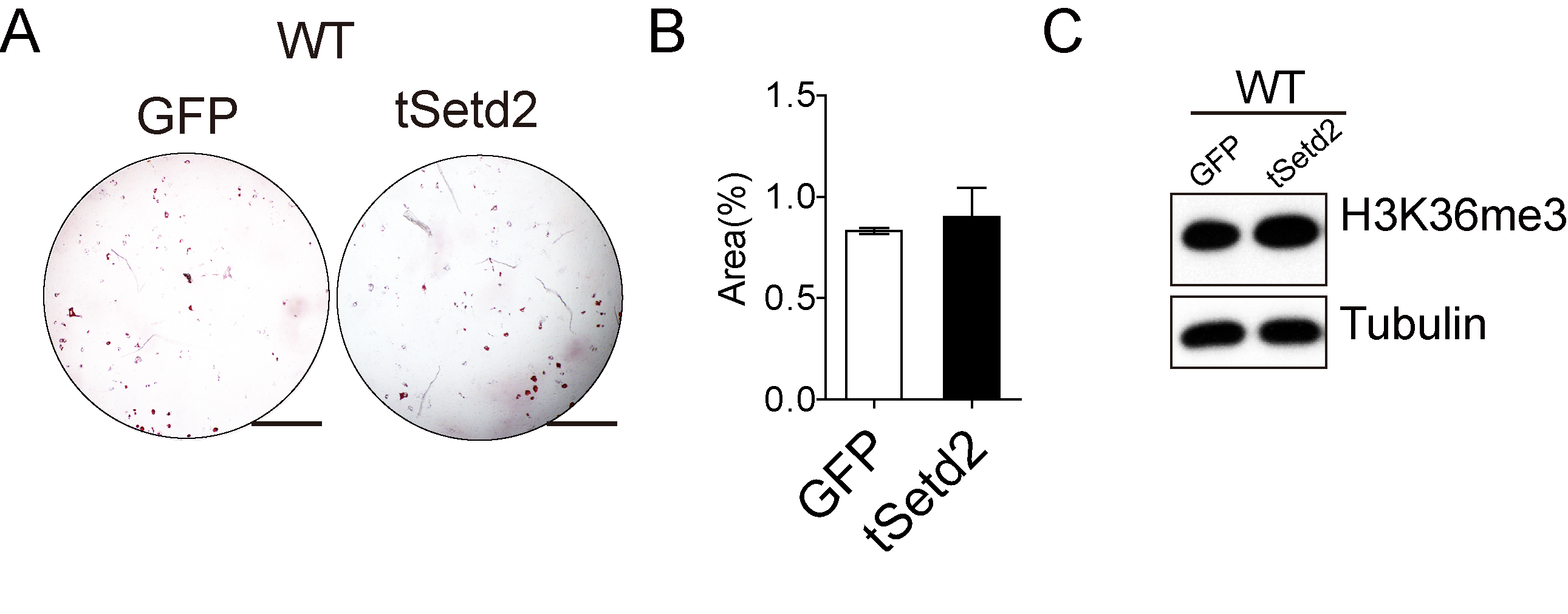

Supplement: S7 Fig — (A–B) Oil Red O staining and quantification of adipocytes differentiated from mBMSC which were infected with lentivirus expressing GFP and tSetd2 followed by adipocyte differentiation for 6 days, scale bar = 1 mm. (C) H3K36me3 levels in WT cells infected with lentivirus expressing GPF and tSetd2. Data used in the generation of this figure can be found in S1 Data. (TIF) [file pbio.2006522.s010.tif]

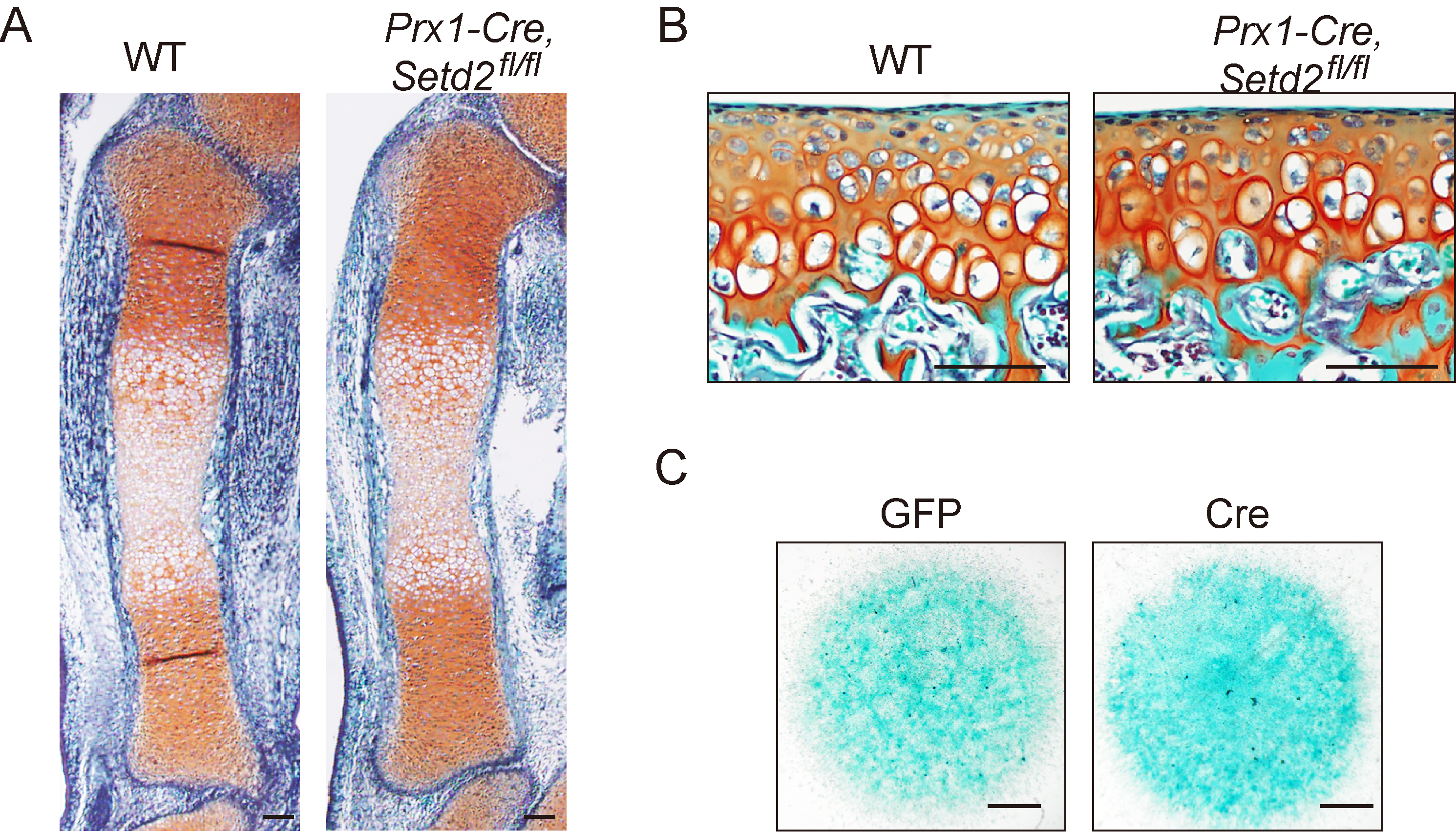

Supplement: S8 Fig — (A) Safranin O staining at embryonic day 16.5 in WT and Prx1-Cre; Setd2fl/fl mice, scale bar = 100 μm. (B) Safranin O staining at 5 weeks at the cartilage, scale bar = 100 μm. (C) Alcien blue staining for micromass culture at D7; chondrocyte progenitors were isolated from Setd2fl/fl mice at P3 and infected with GFP and Cre-lentivirus, scale bar = 1 mm. (TIF) [file pbio.2006522.s011.tif]

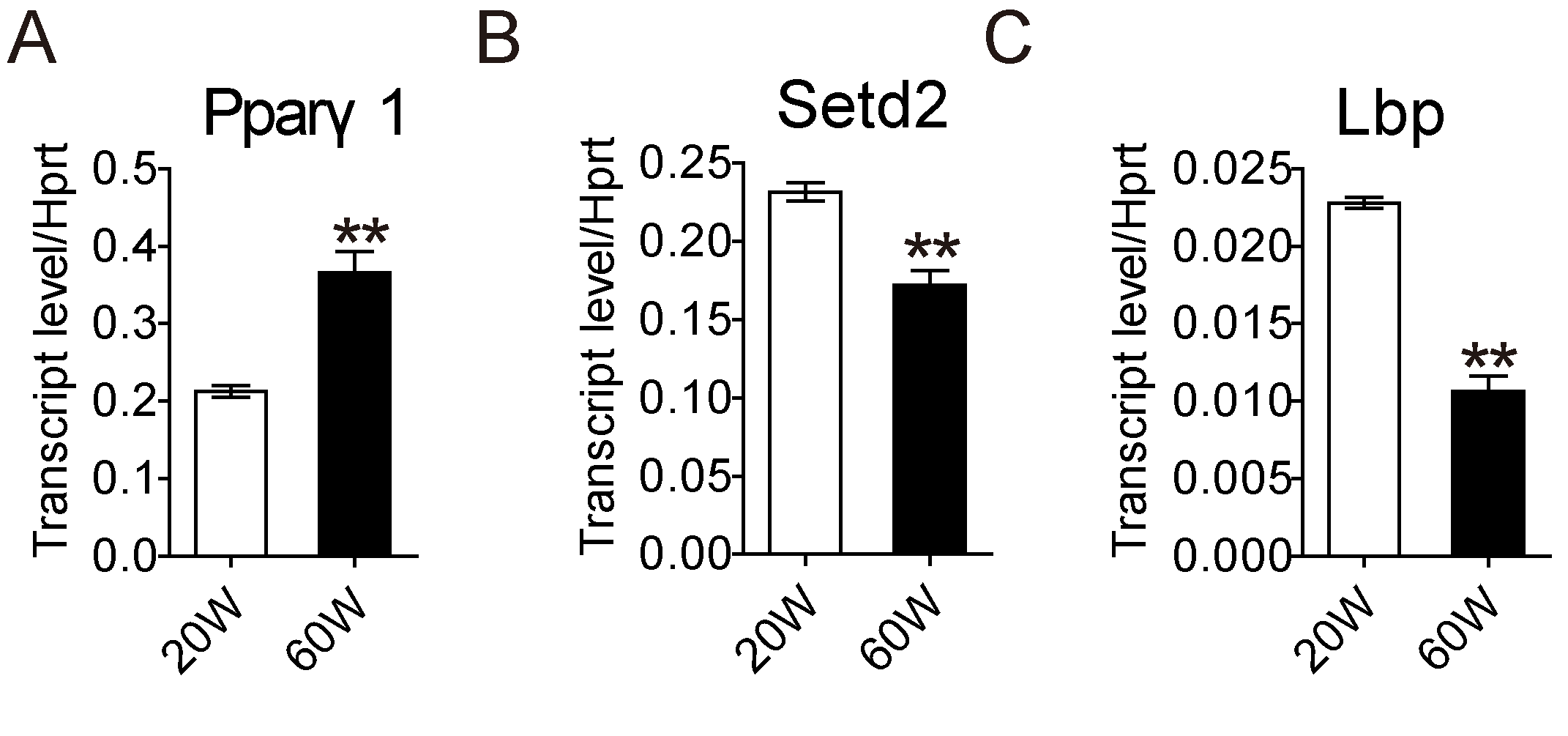

Supplement: S9 Fig — (A–C) Analysis of Pparγ1, Setd2, and Lbp via qPCR of BMSCs isolated from 20-week and 60-week WT mice. Results are presented as the mean ± SD, n = 3 mice per condition. Data used in the generation of this figure can be found in S1 Data. (TIF) [file pbio.2006522.s012.tif]

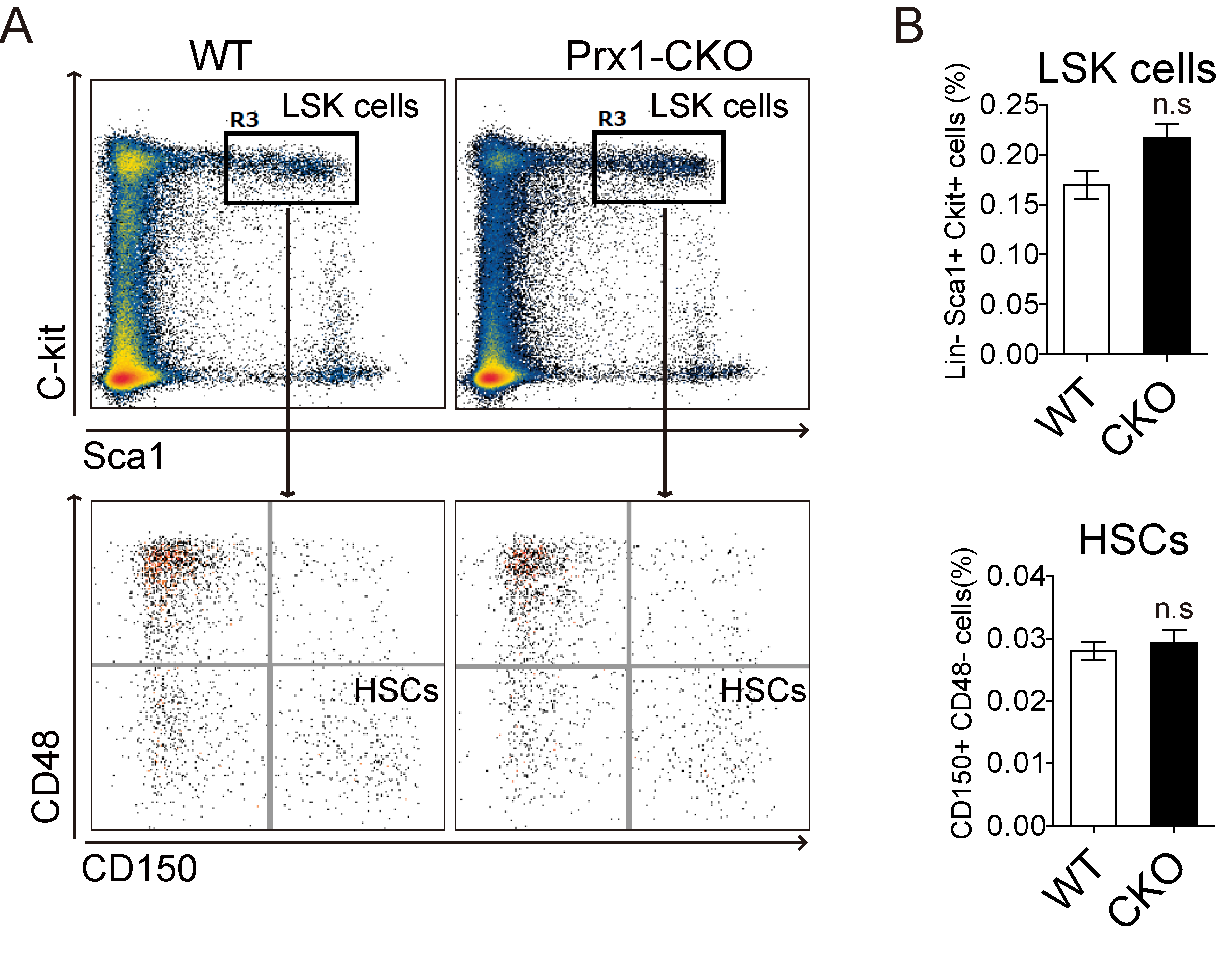

Supplement: S10 Fig — (A) Flow cytometric analysis of Lineage-Sca-1+ c-kit+ LSK cells and CD150+ CD48− Lineage-Sca-1+ c-kit+ HSCs of bone marrow cells that are from WT and Prx1-Cre; Setd2fl/fl mice. (B) Quantification of LSK cells and HSCs. Results are presented as the mean ± SD, n = 3 per condition. Data used in the generation of this figure can be found in S1 Data. (TIF) [file pbio.2006522.s013.tif]

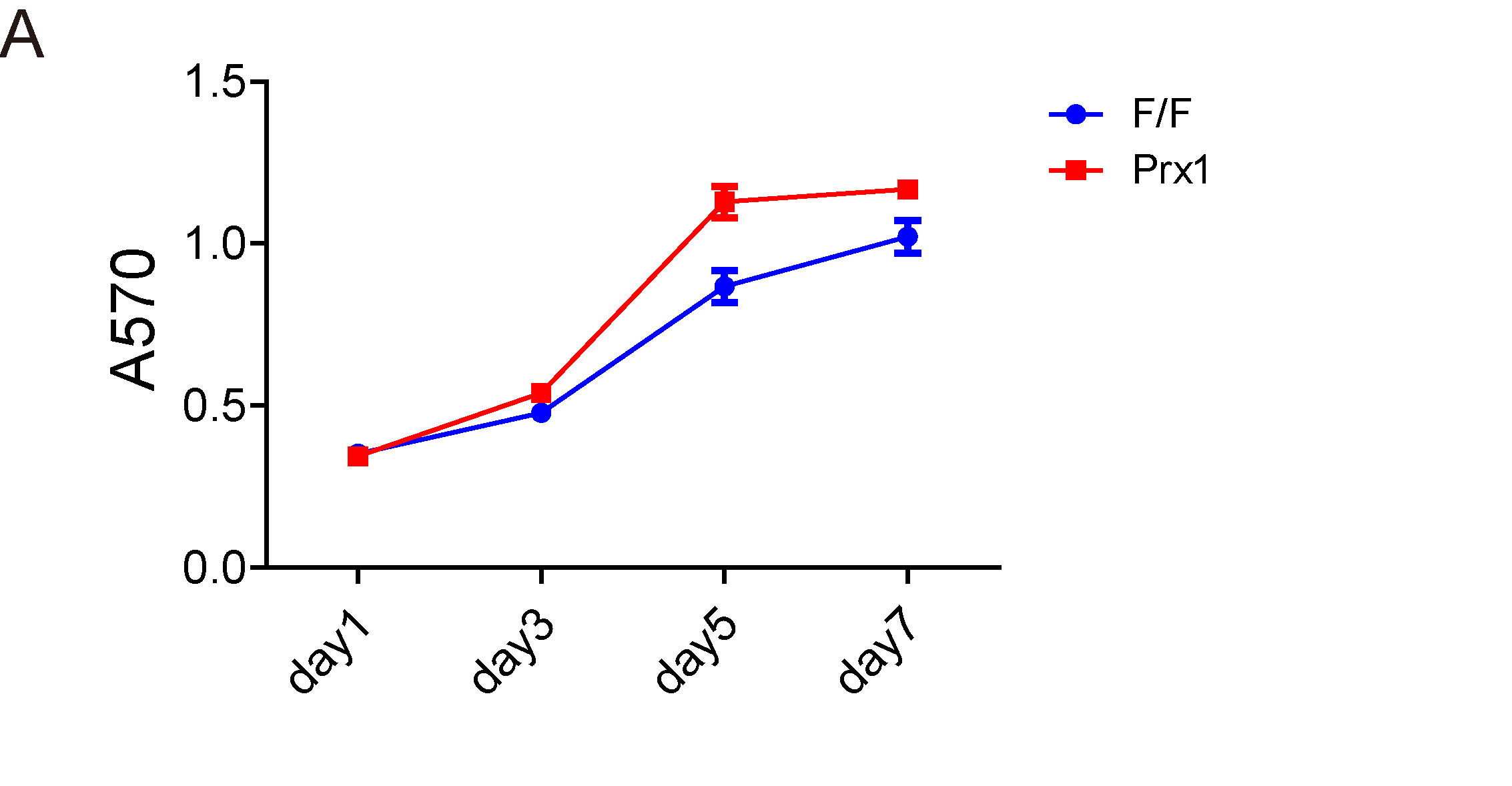

Supplement: S11 Fig — Data used in the generation of this figure can be found in S1 Data. (TIF) [file pbio.2006522.s014.tif]
